# The Human Circadian Clock Protein Cryptochrome‑1 Responds to Photochemistry by Conformational Changes

**DOI:** 10.1021/jacs.6c06484

**Published:** 2026-07-31

**Authors:** Axel Jeibmann, Mian Qi, Marten Hellmich, Adelheid Godt, Tilman Kottke, Lukas Goett-Zink

**Affiliations:** † Biophysical Chemistry and Diagnostics, Medical School OWL, 9167 Bielefeld University, Universitätsstraße 25, Bielefeld 33615, Germany; ‡ Biophysical Chemistry and Diagnostics, Faculty of Chemistry, Bielefeld University, Universitätsstraße 25, Bielefeld 33615, Germany; § Organic and Macromolecular Chemistry, Faculty of Chemistry, Bielefeld University, Universitätstraße 25, Bielefeld 33615, Germany

## Abstract

The human clock protein cryptochrome-1 (HsCRY1) can be targeted by small molecular modulators to shift the 24 h rhythm, opening up possibilities for treating circadian disorders. GO1323 is a photopharmacological compound that selectively binds to HsCRY1 and enables light-controlled manipulation of the circadian rhythm via photoisomerization. We synthesized isotope-labeled and unlabeled GO1323 to investigate conformational changes in full-length HsCRY1 that are caused by isomerization of GO1323 using infrared difference spectroscopy. Activation of GO1323 induces specific changes in secondary structure including a rearrangement of the β-sheet, which are surprisingly similar to those observed in the activation of evolutionarily separated plant cryptochromes by flavin photoreduction. Accordingly, we investigated the response of HsCRY1 to flavin photochemistry despite the reported low affinity to oxidized flavin. We revealed changes in secondary structure only during the transition from the flavin neutral radical to the fully reduced flavin, which requires the sequential absorption of two photons. Difference spectra suggest a similar HsCRY1 activation by GO1323 isomerization and by flavin photoreduction. Therefore, we present a model in which HsCRY1 acts as a bifunctional protein, responding to interaction partners and flavin photoreduction as a high light receptor.

## Graphical abstract

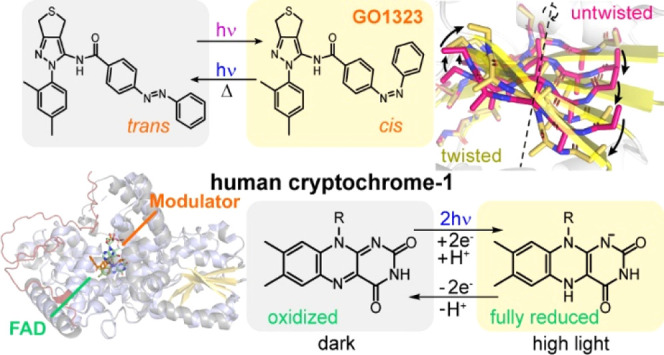

## Introduction

Human daily life is determined by the Earth’s rotational schedule. Nature has evolved an internal clock that aligns our physiological behavior such as bedtime, activity, body temperature and appetite to a 24 h day–night cycle.^1^ The vast majority of the human proteome is regulated in a circadian rhythm.^2^ The central pacemaker for the circadian rhythm is the suprachiasmatic nucleus (SCN), based on transcription-translation feedback loops (TTFL) with a periodicity of approximately 24 h.^3^ The SCN is entrained by the photoreceptor melanopsin located in the eye and synchronizes the peripheral cellular clocks in all tissues of the human body.^3,4^

Cryptochrome proteins (CRYs) act as key repressors in mammalian TTFL. They assemble to macromolecular complexes with period proteins (PER) and the casein kinase 1δ isoform in the cytosol.^5^ These CRY-containing complexes enter the nucleus in the evening, where they bind and repress the two transcription factors circadian locomotor output cycles kaput (CLOCK) and brain and muscle ARNT-like 1 (BMAL1) responsible for CRY and PER expression.^5,6^ During the night, the repressive complexes are modified by post-translational modifications resulting in a late repressive complex with cryptochrome-1 bound to CLOCK and BMAL1.^7–9^ Then, a new cycle of TTFL is started in the morning by degradation of cryptochrome-1 (CRY1), which plays a unique role in phase determination of vertebrate circadian clocks.^5^

CRYs are part of the cryptochrome/photolyase superfamily. They share a conserved photolyase homology region (PHR) of about 500 amino acids and an unstructured C-terminal extension (CCE) of variable length (Figure 1A). Most CRYs bind flavin adenine dinucleotide (FAD) to the PHR domain with subnanomolar affinity as a chromophore to function as photoreceptors.^10,11^ In contrast, animal type II CRYs, including mammalian CRYs, bind FAD^12^ with a low, micromolar affinity (*K*_D_ ∼16–97 μM).^13–15^ Moreover, functions of ocular photoreceptors in mice have been assigned to opsins instead of CRYs.^16^ Recent studies demonstrate that FAD binding to PHR protects mammalian CRYs against degradation and thereby contributes to regulation of cellular metabolism.^17^ Human cryptochromes have been shown to undergo photoreduction in *Drosophila* cells and to rescue light-dependent magnetoreception in *Drosophila* cryptochrome (dCRY) null mutants.^18,19^ Any light-dependent roles of mammalian CRYs remain unclear. In particular, structural responses of mammalian CRYs to FAD photoreduction have not been resolved.

**Figure 1:**
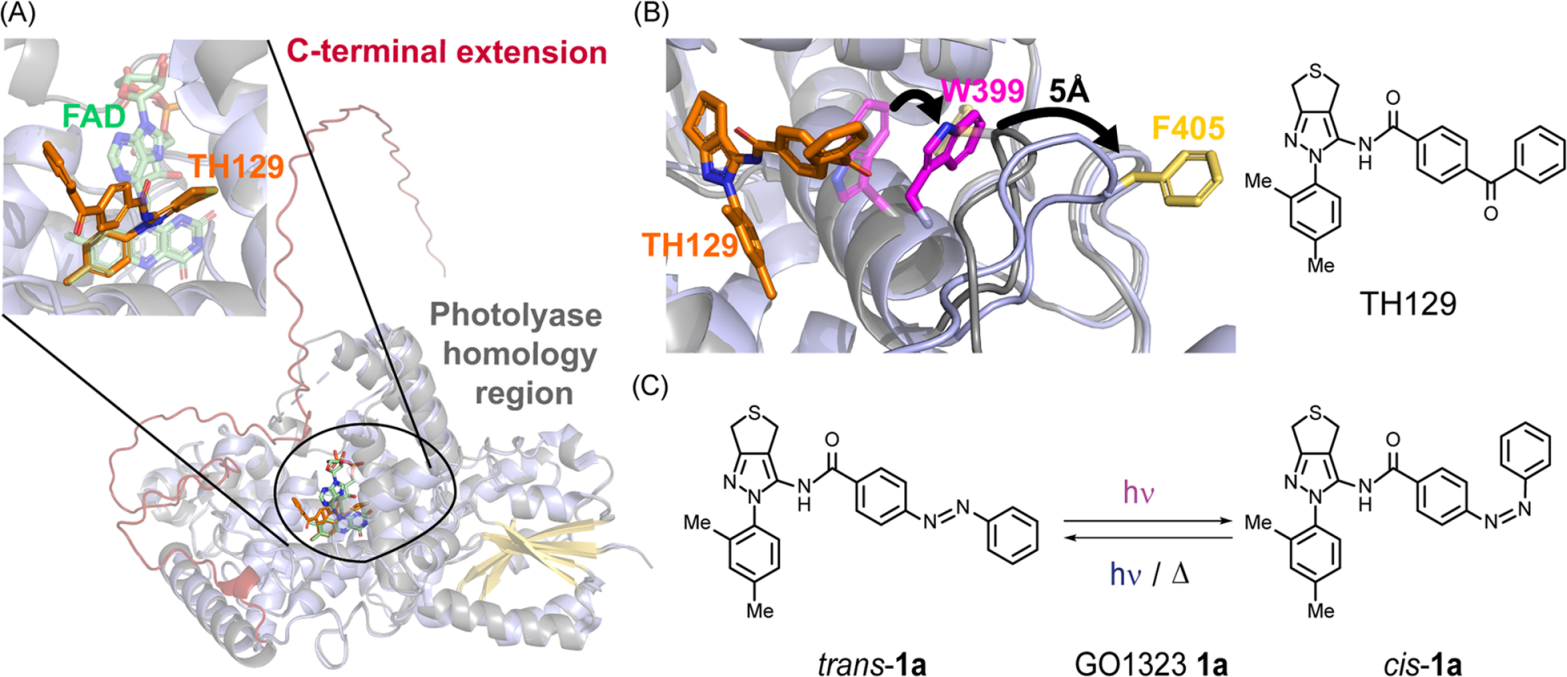
Structures of mammalian cryptochrome-1 and the light-switchable modulator GO1323 (**1a**). (A) Overlay of the FAD-bound AlphaFold3 model (gray) and the crystal structure of TH129 bound to mouse cryptochrome-1 (PDB code: 7D19, light blue). Modulator TH129 (orange) occupies the binding pocket of the cofactor FAD (green) in the PHR. (B) Overlay of crystal structures of mouse cryptochrome-1 with and without TH129 (PDB code: 6KX4). Binding of TH129 leads to a 5 Å outward shift of the lid loop. (C) Light-induced *trans*–to-*cis* isomerization of photopharmacological compound GO1323 (**1a**), which specifically targets human cryptochrome-1. The *cis*-form of GO1323 mimics the TH129 modulator in structure and activity.

Misalignment of the circadian clock has a severe impact on human health including metabolic dysfunction, cognitive impairment and cardiovascular abnormalities.^20,21^ Pharmacological compounds that manipulate the human circadian clock may help to treat circadian rhythm disorders. TH129 is an isoform-specific agonist, which selectively binds to the cofactor-binding pocket of human cryptochrome-1 (HsCRY1) (Figure 1A). Binding of TH129 activates HsCRY1 resulting in a prolonged lifetime of the protein and a period lengthening of the circadian clock.^22^ The phase shifting effect of TH129 has been explained by a local displacement of W399 and F405 in a region called lid loop (Figure 1B), as observed by X-ray crystallography of the PHR of mouse cryptochrome-1.^15,22^ This region is located at the interaction interface with a ubiquitin ligase F-box/LRR-repeat protein 3 (FBXL3).^23^ Kolarski *et al.* designed the photopharmacological compound GO1323 (**1a**), which is structurally closely related with TH129.^22^ Compound **1a** possesses an azobenzene group and its configuration can be switched between *trans*- and *cis*-form by UV and blue light (Figure 1C). The effect of the *cis*-form mimics the cellular activity of TH129. Switching the nonactive *trans*-**1a** to the active *cis*-**1a** and back with light provides access to a reversible and noninvasive regulation of TTFL.^22^ The activation mechanism of HsCRY1 by TH129 and **1a** leading to a period lengthening of TTFL is only partially understood.^33^ The conformational changes in HsCRY1 due to **1a** switching have so far been sparsely characterized.

Controlling the activity of **1a** in HsCRY1 with light enables the application of Fourier transform infrared (FTIR) difference spectroscopy, a well-established technique that is highly sensitive to changes in protein secondary structure, amino acid side chains and cofactors. Light-induced FTIR difference spectroscopy has been successfully employed to resolve protein mechanisms, including members of the cryptochrome/photolyase superfamily,^24,25^ photosystem II,^26,27^ phytochromes,^28,29^ and rhodopsins.^30–32^

Here, we synthesized both isotope-labeled and unlabeled variants of the photopharmacological compound **1** and activated it with light while bound to full-length HsCRY1. We revealed specific changes in the secondary structure of HsCRY1 that go beyond a local shift in the lid loop. These findings motivated us to investigate the structural response of HsCRY1 to the photoreduction of FAD, which has to our knowledge not been resolved previously. The remarkable similarities between FTIR difference spectra of HsCRY1 with FAD and **1a** prompt us to consider the notion of a light-dependent function of HsCRY1 in addition to its light-independent role as clock component.

## Results

### Synthesis of Isotope-Labeled and Unlabeled Photopharmacological Compounds

The investigation of protein mechanisms via FTIR difference spectroscopy requires an external stimulus for switching between different states. Recently, the development of the photopharmacological compound **1a** was reported which selectively targets HsCRY1.^22^ Its azobenzene group can be switched by UV and blue light between its *cis*- and *trans*-configuration corresponding to the pharmacologically active and inactive state, respectively.^22^ Therefore, we synthesized **1a** and the corresponding isotope-labeled compound **1b** for studying light-induced structural changes of HsCRY1 (Scheme 1). Compound **1a** with natural isotopic abundance was assembled from pyrazolamine **5** ^33^ and azobenzenecarboxylic acid **4a** (Scheme 1) on the basis of a reported procedure.^22^ Azobenzenecarboxylic acid (**4a**) was obtained from methyl 4-aminobenzoate (**2a**) via a Baeyer–Mills reaction^34,35^ with nitrosobenzene^36^ and a subsequent ester hydrolysis. Acylation of pyrazolamine **5** with azobenzoic acid **4a** yielded photopharmacological compound **1a**.

**Scheme 1:**
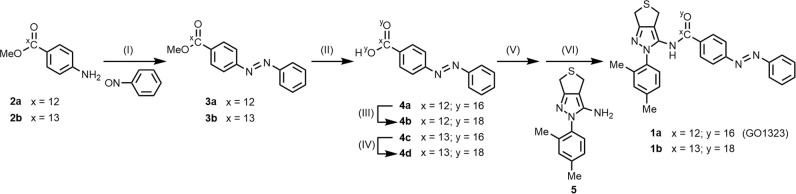
Syntheses of Photopharmacological Compound **1a** and Corresponding Isotope-Labeled Compound **1b**.

(I) **2a**→ **3a**: HOAc, rt, 24 h, 78%; **2b**→ **3b**: HOAc, rt, 17.5 h, 98%; (II) **3a**→ **4a**: (1) LiOH, H_2_O, THF, rt, 4 h, (2) HCl, H_2_O, 99%; **3b**→ **4c**: (1) LiOH, H_2_O, THF, rt, 4 h, (2) HCl, H_2_O, 97%; (III) experiment 1: H_2_^18^O, EtN=C–N­(CH_2_)_3_NMe_2_•HCl (EDC•HCl), 3,5-dimethylpyridine hydrobromide, DMF, rt, 7 d, 43% ^18^O-enrichment; experiment 2: H_2_^18^O, EDC•HCl, 3,5-dimethylpyridine hydrobromide, DMF, rt, 2 d, then 40 °C, 3 d, 48% ^18^O-enrichment; (IV) (1) H_2_^18^O, HCl, 1,4-dioxane, 100 °C, 17 h, (2) freeze-drying, treatment applied four times, 97% ^18^O-enrichment, yield 92%; **4a**→ **1a**: (V) (1) SOCl_2_, 80 °C, 1.5 h, (2) removal of all volatiles, (VI) Et_3_N, CH_2_Cl_2_, rt, 21 h, 39%; **4d**→ **1b**: (V) (1) SOCl_2_, 80 °C, 1.5 h, (2) removal of all volatiles; (VI) Et_3_N, CH_2_Cl_2_, rt, 18 h, 99% ^13^C-enrichment, 97% ^18^O-enrichment, yield 23%.

Compound **1b** was synthesized in the same way, only with the addition of an esterification of commercially available 4-aminobenzene­[^13^C]­carboxylic acid and an exchange of the ^16^O atoms for ^18^O atoms at the stage of azobenzene­[^13^C]­carboxylic acid **4c**. The ^16^O/^18^O exchange using a nonequilibrium, multiple–turnover reaction reported by Seyfried et al.^37^ was not sufficient, whereas high enrichments of up to 97% were achieved using an equilibrium-based reaction.^38^ Synthesis of isotope-labeled **1b** was obtained with a 99% ^13^C-enrichment, 97% ^18^O-enrichment and a yield of 23%.

### Rearrangement of the β-Sheet in HsCRY1 Induced by Photochemically Activating the Circadian Clock Modulator

The functionality of the reversible switching of **1a** between *trans*- and *cis*-configuration was checked by nuclear magnetic resonance in DMSO-*d*_6_ (Figure S1 and Table S1) and UV–vis spectroscopy in DMSO (Figure 2A). Illumination with UV-light at 385 nm for 120 s led to an increase of absorbance at ∼280 nm and a pronounced loss at 335 nm, corresponding to π → π* transitions, as well as a slight blue shift at 400–500 nm of the *n* → π* transition, consistent with the photoisomerization properties of azobenzene.^39^ The spectral changes were completely reversed by illuminating the sample with blue light at 410 nm for 30 s (Figure 2A inset). Accordingly, **1a** can be switched by light between two photostationary states, while thermal isomerization can be neglected because of the long half-life of *t*_1/2_ > 1 d of *cis*-**1a**.^22^ FTIR difference spectra of **1a** in DMSO revealed the characteristic fingerprint of the isomerization (Figure S2A).

**Figure 2:**
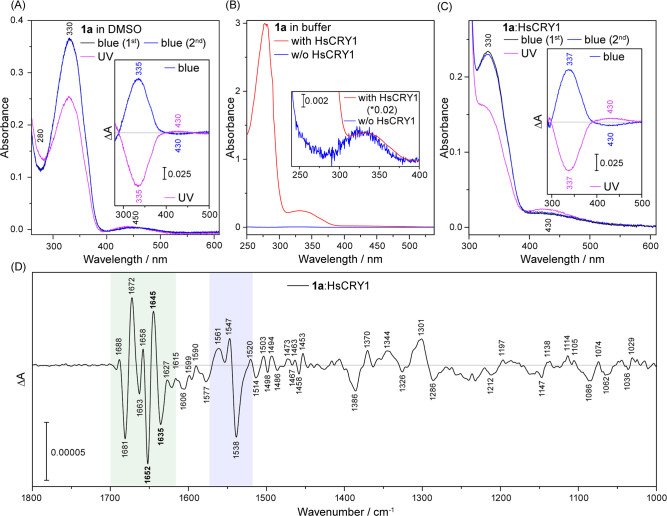
UV–vis and infrared difference spectra of the photoisomerization of **1a** and **1a**:HsCRY1. (A) UV–vis spectra of **1a** in DMSO after illumination with 385 nm UV light and 415 nm blue light show reversible photoinduced isomerization from the *trans*- to the *cis*-configuration and back as demonstrated by a loss and gain of absorbance at 335 nm, respectively (inset). (B) Dissolving **1a** in buffer and subsequent centrifugation results in very low absorbance at 340 nm, demonstrating that **1a** is highly insoluble in water. In contrast, the absorbance of **1a** at ∼340 nm is 50 times higher in the presence of HsCRY1 (inset), indicating the uptake of **1a** into HsCRY1. (C) The configuration of **1a** bound to HsCRY1 can be reversibly photoisomerized from the *trans*-form to the *cis*-form and back with UV and blue light, respectively. (D) FTIR difference spectrum of the light-induced isomerization of **1a**:HsCRY1 shows pronounced signals in the amide I region and amide II region as highlighted in green and blue, respectively, which are characteristic for changes in the secondary structure. For **1a**:HsCRY1, 12,288 scans were averaged from 12 replicates. Sample-to-sample variation in signal is shown in Figure S5.

Next, full-length HsCRY1 was recombinantly expressed in *E. coli* and purified as confirmed by analysis via Western blot and SDS-PAGE (Figure S3). However, protein yields were consistently low, with ∼0.75 mg of purified HsCRY1 obtained from 10 L of bacterial cell culture. The pooled, purified protein was then loaded with **1a** by incubating overnight. Despite its poor water solubility, an accumulation of **1a** was observed in the presence of HsCRY1 by UV–vis spectroscopy, confirming that **1a** binds to HsCRY1 forming **1a**:HsCRY1 (Figure 2B). Binding of **1a** to the FAD binding site of HsCRY1 was previously demonstrated by thermal shift assays in combination with computational docking simulations.^22^ We found that ∼90% of HsCRY1 was occupied with **1a** based on the experimentally determined extinction coefficient at 360 nm of **1a** in DMSO and a theoretical extinction coefficient at 280 nm of HsCRY1. We estimate the uncertainty in the occupancy of **1a** to be ±10%, using the difference between the extinction coefficients of FAD at 450 nm in insect cryptochrome^13^ and water^40^ as a proxy for the change in the extinction coefficient arising from the dielectric environment of HsCRY1.

Illumination of **1a**:HsCRY1 led to a reversible isomerization, which resembled the isomerization in DMSO but with a minor bathochromic shift of 2 nm to 337 nm in the UV–vis difference spectra (Figure 2C). To obtain insight into the protein response, we recorded FTIR difference spectra of **1a**:HsCRY1 after illuminating the sample with UV or blue light. The resulting difference spectra represent *trans*-to-*cis* and *cis*-to-*trans* isomerization of **1a**:HsCRY1 and are nearly symmetric, demonstrating the reversibility in conformational changes of both, **1a** and the protein moiety of HsCRY1 (Figure S4A). Accordingly, both difference spectra were averaged to obtain a difference spectrum of **1a**:HsCRY1, in which negative and positive signals reflect the inactive and active state, respectively, of protein as well as **1a** (Figure 2D). This FTIR difference spectrum reveals prominent signals in the amide I region (1700–1615 cm^–1^) and the amide II region (1570–1520 cm^–1^) which are characteristic for changes in the protein secondary structure.^41^

Compound **1a** contains an amide moiety impeding an exclusive assignment of signals in the amide I region to HsCRY1. Therefore, we synthesized **1b** with a ^13^C-,^18^O-labeled amide moiety to differentiate between signals of **1** and HsCRY1. Isotope-labeling in **1b** resulted in a downshift of the C=O vibrations by ∼60 cm^–1^ from 1683/1667 cm^–1^ to 1626/1613 cm^–1^ in the FTIR difference spectrum compared to **1a** in DMSO in agreement with quantum chemical calculations (Figures 3A and S2). A similar downshift was observed in the FTIR difference spectrum of **1b**:HsCRY1 compared to that of **1a**:HsCRY1, but the majority of the signals in the amide I region remained unchanged (Figures 3B; S6 and S7). Accordingly, signals originating from **1a** or **1b** are small in intensity compared to those of the HsCRY1 response.

**Figure 3:**
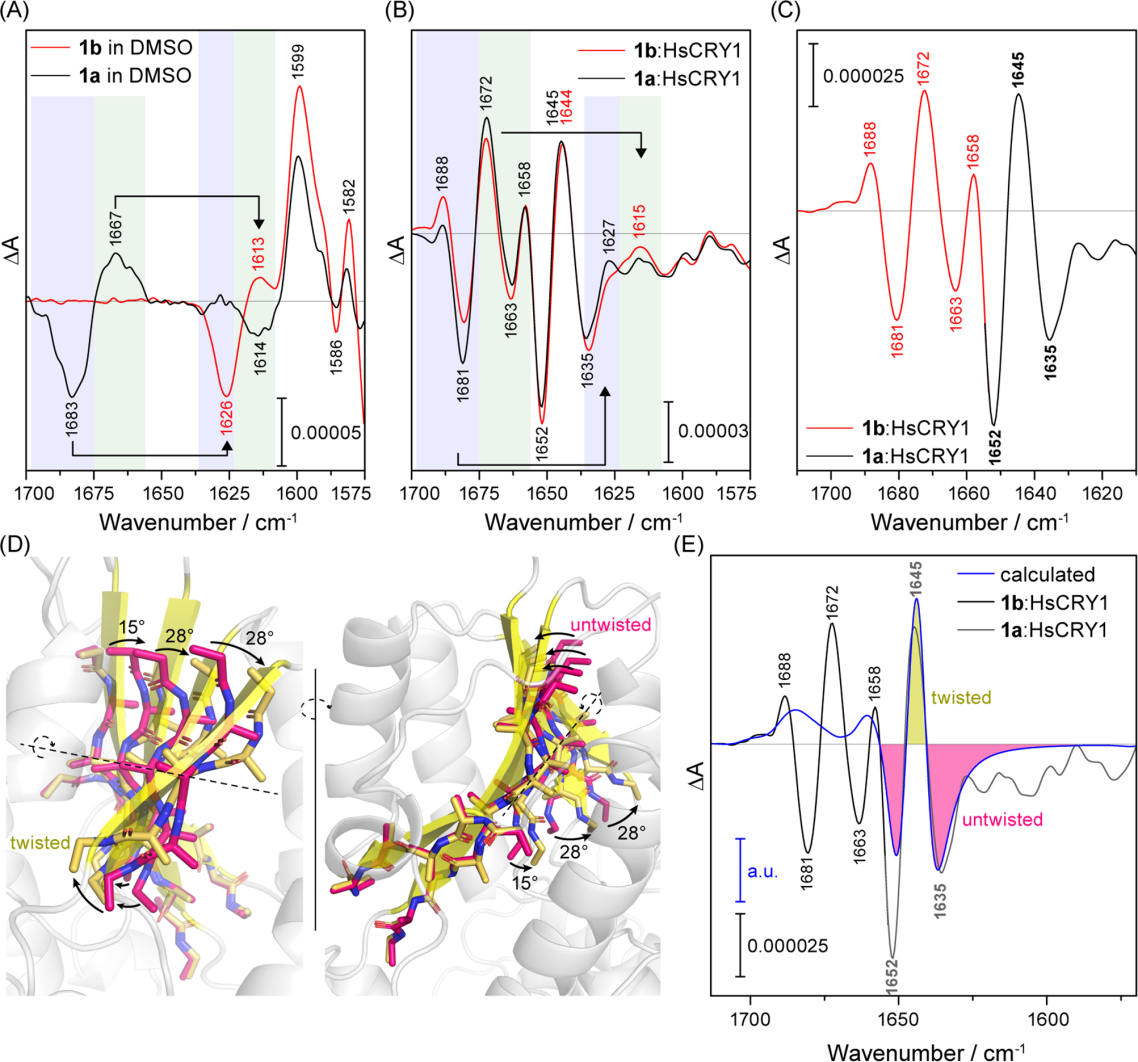
Comparison of infrared difference spectra of photoisomerization of **1a** and isotope-labeled **1b** as well as DFT calculations of the β-sheet in HsCRY1. (A) In DMSO, the ^13^C- and ^18^O-isotopes of the amide group of **1b** shift the C=O stretch to lower wavenumbers as highlighted in blue and green and indicated by arrows. For the spectra of **1a** and **1b** in DMSO, 10,240 and 4096 scans were averaged from 10 and 4 replicates, respectively. (B) A similar shift of the signals is observed as small contributions to the difference spectra of **1a**:HsCRY1 and **1b**:HsCRY1. Spectra of **1a**:HsCRY1 and **1b**:HsCRY1 were normalized to the signal at 1386 (−) cm^–1^ of HsCRY1. For **1b**:HsCRY1, 10,240 scans were averaged from 10 replicates. Sample-to-sample variations are shown in Figure S5. (C) By combining the difference spectra of **1a**:HsCRY1 and **1b**:HsCRY1, a difference spectrum is generated that shows exclusively signals from the protein moiety. (D) Overlay of the polyalanine β-sheet structures used for DFT calculations with the AlphaFold3 model of HsCRY1. Coordinates for the twisted structure were taken from the AlphaFold3 model, while those for the untwisted structure were generated by rotation around a Cα-axis as indicated by the dashed line. (E) Difference signals of HsCRY1 at 1652 (−), 1645 (+) and 1635 (−) cm^–1^ are in good agreement with the calculated twisting motion.

Signals from **1a**/**1b** in the difference spectra of **1a**/**1b**:HsCRY1 completely overlap with signals from HsCRY1, but can be isolated by calculating a double difference spectrum of **1a**:HsCRY1 *minus* **1b**:HsCRY1 (Figure S6). The resulting double difference spectrum closely resembles that of **1a** *minus* **1b** in DMSO. Density functional theory (DFT) calculations on **1a** and **1b** assign signals at 1682 (−) and 1671 (+) cm^–1^ to the C=O stretching vibration of **1a** and signals at 1599 (+) and 1533 (−) cm^–1^ to a combination of the N=N stretching vibration of the azobenzene with δ­(C–H) vibrations of phenyls and the δ­(N–H) vibration of the amide moiety, respectively (Table S3). Differences in band intensities to those in DMSO as well as the absence of a signal at 1599 cm^–1^ (Figure S6) are likely attributable to the restricted environment of **1a** in the FAD binding pocket of HsCRY1.

As the C=O vibrations of **1a** appear between 1700 and 1655 cm^–1^ and those of **1b** between 1640 and 1605 cm^–1^, a combination of the spectra of **1a**:HsCRY1 and **1b**:HsCRY1 results in a difference spectrum of the amide I region that shows exclusively signals originating from HsCRY1 (Figure 3C). The signals of HsCRY1 in the amide I region at 1681 (−) and 1672 (+) cm^–1^ are tentatively assigned to changes in turn elements, signals at 1663 (−) and 1658 (+) cm^–1^ to α-helical elements and signals at 1645 (+) and 1635 (−) cm^–1^ to β-sheets (Table S2).^29,41^ The band at 1688 (+) cm^–1^ might originate from Gln289, which forms a hydrogen bond to the amide moiety of **1a** as deduced from the crystal structure of mouse cryptochrome-1 with TH129 (Figure S8).^22^ DFT calculations on the isolated β-sheet of HsCRY1 suggest that the signals at 1645 (+) and 1635 (−) cm^–1^, as well as partially at 1652 (−) cm^–1^, originate from a structural rearrangement of the β-sheet involving a twisting motion around an axis of Cα-atoms in the β-strands (Figures 3D,E and S9). According to our calculations, the proposed twisting does not lead to a completely planar structure of the β-sheet (Figure S9D). The results are in accordance to theory predicting that the reduced interstrand coupling of C=O vibrations for twisted β-sheets results in higher frequencies compared to planar β-sheets.^42^

A similar signal from rearrangement of the only β-sheet in the PHR was reported already for the phylogenetically distant plant cryptochromes^24,43^ but not yet for the human ortholog HsCRY1 nor any other animal cryptochrome. Moreover, the β-sheet rearrangement in plant cryptochromes was not induced by photopharmacology but by photoreduction of bound FAD_ox_ to the FAD neutral radical (FADH^•^).

### FAD Photoreduction Induces Conformational Response in HsCRY1

Motivated by the substantial structural reorganization of activated HsCRY1 by isomerization of **1a**, we next aimed to investigate whether a photoreaction of FAD bound to HsCRY1 (FAD:HsCRY1) induces structural changes in the protein, particularly given that **1a** binds to the FAD binding site. The light response of HsCRY1 was probed after adding FAD_ox_ to the purified protein. As FAD_ox_ binds to HsCRY1 dynamically,^13^ the protein was loaded with FAD_ox_ by mixing the two components under exclusion of light. Illumination of the sample with blue light for 1–2 s in the absence of external reductants led to broad positive difference absorbance bands in the UV–vis spectrum at above 500 nm, indicating the formation of FADH^•^ as demonstrated previously for human cryptochromes (Figure 4A).^13^ The lifetime of FADH^•^ bound to HsCRY1 was obtained by measuring its absorption at 630 nm after 2 s of blue light illumination (Figure 4B). Fitting the traces with a single-exponential decay yielded τ = 81 ± 10 s at 10 °C under aerobic conditions.

**Figure 4:**
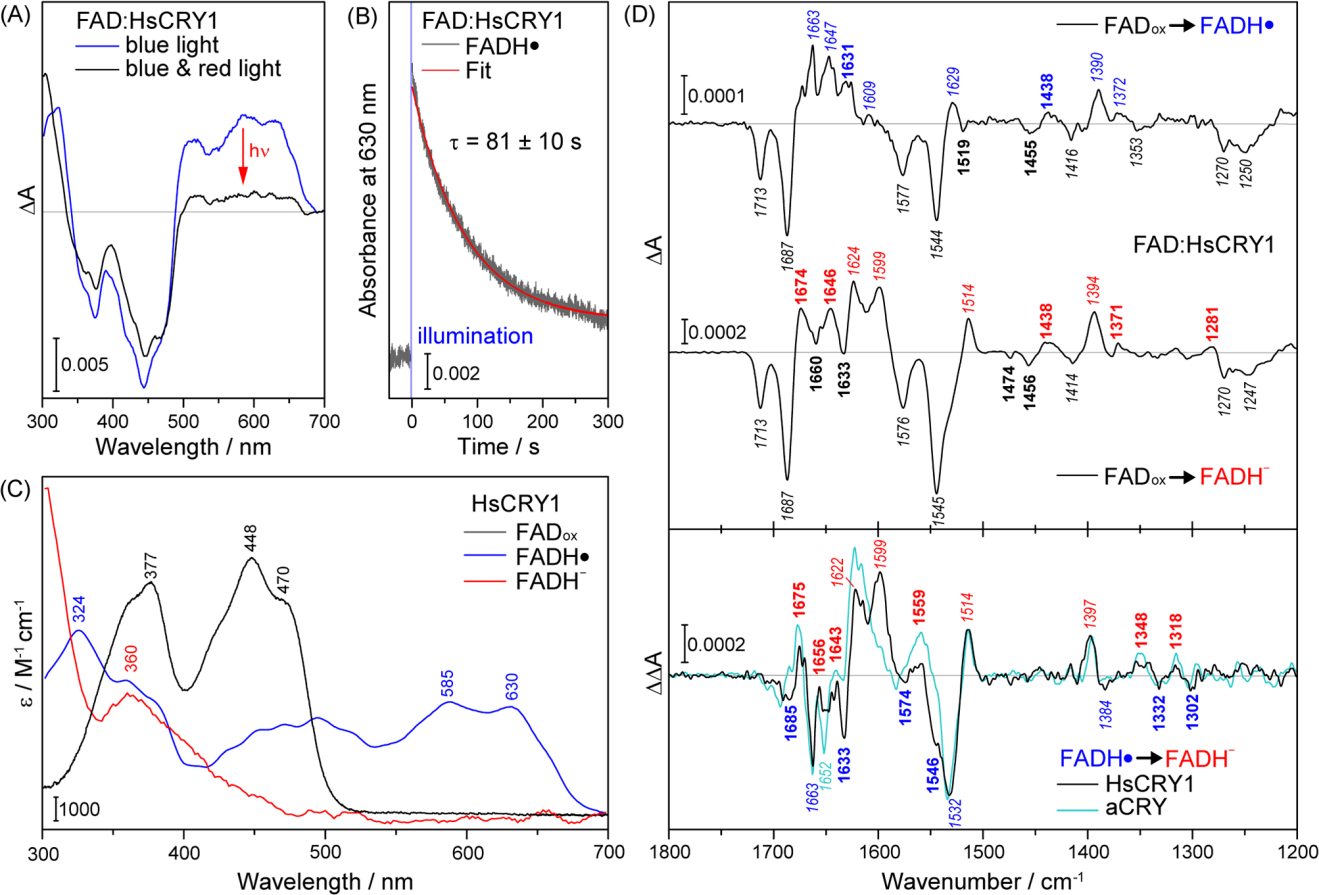
Photoreduction of FAD in HsCRY1 probed by UV–vis and infrared spectroscopy. (A) Blue light illumination at 453 nm produces characteristic spectral features of FADH^•^ in the UV–vis difference spectrum (blue). Subsequent red light illumination at 633 nm leads to reduction to FADH^–^ (black). (B) The decay of FADH^•^ in HsCRY1 was monitored at 630 nm and 10 °C after 2 s of illumination at 453 nm. Data were fitted with a single-exponential function yielding a lifetime of τ = 81 ± 10 s. (C) The extracted UV–vis absorbance spectra for the redox states of FAD_ox_, FADH^•^ and FADH^–^ in HsCRY1 normalized to their extinction coefficients are shown. (D) FTIR difference spectra of the formation of FADH^•^ (top) and FADH^–^ (middle) from FAD_ox_ in HsCRY1 were normalized to the band at 1713 (−) cm^–1^. The double difference spectrum (bottom) of the reduction of FADH^•^ to FADH^–^ in HsCRY1 was obtained from the other two spectra by subtraction. For comparison, the difference spectrum of algal animal-like cryptochrome (aCRY, turquoise)^45^ was superimposed, normalized to the 1514 (+) cm^–1^ marker band of FADH^–^. Signals assigned to the protein moiety and to FAD are indicated in bold and italics, respectively. For the spectrum of FADH^•^ *minus* FAD_ox_, 47,616 scans were averaged from 11 replicates. For the spectrum of FADH^–^ *minus* FAD_ox_, 1536 scans were averaged from 3 replicates. Sample-to-sample variations are shown in Figure S5. The spectrum of aCRY was reproduced from 45. Copyright 2014 American Chemical Society.

Consecutive illumination with red light was performed to selectively excite FADH^•^, which led to a conversion of FADH^•^ to the fully reduced FAD (FADH^–^). Absolute spectra were extracted by using the difference spectra and an absorption spectrum of FAD_ox_ in HsCRY1 (Figure 4C). These spectra confirm the red light-induced species to be FADH^–^. To our knowledge, FADH^–^ is observed here for the first time in a mammalian cryptochrome. The formation of FADH^–^ is typical for proteins of the photolyase/cryptochrome family carrying an asparagine next to the N5 atom of FAD, like Asn393 in HsCRY1 (Figure S8).^44^

The structural response of HsCRY1 to formation of both, FADH^•^ and FADH^–^, was analyzed by FTIR difference spectroscopy with positive signals representing the illuminated state and negative signals the dark state (Figure 4D, top). In the light-induced difference spectrum, most of the signals are assigned to the photoreaction of FAD, like the C=O stretching vibrations that shift from 1713 (−) and 1687 (−) cm^–1^ to 1663 (+) and 1647 (+) cm^–1^ upon FAD_ox_ to FADH^•^ conversion.^45^ This observation indicates that no major structural response of HsCRY1 to the formation of FADH^•^ takes place (Table S3). Only the signal at 1631 (+) cm^–1^ and the difference signal at 1455 (−)/1438 (+) cm^–1^ might arise from the protein moiety of HsCRY1. The light-induced difference spectrum of the conversion from FAD_ox_ to FADH^–^ (Figure 4D, middle) shows characteristic spectral features of FADH^–^ formation at 1624 (+), 1514 (+) and 1394 (+) cm^–1^.^45^ Signals between 1680 cm^–1^ and 1630 cm^–1^ can be assigned to the protein response of HsCRY1, as the C=O stretching vibrations of FADH^–^ appear at lower wavenumbers.^45,46^ Signals at 1674 (+), 1660 (−), 1646 (+) and 1633 (−) cm^–1^ in the amide I region demonstrate that HsCRY1 responds by changes in secondary structure to the formation of FADH^–^.

To isolate signals of the photoreduction of FADH^•^ to FADH^–^ responsible for the protein response, the difference spectrum of FADH^•^ *minus* FAD_ox_ was subtracted from that of FADH^–^ *minus* FAD_ox_ (Figure 4D, bottom). In the resulting double difference spectrum, conversion of FADH^•^ to FADH^–^ is evidenced by the signals at 1663 (−) and 1532 (−) cm^–1^. Most of the FAD signals can be assigned by similarity to the spectrum of animal-like cryptochrome (aCRY) from the green alga *Chlamydomonas reinhardtii*, phylogenetically separated from HsCRY1 (Figure 4D, Table S3).^45^ However, there are two key differences for FAD between the two spectra. First, a C=O stretch of FADH^–^ in HsCRY1 shows a downshifted signal at 1599 (+) cm^–1^ similar to FADH^–^ in halogenase enzymes and aqueous solution,^47^ which points to a more polar binding pocket of FADH^–^ in HsCRY1 than in aCRY. Second, the C=O stretch signal of FADH^•^ in FAD:HsCRY1 is very weak as compared to aCRY at 1652 (−) cm^–1^ pointing to a compensation of this signal by the protein response induced by FADH^–^ formation. Of note, signals from the protein moiety at 1633 (−) and 1546 (−) cm^–1^ are only present in the spectrum of HsCRY1, indicating distinct protein responses to light of FAD:HsCRY1 and aCRY (Figure 4D, bottom). Taken together, we uncovered a unique activation mechanism of HsCRY1, which differs from those of aCRY and plant cryptochromes.

### Similar Conformational Changes in HsCRY1 Induced by FAD Photoreduction and 1a Isomerization

In search of similarities in the responses of HsCRY1 to FAD photoreduction and **1a** isomerization, we compared FTIR difference spectra with respect to signals in the amide I region (Figure 5, Tables S2 and S4). Both difference spectra share several characteristic signals from secondary structure associated with cryptochrome activation as detected at 1672/1674 (+) cm^–1^ and 1660/1663 (−) cm^–1^ for turn structures as well as at 1646/1645 (+) cm^–1^ and 1633/1635 (−) cm^–1^ for β-sheet rearrangement (Figure 5A).^24,43,45^ Signals at above 1680 cm^–1^ and below 1630 cm^–1^ were excluded in the comparison because strong bands from FAD_ox_ and FADH^–^ dominate these spectral regions of FAD photoreduction.

**Figure 5:**
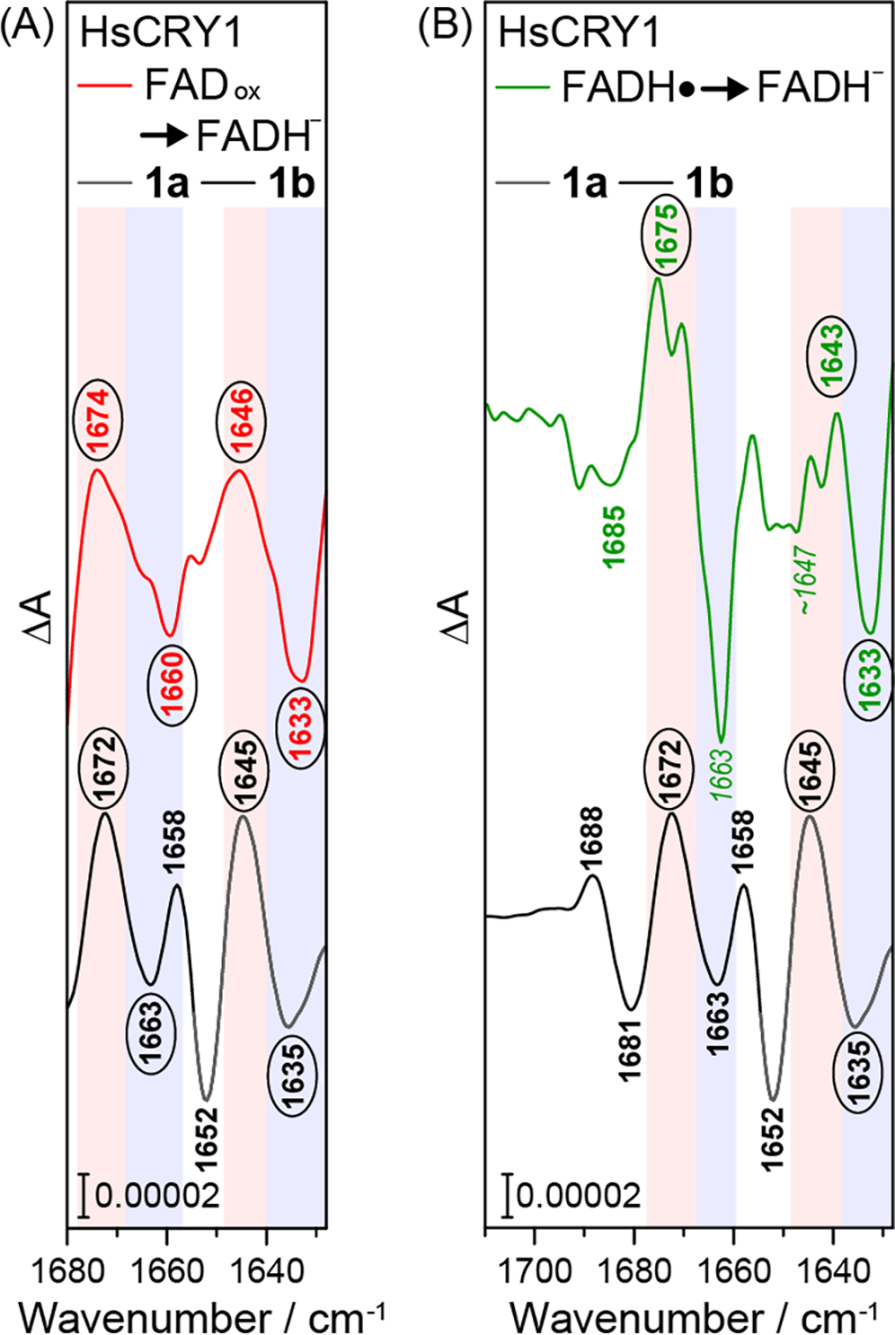
Comparison of the conformational changes induced in HsCRY1 by photoreduction of FAD with those resulting from isomerization of **1a**. Photoreduction of FAD_ox_ to FADH^–^ (A) and FADH^•^ to FADH^–^ (B) in HsCRY1 causes a highly similar pattern (highlighted in blue and red) of protein responses in the FTIR difference spectrum as the photoinduced isomerization of **1a**:HsCRY1 with bands at 1672 (+), 1663 (−), 1645 (+) and 1635 (−) cm^–1^. Signals that are superimposed by contributions from FADH^•^ carbonyls are indicated in italics. Spectra of FAD_ox_ to FADH^–^ and FADH^•^ to FADH^–^ were normalized to the β-sheet signal at 1646 (+)/1633 (−) cm^–1^ and the turn signal at 1674 (+) cm^–1^of **1a**:HsCRY1, respectively.

The region at above 1680 cm^–1^ is additionally accessible when we compare the conversion from FADH^•^ to FADH^–^ to the activation of **1a**:HsCRY1 (Figure 5B). Here, signals attributed to β-sheet rearrangement and turn structures are present at 1633 (+) cm^–1^ and 1675 (−) cm^–1^ as well. However, the C4=O stretch of FADH^•^ results in a stronger negative signal at 1663 (−) cm^–1^ than for **1a**:HsCRY1. Moreover, the negative C2=O stretch of FADH^•^ at ∼1647 cm^–1^ in HsCRY1 (1652 cm^–1^ for aCRY) compensates the strong positive β-sheet signal found in the **1a**:HsCRY1 spectrum at 1645 cm^–1^.

Differences between the photoreduction- and isomerization-induced responses of HsCRY1 are apparent in the region 1700–1680 cm^–1^ and at 1652 (−) cm^–1^, where a prominent signal is absent for FAD:HsCRY1. It should be noted that identical structural responses are unlikely as the redox active unit of FAD, the isoalloxazine ring, and the isomerizing azobenzene group of **1a** are separated within the FAD binding site when both structural models are overlaid (Figure 1A).^22^ Nevertheless, both comparisons reveal almost identical responses that involve characteristic turn-structural changes and β-sheet rearrangements, indicating a similar outcome of light-induced and pharmacologic activation of HsCRY1 (Figure 5). The conformational changes in HsCRY1 due to *cis*-**1a** lengthens the circadian period in mammalian cells.^22^ Supported by the comparison of the HsCRY1 response to FADH^–^ formation with the response of plant as well as animal-like cryptochromes, our results suggest that the formation of FADH^–^ in HsCRY1 has a similar structural impact on this clock protein, which may potentially lead to physiological effects such as a lengthened circadian period.

## Discussion

What are the implications from photopharmacology for the mechanism of HsCRY1 activation and a possible light-dependent role? HsCRY1 is a central component of the circadian clock and therefore a suitable target for pharmacological manipulation. Understanding the structural impact of a circadian clock modulator on HsCRY1 aids in the development of new modulators and may reveal insights into the binding and allostery of interaction partners of cryptochrome-1 in humans. Photopharmacological compounds that are selective for cryptochromes allow not only for spatiotemporal modulation of the mammalian circadian rhythm,^22^ but also open up the opportunity to study the structural response of cryptochrome in solution by time-resolved spectroscopic techniques such as infrared difference spectroscopy.

Here, we studied full-length HsCRY1 by photochemically isomerizing the bound compound **1a** to its pharmacologically active *cis*-isomer. Structural studies on **1a** with cryptochrome have so far been limited to the PHR domain of the homologous mouse cryptochrome-1.^22^ By exploiting selective isotope labeling, we were able to distinguish infrared signals of **1a** from those of the protein moiety. Photoisomerization of **1a** in HsCRY1 was fully reversible as evident in the UV–vis (Figure 2C) and FTIR difference spectra (Figure S4), which includes structural changes in **1a** and the protein moiety. In contrast to the local changes identified in the PHR of mouse cryptochrome-1 with TH129 by protein crystallography,^22^ we observed distinct signals that are consistent with changes in secondary structure including α-helices, turn elements as well as a β-sheet (Figure 2C, Table S2). This difference might be explained by restrictions in protein flexibility by crystal packing and by the absence of the CCE in the crystals. Of particular note are the signals at 1645 (+) and 1635 (−) cm^–1^, which are consistent with a twisting motion of the only β-sheet of HsCRY1 according to DFT calculations (Figures 1A and 4D). Accordingly, local changes in the conformation of amino acids induced by *cis*-**1a** as a structural mimic of TH129 propagate from the FAD binding pocket to the β-sheet over a distance of ∼25 Å. The sequence of events in HsCRY1 activation and the response of the CCE to the modulator have yet to be resolved.

The similarity in changes of the β-sheet structure induced by *trans*-to-*cis* isomerization of **1a**:HsCRY1 and photoreduction of FAD_ox_ in plant cryptochromes motivated us to investigate the response of HsCRY1 to FAD photoreduction despite the reported low binding affinity of FAD_ox_.^13^ The formation of FADH^–^ but not of FADH^•^ induced conformational changes in HsCRY1 similar to those which were observed for isomerization of **1a** (Figure 4). These similarities may indicate that *cis*-**1a** generates a structural response of HsCRY1 that mimics a physiological response of HsCRY1 to the light-induced formation of FADH^–^ (Figure 5). In addition, signal progression to the β-sheet upon activation might be a conserved pathway among cryptochromes.

Melanopsin mediates ocular light sensing for circadian photoentrainment and pupil constriction^16^ and has also been suggested to contribute to spatial vision.^48^ However, recent evidence suggests that extraocular light responses are present in mammals with roles of the opsins OPN3, OPN4, and OPN5 currently under investigation.^49^ Similarly, it has been speculated that HsCRY1 may possess light-dependent functions in light-exposed tissues, in addition to its light-independent role as a key component of the circadian clock.^50,51^ We demonstrated by FTIR difference spectroscopy that HsCRY1 responds to the formation of FADH^–^ by changes in secondary structure. The resulting signal patterns in the amide I region of HsCRY1 resemble but are not identical to those of the photoreceptors pCRY and aCRY,^24,43,45^ indicative for a light-dependent role of HsCRY1. In plant,^52,53^ insect^54^ and animal-like^55^ cryptochromes, photoreduction leads to the dissociation of the CCE from the PHR. Analogously, in HsCRY1, the CCE binds to the PHR in competition with CLOCK/BMAL1 as well as PER2 and thereby regulates their affinity to the PHR.^56^ Mutation-driven dissociation of the CCE leads to a lengthening of the circadian rhythm, as observed in patients with familial delayed sleep phase disorder.^56,57^ A light-induced release of the CCE in HsCRY1 remains to be demonstrated to complement these findings.

In contrast to other cryptochromes, mammalian cryptochromes were shown to only transiently bind FAD_ox_ with moderate affinities in the micromolar range.^13,15^ The moderate affinities are unique to animal type II cryptochromes such as HsCRY1, which have a disordered phosphate binding loop and a displaced protrusion motif compared to the evolutionarily related insect cryptochromes and (6–4) photolyases.^13,23^ The resulting reduced interaction with the phosphates and the adenine moiety of FAD causes increased solvent exposure of the FAD cavity, which allows for FAD and other binding partners to diffuse in and out. However, the flavin binding region has been reported to be conserved in animal type II cryptochromes,^13^ supporting FAD photochemistry in HsCRY1. We found FADH^•^ to be stable for minutes (τ = 81 s) in the presence of HsCRY1. If FADH^•^ dissociated during this time period, the radical would directly disproportionate to FAD_ox_ and FADH^–^ in the millisecond time regime, even at this low concentration.^58^ Additionally, the observed red light-induced formation of FADH^–^ in the absence of any external reducing agent further demonstrates that FADH^•^ is bound to the protein. A higher binding affinity of FADH^•^ than of FAD_ox_ may be rationalized by an additional hydrogen bond formed between FADH^•^ and conserved N393 as observed in cyclobutane pyrimidine dimer (CPD) photolyases and *Drosophila*, *Arabidopsis*, *Synechocystis*, human (DASH)-type cryptochrome (CRY-DASH).^25,59^ Extrapolation from the 10 °C measurements to body temperature by assuming Arrhenius behavior yields FADH^•^ lifetimes of about 30 s. This transient population may only allow for an accumulation of FADH^•^ and subsequent formation of FADH^–^ in HsCRY1 under high light conditions, leading to much higher cofactor occupancies than in the dark (Figure 6).

**Figure 6:**
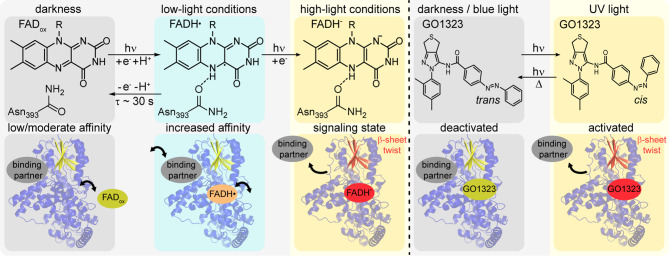
Model of a postulated light-dependent role of HsCRY1 in human physiology and the effect of GO1323 (**1a)** on HsCRY1. In the dark, the binding pocket of HsCRY1 is devoid of FAD and available for other interaction partners such as FBXL3 or the circadian clock modulators TH129/GO1323. Under low light conditions, FADH^•^ accumulates in HsCRY1 and has a lifetime of τ ∼30 s at body temperature. FADH^•^ competes with FBXL3 or TH129/GO1323 for binding. Under high light conditions, conversion of FADH^•^ to FADH^–^ triggers a conformational change of HsCRY1 involving a rearrangement of the β-sheet, which might be transduced to binding partners. Unlike FAD_ox_, GO1323 is already bound to HsCRY1 in the dark and therefore competes with FAD_ox_ and FBXL3 for binding. Isomerization from *trans*- to *cis*-form causes a conformational change in HsCRY1 involving a shift in the lid loop and rearrangement of the β-sheet, which induces a lengthening of the circadian rhythm via binding partners.

Such putative mechanism requires the sequential absorption of two photons for activation as a slow and insensitive response to light, thereby preventing disruptions of the circadian rhythm by transient or low-intensity external light cues. To support our model, we numerically simulated the population of FADH^•^ at the photostationary state formed from FAD_ox_ in HsCRY1 at full sunlight (model AM1.5 g) with a photon flux density of 1 mmol m^–2^ s^–1^ (Figure S10A). Assuming a quantum yield of 2%, this estimate yields a conversion of ∼5% to FADH^•^ that could be available for production of FADH^–^. Significantly higher conversions were simulated when the focusing effect of the eye’s lens was taken into account (Figure S10B). To date, there is no experimental evidence for a light-induced activation of mammalian cryptochromes *in vivo*. However, an integrating high light effect has been observed in human keratinocytes, where blue light promotes the protein expression of HsCRY1 as light exposure increases.^51^ Furthermore, full-length cryptochrome-1 has been demonstrated to be expressed in the retina of humans and great apes but only in the outer segment of shortwave-sensitive 1 (SWS1) cones.^50^ For these cryptochromes, a function in the TTFL has been dismissed, which indicates that HsCRY1 may have an additional nonclock function.^50^

HsCRY1 is expressed in all tissues as a key regulator of the circadian clocks. In light-inaccessible tissues, strong FAD_ox_ binding may offer no functional advantage, would deplete cellular FAD pools and block interactions with partners targeting the FAD binding site.^23^ Therefore, the moderate binding affinity of HsCRY1 for FAD_ox_ may reflect its proposed additional role as a high-light receptor in light-accessible regions. HsCRY1 may act as a bifunctional protein in addition to its interaction with natural binding partners and modulation by (photo-) pharmacological compounds.

## Conclusions

A potential photosensory role of mammalian cryptochromes has been debated for decades and remains unresolved, because their involvement in circadian clock photoentrainment and other ocular responses has been excluded. Here, we demonstrate that the human circadian clock protein cryptochrome-1 undergoes conformational changes upon light-induced formation of fully reduced FAD that resemble those induced by photopharmacological modulation. The requirement to form fully reduced flavin may enable the protein to accumulate flavin as neutral radical in the cell overcoming the low affinity to oxidized flavin. At the same time, a much higher light intensity is required for the photoconversion than for the conversion of oxidized flavin to flavin neutral radical as it takes place, for example, in plant cryptochromes. Together, these findings motivate us to propose a model in which human cryptochrome-1 is a bifunctional protein, acting both as a core circadian clock component and as a high-light receptor. As the light-induced structural changes observed in HsCRY1 in the presence of FAD do not provide direct evidence for a function of HsCRY1 as light receptor, our model needs to be validated by biochemical and cellular assays.

The availability of an isomer-specific photoactivatable modulator allowed us to establish a procedure to probe the responses of HsCRY1 by FTIR difference spectroscopy and to reveal conformational changes which may cause changes in interaction to the binding partners and the observed shifts in the phase of the clock. There are complementary biophysical techniques available, particularly time-resolved approaches, which require a photoactivation. We envision that our results now motivate and enable other studies to elucidate both, responses to GO1323 photoisomerization and to flavin photochemistry, and thereby further increase our molecular understanding of mammalian cryptochromes.

## Supplementary Material



## Notes

Reversibility of switching of **1a**, *cis*-to-*trans* ratios of compound **1a** (Table S1), changes in the *cis*-to-*trans* ratios by photoisomerization (Figure S1), FTIR difference spectra of isomerization of **1a** and **1b** in DMSO and calculated IR spectra (Figure S2), analysis of isolated HsCRY1 by Western blot and SDS-PAGE (Figure S3), FTIR difference spectra of isomerization of **1a** and**1b** in HsCRY1 (Figure S4), sample-to-sample variation of FTIR difference spectra (Figure S5), double difference spectra of **1b** *minus* **1a** in DMSO and in HsCRY1 (Figure S6), assignment of FTIR difference signals of **1a**:HsCRY1 (Table S2), assignment of FTIR difference signals of **1a** (Table S3), comparison of light-induced FTIR difference spectra of **1a:**HsCRY and **1b:**HsCRY1 (Figure S7), binding sites of FAD and TH129 in mammalian cryptochrome-1 (Figure S8), DFT calculations of IR spectra of the β-sheet of HsCRY1 (Figure S9), assignment of signals from FAD in the FTIR difference spectra of HsCRY1 (Table S4), simulation of the population of FADH^•^ at the photostationary state in HsCRY1 (Figure S10), experimental procedures, MS spectra, NMR spectra (PDF)

## Abbreviations

aCRY: animal-like cryptochrome
BMAL1: brain and muscle ARNT-like 1
CCE: C-terminal extension
CLOCK: circadian locomotor output cycles kaput
CPD: cyclobutane pyrimidine dimer
CRY: cryptochrome
CRY-DASH: *Drosophila*, *Arabidopsis*, *Synechocystis*, human -type cryptochrome
dCRY: *Drosophila* cryptochrome
DFT: density functional theory
FAD: flavine adenine dinucleotide
FADH^•^: FAD neutral radical
FADH^–^: fully reduced FAD
FAD_ox_: oxidized FAD
FBXL3: ubiquitin ligase F-box/LRR-repeat protein 3
FTIR: Fourier transform infrared
HsCRY1: human cryptochrome-1
PER: period protein
PHR: photolyase homology region
SCN: suprachiasmatic nucleus
TTFL: transcription-translation feedback loop.
