# Supplementary material for: The Human Circadian Clock Protein Cryptochrome‑1 Responds to Photochemistry by Conformational Changes
Source: J Am Chem Soc. 2026 Jul 31;148(31):33217–28. doi: 10.1021/jacs.6c06484 (PMC13474925; doi:10.1021/jacs.6c06484)
Supplement: Supplementary file 1 [file ja6c06484_si_001.pdf]

Supporting Information

The Human Circadian Clock Protein Cryptochrome-1 Responds to Photochemistry by Conformational Changes

1. Supporting Figures and Tables

2. Experimental Procedures, MS Spectra & NMR Spectra

Axel Jeibmann[1,2§], Mian Qi[3§], Marten Hellmich[2], Adelheid Godt[3], Tilman Kottke[1,2*], Lukas Goett-Zink[1,2*]

[1]Biophysical Chemistry and Diagnostics, Medical School OWL, Bielefeld University, Universitätsstraße 25, 33615 Bielefeld, Germany;
[2]Biophysical Chemistry and Diagnostics, Faculty of Chemistry, Bielefeld University, Universitätsstraße 25, 33615 Bielefeld, Germany;
[3]Organic and Macromolecular Chemistry, Faculty of Chemistry, Bielefeld University, Universitätstraße 25, 33615 Bielefeld, Germany

[*]For correspondence: Tilman Kottke, tilman.kottke@uni-bielefeld.de; Lukas Goett-Zink, lukas.goett-zink@uni-bielefeld.de

[§]A.J. and M.Q. contributed equally

Contents 1: Supporting Figures and Tables

Reversibility of the switching of **1a** between *trans*- and *cis*-configuration verified with ^{1}H NMR spectroscopy 3

Table S1. The *cis*-to-*trans* ratios of compound **1a** determined by ^{1}H NMR spectroscopy. 3

Figure S1. Changes in the *cis*-to-*trans* ratios of compound **1a** by photoisomerization as determined by ^{1}H NMR spectroscopy. 4

Figure S2. FTIR difference spectra of the isomerization of **1a** and **1b** in DMSO and calculated IR spectra of **1a** and **1b** in the *cis*- and *trans*-configuration. 5

Figure S3. Analysis of isolated HsCRY1 by SDS-PAGE and Western blot. 6

Figure S4. FTIR difference spectra of light-induced isomerization of **1a** and **1b** in HsCRY1.......7

Figure S5. Sample-to-sample variation of light-induced FTIR difference spectra of **1a**:HsCRY1. 8

Figure S6. Double difference spectra of isomerization of **1b** *minus* **1a** in DMSO and bound to HsCRY1. 9

Figure S7. Comparison of light-induced FTIR difference spectra of **1a** and **1b** in HsCRY1. 9

Table S2. Assignment of FTIR difference signals of **1a**:HsCRY1. 10

Table S3. Assignment of FTIR difference signals of **1a**. 11

Figure S8. Binding sites of FAD and modulator TH129 in mammalian cryptochrome-1. 12

Figure S9. DFT calculations of IR spectra of the parallel β-sheet of HsCRY1 in different twisted conformations. 13

Table S4. Assignment of signals from FAD_{ox}, FADH• and $FADH^-$ in the FTIR difference spectra of HsCRY1 and numbering of the isoalloxazine ring of FAD. 14

Figure S10. Estimated conversion of FAD_{ox} to FADH• in HsCRY1 in dependence of continuous light intensity up to full sunlight. 15

References for Supporting Figures and Tables..16

Reversibility of the switching of **1a** between *trans*- and *cis*-configuration verified with ^{1}H NMR spectroscopy

A mixture of compound **1a** (3.6 mg) and DMSO-d_6 (600 µL) was briefly heated. This gave a homogeneous solution, which remained a solution throughout the whole experiment.

The solution was alternately illuminated with UV light with a wavelength of 385 nm and with blue light with a wavelength of 415 nm. The illumination was performed right next to the NMR spectrometer. An ^{1}H NMR spectrum was recorded before the first illumination, immediately after each of the five consecutive illuminations, and 120 s after the last illumination with UV light. For quantitative analysis, the intensity of the ^{1}H NMR signal of DMSO served as a reference. The ratios of the combined signal intensities of the *cis*- and *trans*-isomers of compound **1a** relative to the DMSO signal remained constant in the seven spectra. A comparison of the spectra reveals that the intensities of the signals corresponding to the *cis*-isomer increase by UV light exposure and decrease by exposure to blue light at 415 nm concomitant with a quantitatively corresponding opposite change of the signal intensities of the *trans*-isomer. This correspondence is very obvious through the signals of the methyl groups, as shown in Figure S1. The ratios of the two isomers are listed in Table S1. The correlation between the signal intensities of the two isomers testifies the reversibility of the photoinduced switching from *trans*- to *cis*-configuration and back for compound **1a**. In the absence of light, no interconversion between the two isomers happened within 120 s as evidenced by the last NMR spectrum.

Table S1. The *cis*-to-*trans* ratios of compound **1a** determined by ^{1}H NMR spectroscopy. The listed treatments were applied to the same sample consecutively. NMR spectra were recorded immediately after each treatment.

^{1}H NMR spectrum	Treatment	*cis* : *trans*
1	**1a** dissolved in DMSO-d_6 without any illumination	2 : 98
2	illumination with UV light at 385 nm for 120 s	15 : 85
3	illumination with blue light at 415 nm for 120 s	12 : 88
4	illumination with UV light at 385 nm for 120 s	21 : 79
5	illumination with blue light at 415 nm for 120 s	12 : 88
6	illumination with UV light at 385 nm for 120 s	21 : 79
7	kept in the dark for 120 s	21 : 79

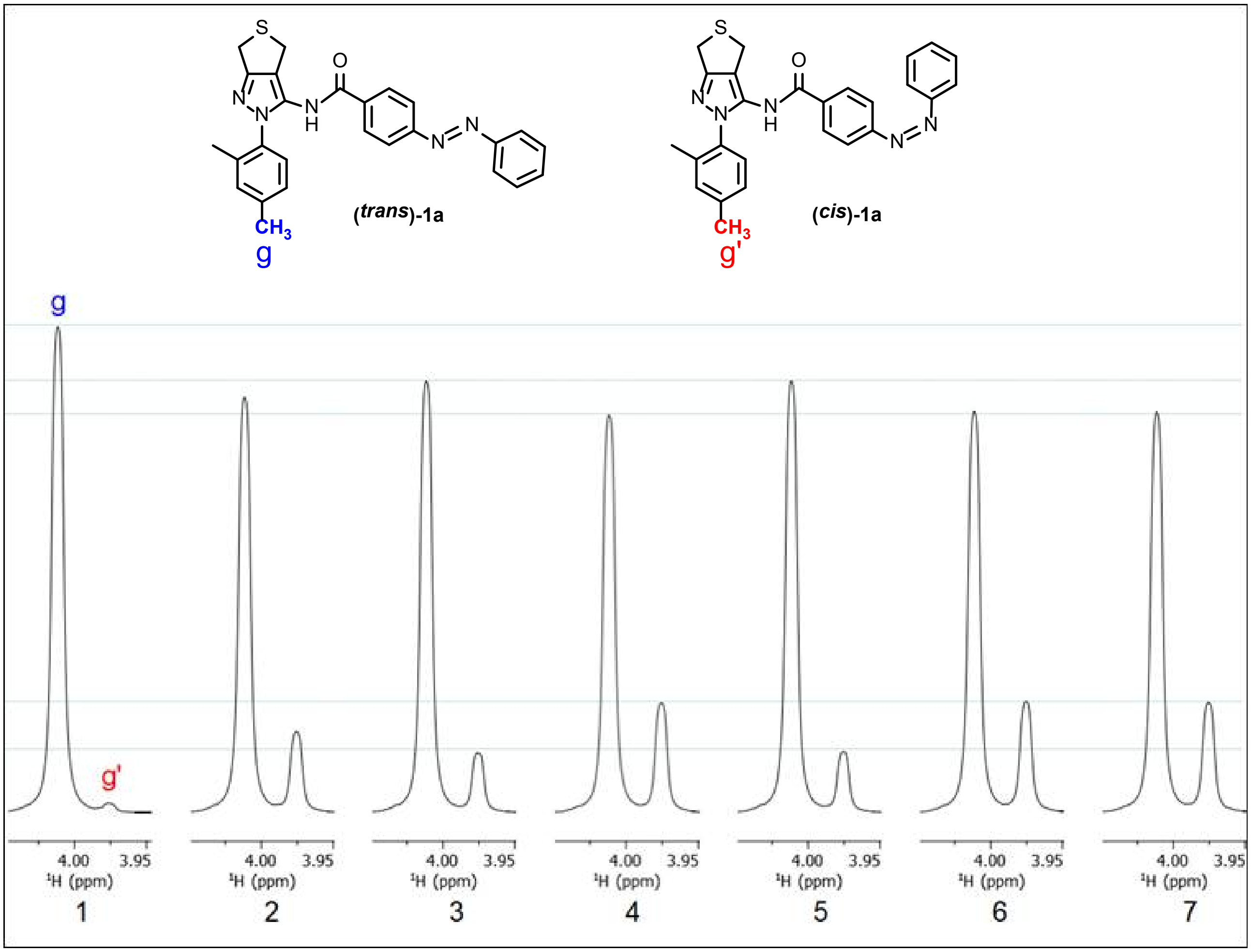

Figure S1. Changes in the *cis*-to-*trans* ratios of compound **1a** by photoisomerization as determined by ^{1}H NMR spectroscopy. Cutouts of the ^{1}H NMR spectra 1-7 (500 MHz) for the signals of methyl groups are shown. Quantitative evaluation is presented in Table S1. The spectra are normalized to the signal of DMSO at 2.50 ppm.

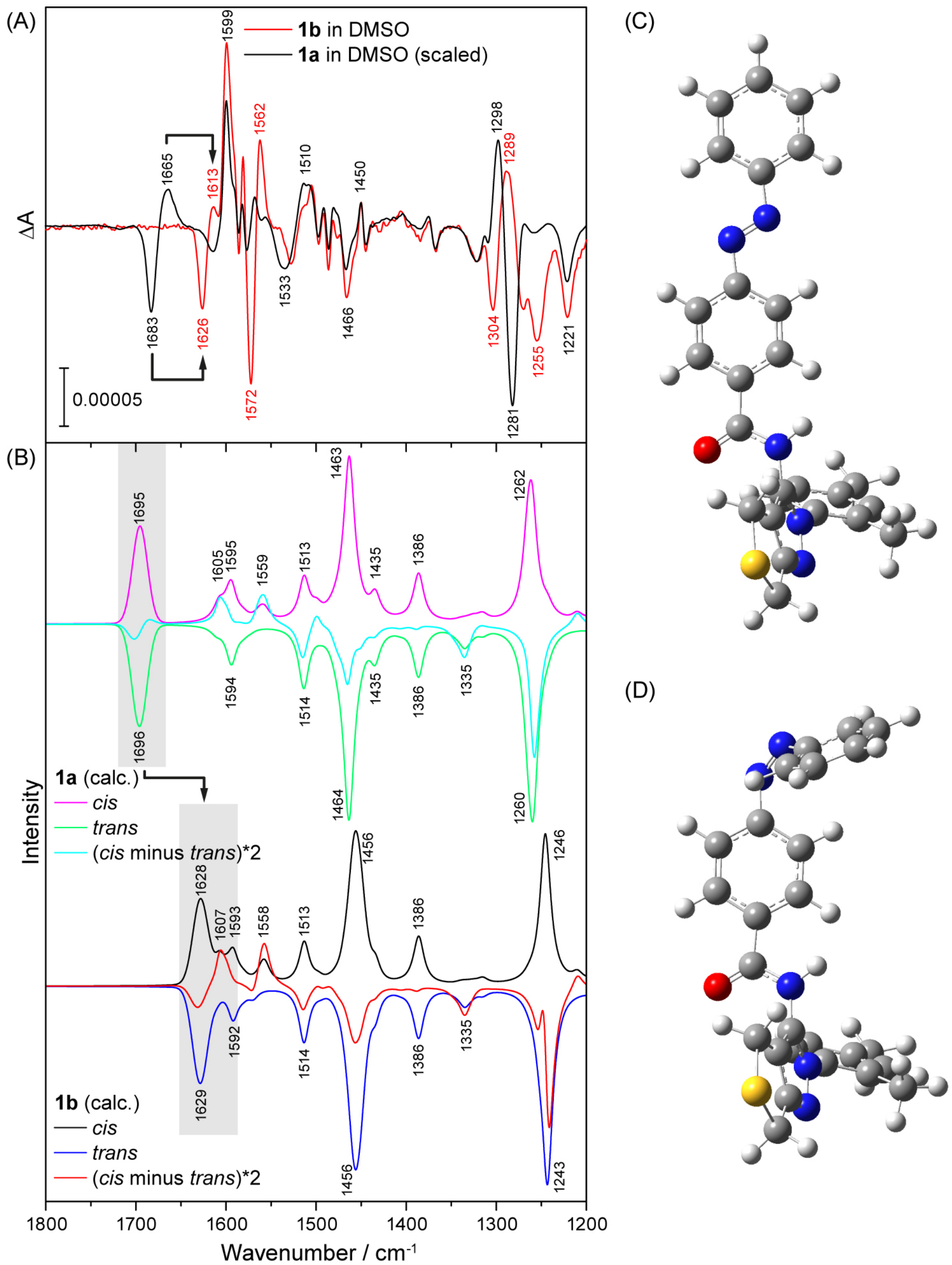

Figure S2. FTIR difference spectra of the isomerization of **1a** and **1b** in DMSO and calculated IR spectra of **1a** and **1b** in the *cis*- and *trans*-configuration. (A) Light-induced FTIR difference spectra of **1a** and **1b** in DMSO show that ^{13}C- and ^{18}O-isotope labeling of the amide of **1a** results in a shift of the CO mode by ~57 cm^{-1} in **1b**. (B) This shift is in accordance with DFT calculations of the normal modes of the *trans*- and *cis*-isomers of **1a** and **1b**. Structures after geometry optimization are shown for the *trans*- (C) and *cis*- (D) isomers.

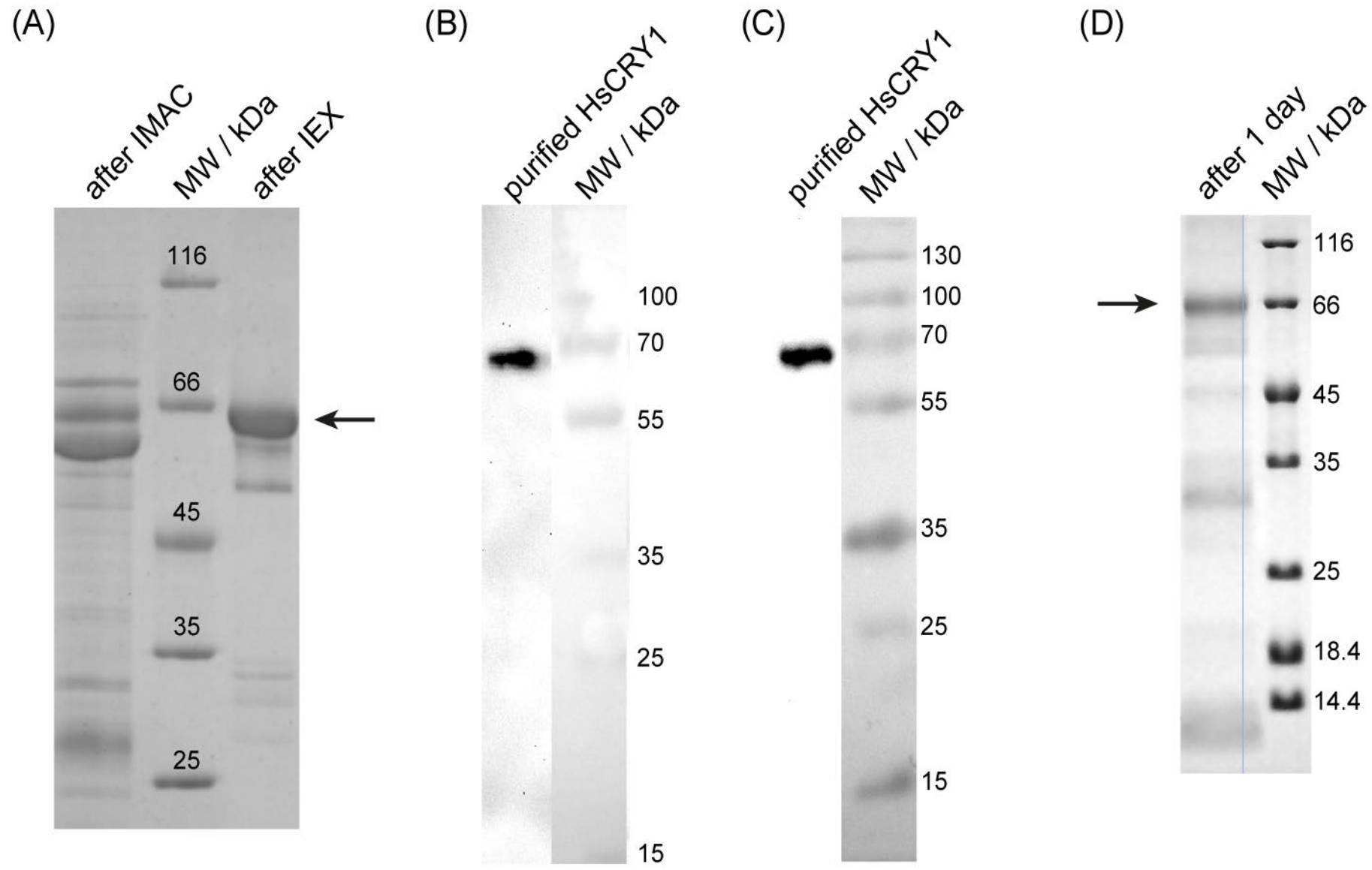

Figure S3. Analysis of isolated HsCRY1 by SDS-PAGE and Western blot. (A) SDS-PAGE analysis after the IMAC purification reveals major contaminations of HsCRY1 that become minor after a second purification step with IEX. An asterisk denotes the band position corresponding to HsCRY1 at about 66 kDa according to the molecular weight standard (MW). In the analysis by Western blot, full-length HsCRY1 was detected close to the 70 kDa band of the molecular weight standard (MW) by both, the Anti-His antibody directed against the N-terminal His_6-tag (B) and the Strep-Tactin horseradish peroxidase conjugate binding the C-terminal Strep-tag of the protein (C). (D) SDS-PAGE analysis shows that HsCRY1 is stable after incubation for 24 hours at 4°C.

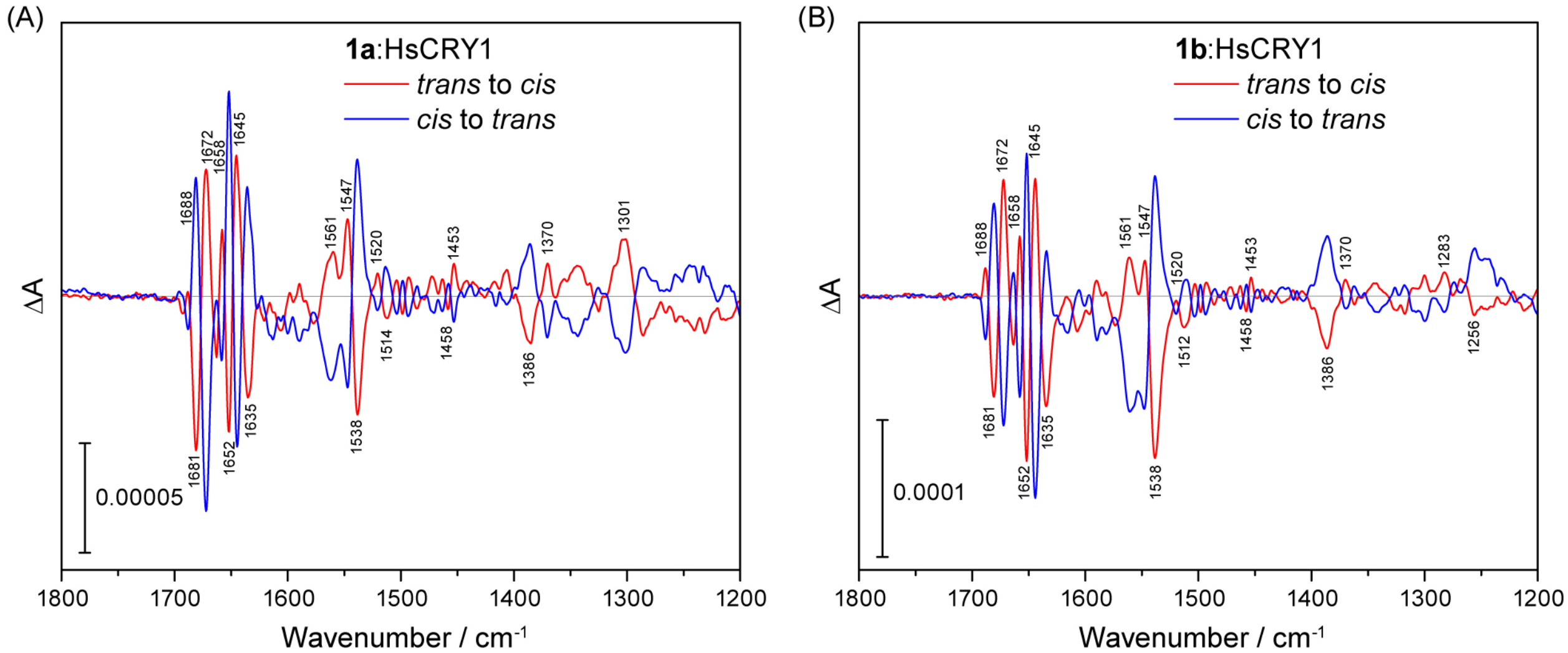

Figure S4. FTIR difference spectra of light-induced isomerization of **1a** and **1b** in HsCRY1. Difference spectra of **1a** and **1b** bound to HsCRY1 are nearly symmetrical, demonstrating the reversibility of the isomerization of both photo-pharmacological compounds in HsCRY1 as well as of the protein response.

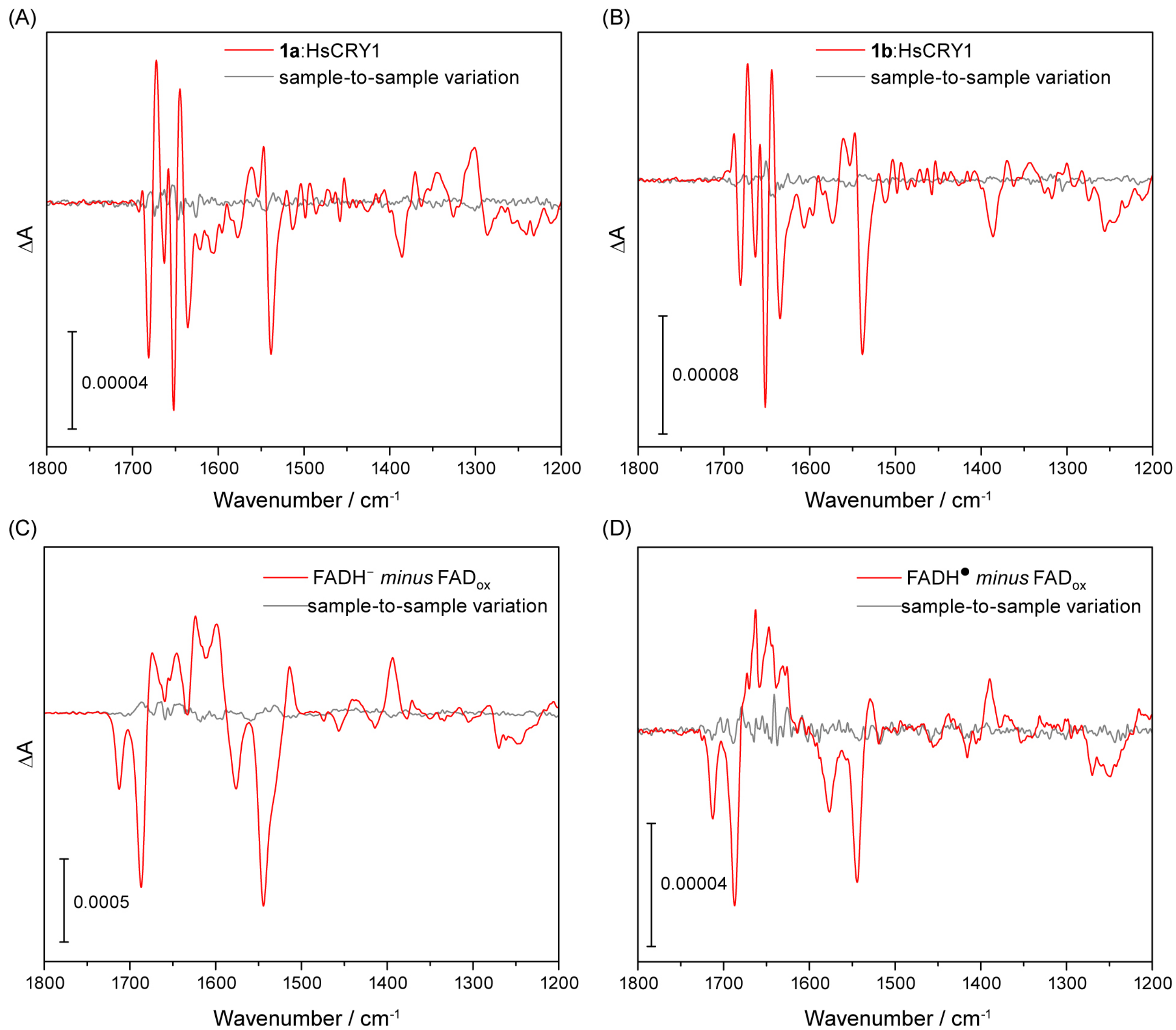

Figure S5. Sample-to-sample variation of light-induced FTIR difference spectra of **1a**:HsCRY1 (A), **1b**:HsCRY1 (B) and FAD:HsCRY1 for the conversion of FAD_{ox} to $FADH^-$ (C) and FAD_{ox} to FADH• (D).

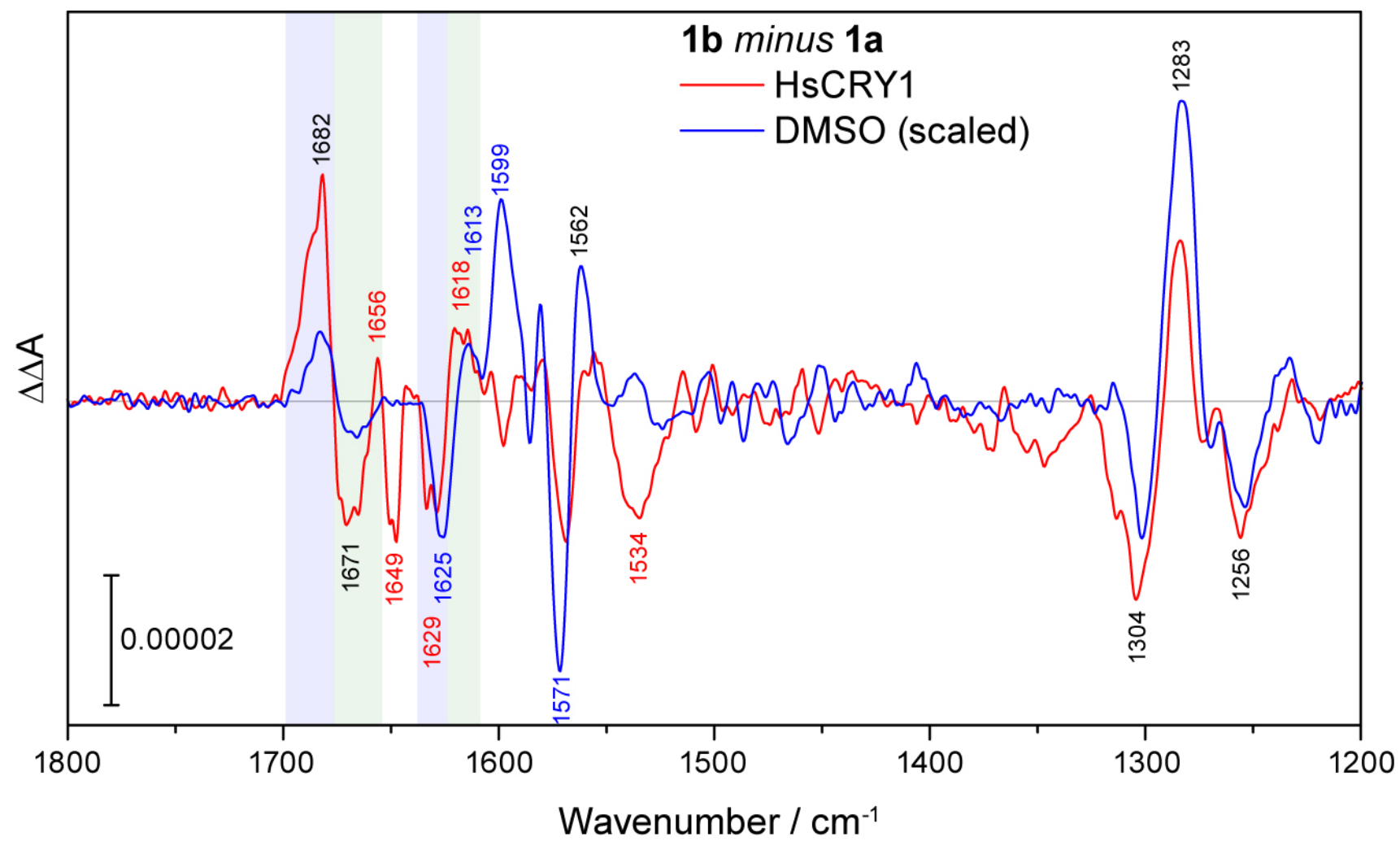

Figure S6. Double difference spectra of isomerization of **1b** *minus* **1a** in DMSO and bound to HsCRY1. Isotope labeling in **1b** results in a similar downshift of the signals of the carbonyl moiety in DMSO and bound to HsCRY1, respectively, as highlighted in blue and green. Slight changes between the band positions in the double difference spectra likely result from the different environment of the modulators in DMSO and in the binding pocket of HsCRY1.

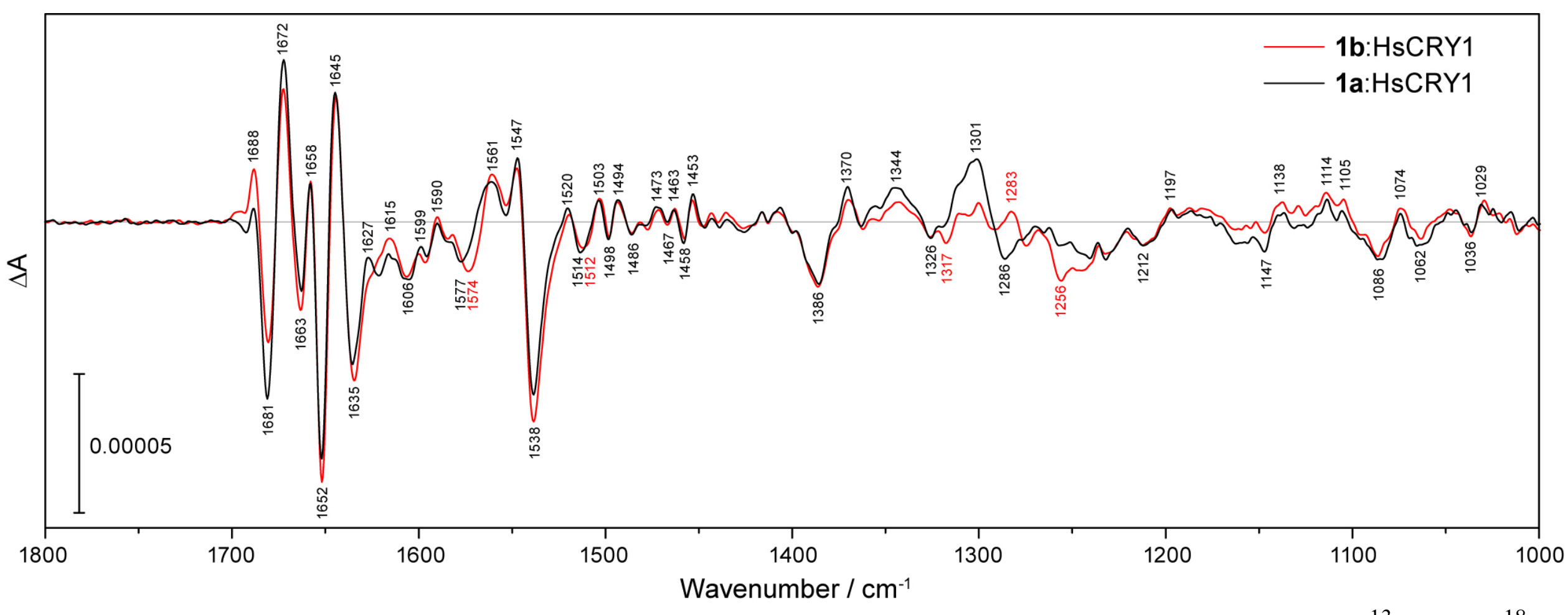

Figure S7. Comparison of light-induced FTIR difference spectra of **1a** and **1b** in HsCRY1. ^{13}C- and ^{18}O-isotope-labeling of **1b** leads to small shifts in wavenumbers in the amide I and amide II regions and to distinct changes in the region between 1326-1212 cm^{-1} in the difference spectrum of **1b** as compared to that of the non-labeled compound **1a**.

Table S2. Assignment of FTIR difference signals of **1a**:HsCRY1.

	**Active *cis*-conformer, positive bands / cm^{-1}**	**Inactive *trans*-conformer, negative bands / cm^{-1}**	**Assignment**
Amide I	1688		HsCRY1 (Gln289)[a]
		1681	HsCRY1 (turns, Gln289, β-sheet)[a], *trans*-**1a** (ν(C=O))[b,c]
	1672		HsCRY1 (turns, β-sheet)[a], *cis*-**1a** (ν(C=O))[b,c]
		1663	HsCRY1 (turns)[a]
	1658		HsCRY1 (α-helix)[a]
		1652	HsCRY1 (minor contributions of β-sheet and α-helix)[a,c]
	1645		HsCRY1 (β-sheet)[a,c]
		1635	HsCRY1 (β-sheet)[a,c]
	1615		HsCRY1 (Gln)[a], minor contributions of **1a**[b,c]
		1606	HsCRY1 (Gln)[a], minor contributions of **1a**[b,c]
	1590		Not assigned
Amide II		1578	Not assigned
	1561		HsCRY1 (amide II)[a], minor contribution of **1a**[b]
	1547		HsCRY1 (amide II)[a]
		1538	HsCRY1 (amide II)[a]
	1520		HsCRY1 (amide II)[a]
		1514	HsCRY1 (amide II, Tyr, Trp)[a], minor contributions of **1a**[b]
	1503		HsCRY1 (Trp, Tyr)[a], minor contributions of **1a**[b]
		1498	HsCRY1 (Trp, Tyr)[a]
	1494		HsCRY1 (Phe CC ring)[a]
		1458	HsCRY1 (side chains, CH_3)[a]
	1453		HsCRY1 (side chains, CH_3)[a]
Amide III		1386	HsCRY1 (amide III)[a]
	1370		HsCRY1 (amide III)[a], minor contributions of GO1323[b]
	1344		HsCRY1 (amide III, Trp, δCH)[a], GO1323[b]
	1300-1030	1300-1030	HsCRY1 (side chains)[a], GO1323[b]

[a]Barth and Zscherp[1]; [b]assigned by isotope labeling in this work; [c]assigned by DFT calculations in this work

Table S3. Assignment of FTIR difference signals of **1a**.

**Active *cis*-conformer, positive bands / cm^{-1}**	**Inactive *trans*-conformer, negative bands / cm^{-1}**	**Assignment**
	1682	ν(C=O)[a,b]
1671		ν(C=O)[a,b]
1599		ν(N=N), δ(C-H) ring 1 and ring 2[a,b]
	1533	ν(N=N), δ(N-H), δ(C-H) ring 1 and ring 2, δ(C-H) ring 3[a,b]
1510		δ(N-H), δ(C-H) ring 1 and ring 2, δ(C-H) ring 3[a,b]
	1466	δ(N-H)[a,b]
1450		δ(N-H)[a,b]
1298		δ(N-H), ν(C-C, ring 1 and ring 2, ν(C-C) ring 3[a,b]
	1281	δ(N-H), ν(C-C) ring 1 and ring 2, ν(C-C) ring 3[a,b]

[a]assigned by isotope labeling in this work; [b]assigned by DFT calculations in this work

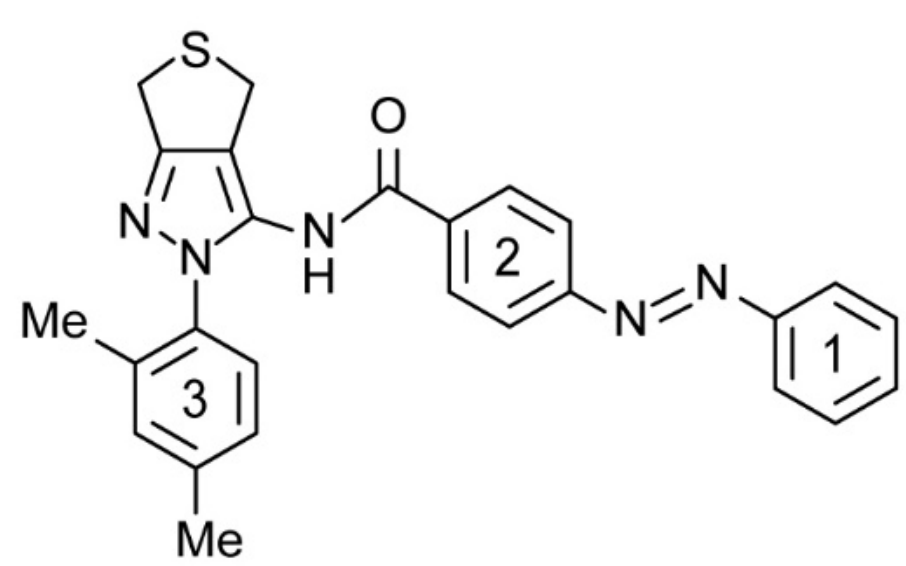

1a

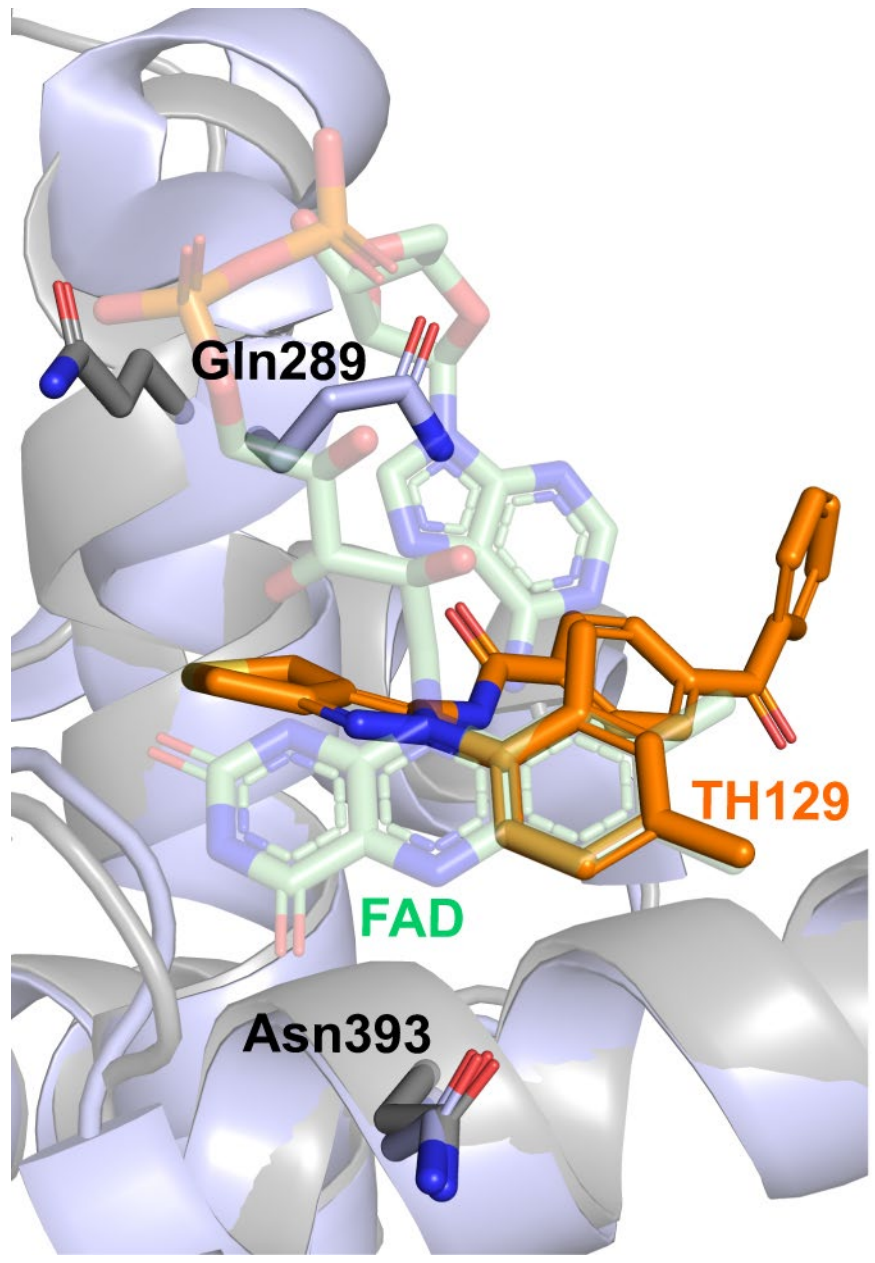

Figure S8. Binding sites of FAD and modulator TH129 in mammalian cryptochrome-1. Overlay of the AlphaFold3 model of FAD-bound HsCRY1 (gray) and the crystal structure of TH129-bound mouse cryptochrome-1 (PDB code: 7D19, light blue).[2] Modulator TH129 (orange) binds to the binding pocket of the cofactor FAD (green).

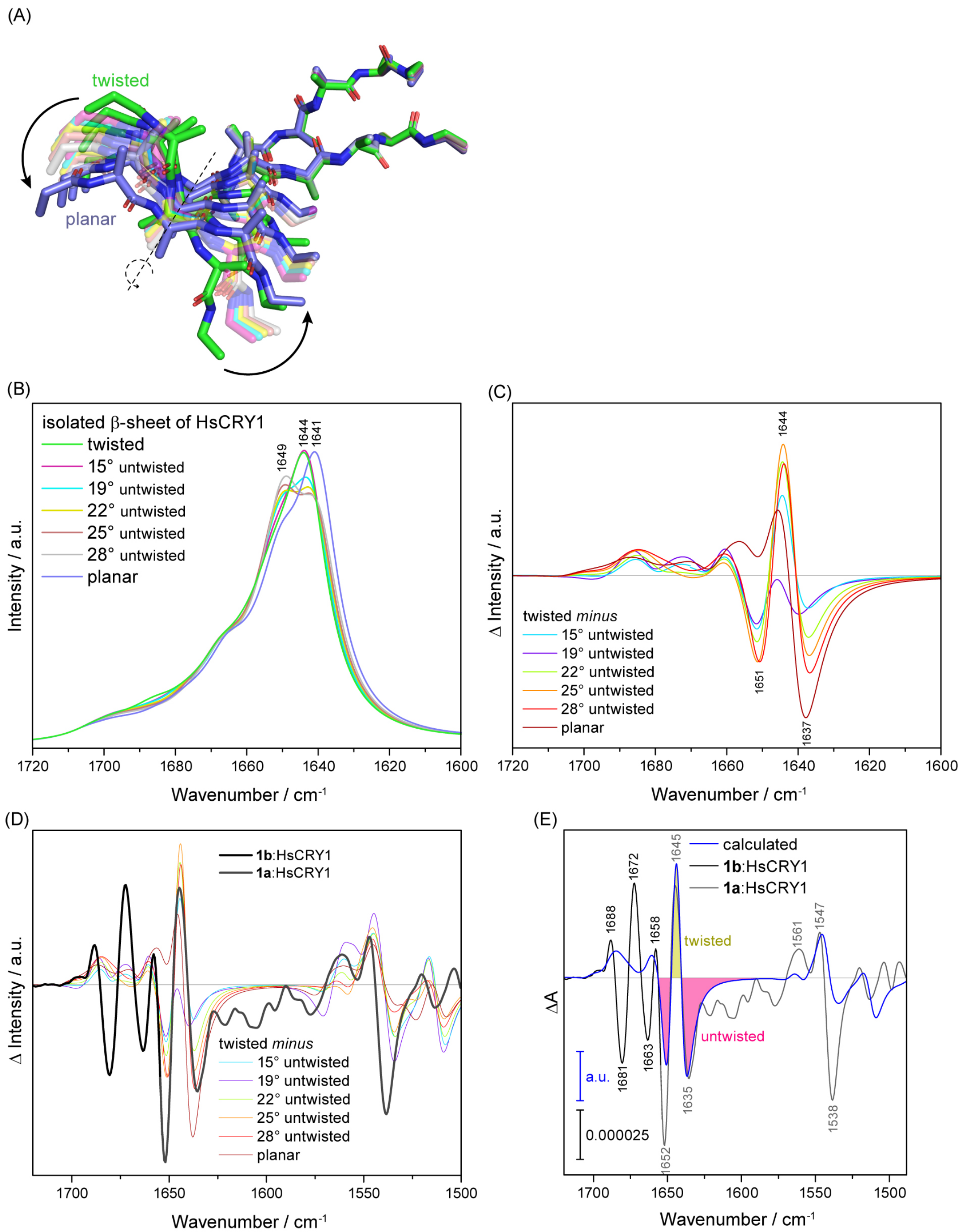

Figure S9. DFT calculations of IR spectra of the parallel β-sheet of HsCRY1 in different twisted conformations. (A) Coordinates for the twisted structure were taken from the AlphaFold3 model, while those for the untwisted structure were generated by rotation around an axis of Cα-atoms, as indicated by the dotted line. For better visualization, hydrogen atoms are not included in the illustration. (B) Calculated IR spectra of the isolated β-sheet of HsCRY1 in different twisted conformations. (C) A twist of the β-sheet results in a loss of intensity at 1644 cm^{-1} and an increase at 1651 and 1637 cm^{-1} in the difference spectrum. (D) A twist between 15° and 28° was analyzed. Difference spectra were calculated with reference to the spectrum of the original structure and compared to the experimental difference spectra of **1a**:HsCRY1 and **1b**:HsCRY1. (E) A twist of ~28° best describes the experimental difference spectra of **1a**:HsCRY1 and **1b**:HsCRY1.

Table S4. Assignment of signals from FAD_{ox}, FADH• and $FADH^{-}$ in the FTIR difference spectra of HsCRY1 and numbering of the isoalloxazine ring of FAD.

	**FAD_{ox} negative bands / cm^{-1}**	**FADH• positive and negative bands / cm^{-1}**	**$FADH^{-}$ positive bands / cm^{-1}**	**Assignment**
	1713			ν(C4=O)[3-7]
Amide I	1687			ν(C2=O)[3-4, 7]
		1663		ν(C4=O)[3]
		1647		ν(C2=O)[3]
			1621	ν(C=O)[3, 8]
			1599	ν(C=O)[8]
		1609		ν(C4aN5), ν(CC) ring I[3]
Amide II	1577			ν(C4aN5), ν(CN) ring III, ν(CC) ring I[3-4]
	1544			ν(CC) ring I, ν(C10aN10), ν(C10aN1)[3-4]
		1529		ν(C10aN1), ν(CC) ring I[3-4]
			1514	δ(N5H), δ(N1H), δ(C6H), δ(C9H)[3-4, 8]
	1416			ν(C=O), δ(N5H)[3]
			1397	δ(N5H)[3-4, 9]
Amide III		1390		δ($N10CH_3$), ν(C=O), δ(N3H)[3]
		1372		ν(CC) ring I, ν(CN) ring III, δ(N5H)[3]
	1353			δ($N10CH_3$), δ($C8CH_3$), ν(CN) ring III[3]
	1270			ν(CN) ring II, ν(CC) ring I[3]
	1250			ν(C5aN5), δ(CH) ring I, ν(CC) ring I[3]

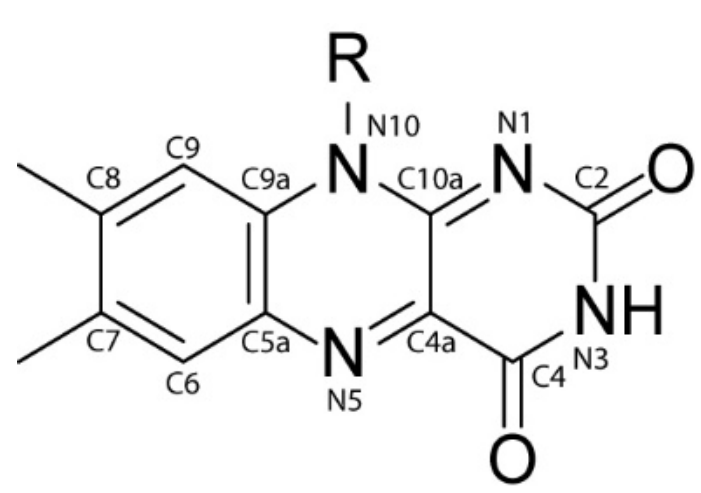

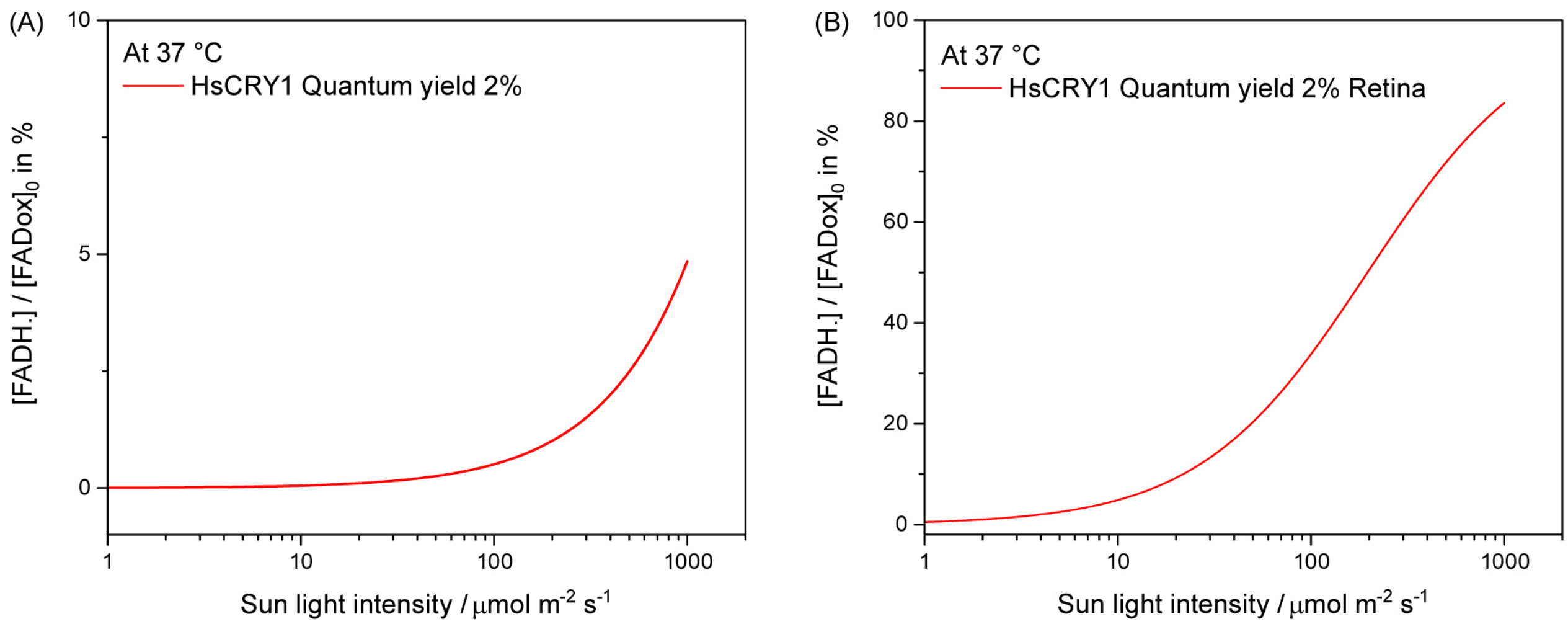

Figure S10. Estimated conversion of FAD_{ox} to FADH• in HsCRY1 in dependence of continuous light intensity up to full sunlight. (A) The proportion of FADH• in the photostationary state that formed from the initial concentration of FAD_{ox} was estimated by numerical simulations[10] using MATLAB (MathWorks). The simulation yielded a maximal conversion of 5% at 37 °C and full sunlight corresponding to a photon flux density of 1 mmol photons m^{-2} s^{-1}. The concentration of FADH• is given by the equation[11]:

$$\left[FADH\bullet\right] = -k \cdot \left[FADH\bullet\right] + \Phi \cdot F \cdot \int \varepsilon(\lambda)\rho(\lambda)d\lambda \cdot \left[FAD_{ox}\right]$$

with the rate constant of thermal recovery k = $(30\ s)^{-1}$ at 37 °C, a quantum yield Φ = 2%, a photon flux F = 1 mmol m^{-2} s^{-1}, the integral extinction coefficient ε(λ) calculated from the spectrum of FAD_{ox} (Figure 4C), and the normalized quantum emission spectrum of the sun ρ (model AM1.5g). The lower limit of the previously reported quantum yields for plant and insect cryptochromes of Φ = 2-18% was used.[12-14] (B) Estimated conversion of FAD_{ox} to FADH• in HsCRY1 under the assumption that incident light is focused onto the human retina, resulting in a 100-fold[15] increase in light intensity. The simulations were performed under the assumption that the light is not attenuated by pigments and that dissociation of FADH• does not take place within its lifetime. Dissociation of FAD_{ox} and excitation of FADH• to $FADH^-$ have not been included in the model.

References for Supporting Figures and Tables

(1) Barth, A.; Zscherp, C., What vibrations tell us about proteins. *Q. Rev. Biophys.* **2002,** *35* (4), 369-430.

(2) Kolarski, D.; Miller, S.; Oshima, T.; Nagai, Y.; Aoki, Y.; Kobauri, P.; Srivastava, A.; Sugiyama, A.; Amaike, K.; Sato, A.; Tama, F.; Szymanski, W.; Feringa, B. L.; Itami, K.; Hirota, T., Photopharmacological Manipulation of Mammalian CRY1 for Regulation of the Circadian Clock. *J. Am. Chem. Soc.* **2021,** *143* (4), 2078-2087.

(3) Spexard, M.; Thöing, C.; Beel, B.; Mittag, M.; Kottke, T., Response of the sensory animal-like cryptochrome aCRY to blue and red light as revealed by infrared difference spectroscopy. *Biochemistry* **2014,** *53* (6), 1041-1050.

(4) Yamada, D.; Zhang, Y.; Iwata, T.; Hitomi, K.; Getzoff, E. D.; Kandori, H., Fourier-transform infrared study of the photoactivation process of *Xenopus* (6-4) photolyase. *Biochemistry* **2012,** *51* (29), 5774-5783.

(5) Spexard, M.; Immeln, D.; Thöing, C.; Kottke, T., Infrared spectrum and absorption coefficient of the cofactor flavin in water. *Vib. Spectrosc.* **2011,** *57* (2), 282-287.

(6) Kottke, T.; Batschauer, A.; Ahmad, M.; Heberle, J., Blue-light-induced changes in *Arabidopsis* cryptochrome 1 probed by FTIR difference spectroscopy. *Biochemistry* **2006,** *45* (8), 2472-2479.

(7) Immeln, D.; Pokorny, R.; Herman, E.; Moldt, J.; Batschauer, A.; Kottke, T., Photoreaction of plant and DASH cryptochromes probed by infrared spectroscopy: The neutral radical state of flavoproteins. *J. Phys. Chem. B* **2010,** *114* (51), 17155-17161.

(8) Wille, G.; Ritter, M.; Friedemann, R.; Mäntele, W.; Hübner, G., Redox-triggered FTIR difference spectra of FAD in aqueous solution and bound to flavoproteins. *Biochemistry* **2003,** *42* (50), 14814-14821.

(9) Zhang, Y.; Iwata, T.; Yamamoto, J.; Hitomi, K.; Iwai, S.; Todo, T.; Getzoff, E. D.; Kandori, H., FTIR study of light-dependent activation and DNA repair processes of (6-4) photolyase. *Biochemistry* **2011,** *50* (18), 3591-3598.

(10) Bannister, S.; Böhm, E.; Zinn, T.; Hellweg, T.; Kottke, T., Arguments for an additional long-lived intermediate in the photocycle of the full-length aureochrome 1c receptor: A time-resolved small-angle X-ray scattering study. *Struct. Dyn.* **2019,** *6* (3), 034701.

(11) Kottke, T.; Heberle, J.; Hehn, D.; Dick, B.; Hegemann, P., Phot-LOV1: photocycle of a blue-light receptor domain from the green alga *Chlamydomonas reinhardtii*. *Biophys. J.* **2003,** *84* (2), 1192-1201.

(12) Schroeder, L.; Oldemeyer, S.; Kottke, T., Time-resolved infrared spectroscopy on plant cryptochrome-relevance of proton transfer and ATP binding for signaling. *J. Phys. Chem. A* **2018,** *122* (1), 140-147.

(13) Müller, P.; Bouly, J. P.; Hitomi, K.; Balland, V.; Getzoff, E. D.; Ritz, T.; Brettel, K., ATP binding turns plant cryptochrome into an efficient natural photoswitch. *Sci. Rep.* **2014,** *4* 5175.

(14) Öztürk, N.; Song, S. H.; Selby, C. P.; Sancar, A., Animal type 1 cryptochromes. Analysis of the redox state of the flavin cofactor by site-directed mutagenesis. *J. Biol. Chem.* **2008,** *283* (6), 3256-3263.

(15) Sliney, D. H., Quantifying retinal irradiance levels in light damage experiments. *Curr. Eye Res.* **1984,** *3* (1), 175-179.

Contents 2: Experimental Procedures, MS Spectra & NMR Spectra

Expression, Purification and Sample Preparation 18
SDS-PAGE and Western Blot Analysis 18
UV-vis Spectroscopy 19
FTIR Spectroscopy 20
Quantum Chemical Calculations 20
$^{16}O/^{18}O$ Exchange of Azobenzenecarboxylic Acid **4** 21
Determination of the Isotopic Enrichments 23
Signals of the *Z*-Isomer of Photopharmacological Compounds **1a** and **1b** in the NMR Spectra 24
Synthesis of Photopharmacological Compounds **1a** and **1b** 27
MS Spectra 34
NMR Spectra 38
References for Experimental Procedures, MS Spectra & NMR Spectra 90

Expression, Purification and Sample Preparation

For recombinant protein expression, the coding sequence of HsCRY1 with an RBS sequence, an N-terminal His_6-tag and a C-terminal Strep-tag II was synthesized and cloned into a pET21(+) vector using BamHI and XhoI restriction sites (pET21(+)-HsCRY1) by Twist Bioscience HQ. HsCRY1 was expressed in *E. coli* SoluBL21 (DE3). The cells were incubated at 37 °C and 120 rpm in DYT medium with 200 µg/mL ampicillin to an OD_{600} of 0.5 and then cooled to 18 °C. At an OD_{600} of 0.8, expression was induced by adding 10 µM isopropyl-β-D-thiogalactopyranoside and the cells were cultivated for 20 h. Cells were harvested by centrifugation (6,000 g, 20 min, 4 °C) and stored for subsequent purification steps at -80 °C. For isolation of HsCRY1, cells were disrupted using a French press (SLM Amincor, 1000 bar, 2 cycles) in 50 mM Tris buffer, pH 7.5, with 150 mM NaCl, DNase I and protease inhibitor (cOmplete EDTA-free protease inhibitor cocktail, Roche). The lysate was centrifuged for 45 min with 108,000 ×g at 4 °C (Avanti J-30 I, Beckman Coulter). Imidazole was added to the supernatant to a final concentration of 10 mM and the sample was loaded on a HisTrap column (EconoFit Nuvia IMAC 5 mL, Bio-Rad) using a fast protein liquid chromatography system (NGC Chromatography System, Bio-Rad, Hercules). After washing with 50 mM Tris buffer, pH 7.5, 300 mM NaCl, 45 mM imidazole, the protein was eluted with 245 mM imidazole. The protein was washed with 50 mM Tris buffer, 150 mM NaCl, pH 7.5, and stored for further purification steps at -80 °C. For purification using anionic exchange chromatography, the protein was loaded on a self-packed Source15Q column (Cytiva) in 50 mM Tris, 60 mM NaCl, pH 9 ($8\ mS\cdot cm^{-1}$) and was eluted by washing with 190 mM NaCl ($20\ mS\cdot cm^{-1}$). After elution, the protein was washed with 50 mM sodium phosphate buffer, 150 mM NaCl, pH 7.5, using a centrifugal filter and then concentrated.

For binding of **1a** or **1b** to HsCRY1, 1.5 µL of 15 mM **1a** or **1b** in DMSO and 3 µL protease inhibitor in DMSO (Halt™ Protease Inhibitor Cocktail, Thermo Fisher Scientific) were incubated with 350 µL of 62 µM HsCRY1 over night at 4 °C. For FTIR difference spectroscopy, the sample was centrifuged at 11,000 ×g and 4 °C, washed with buffer and concentrated to a final concentration of 1.5 mM HsCRY1 with **1a**.

For binding of FAD to HsCRY1, the protein was mixed with FAD_{ox} under exclusion of blue light. Samples for FTIR difference spectroscopy on HsCRY1 with FAD_{ox} were concentrated to 1.5 mM of HsCRY1 with 2 mM of FAD for the short blue light-induced illumination to produce FADH•, and 4 mM HsCRY1 with 1 mM FAD for the photoreduction of FAD_{ox} to $FADH^{-}$.

SDS-PAGE and Western Blot Analysis

Successful purification of full length HsCRY1 was controlled via Western blot against the N-terminal His_6-tag and the C-terminal Strep-tag II. Transfer to a nitrocellulose membrane (0.45 µm, Amersham Protran Premium, Cytiva) occurred at 25 V for 25 min. The N-terminal His_6-tag was addressed by incubating the membrane with primary anti-His antibody (His-Tag (D3I1O) XP® Rabbit mAb, Cell Signaling Technology) and a secondary anti-

rabbit antibody (Peroxidase AffiniPure Goat Anti-Rabbit IgG (H+L), Jackson ImmunoResearch). The C-terminal Strep-tag II was addressed by incubating the membrane with a Strep-Tactin-horseradish peroxidase conjugate (Strep-Tactin® HRP, IBA Lifesciences). Chemiluminescent detection (ECL Chemostar, Intas Science Imaging) was performed by incubating the membrane with reaction solution (LumiLight® Western-Blotting Substrate, Roche). A prestained marker (PageRulerTM Plus, Prestained Protein Ladder, Thermo Fisher Scientific) was used as molecular weight standard. An SDS-PAGE with the same samples, as for the Western-Blot analysis was performed as a control. An unstained protein molecular weight marker (Thermo Scientific) and both samples, normalized to a protein concentration of 2 mg, were applied on a 10 % gel.

UV-vis Spectroscopy

UV-vis spectra were recorded on a Shimadzu UV-2450 spectrometer. Isomerization of 18.8 µM **1a** in DMSO was investigated by illuminating the sample with a UV LED (30 mW·cm^{-2}, Lumileds) at 385 nm for 120 s and with a blue LED (160 mW·cm^{-2}, Lumileds) at 410 s for 30 s. For the spectrum of **1a** in water, 1 µL of 15 mM **1a** in DMSO was added to 999 µL ultrapure water and incubated over night at 4 °C. The sample was centrifuged at 11,000 ×g and 4 °C and a UV-vis spectrum was recorded of the supernatant. Spectra of **1a** in HsCRY1 were recorded by diluting the concentrated FTIR samples (1:66). The occupancy of HsCRY1 with **1a** was determined using the theoretical extinction coefficient of HsCRY1 in water (ε_{280nm} = 128,800 M^{-1}·cm^{-1}) and the experimental extinction coefficients of **1a** in DMSO (ε_{360nm} = 8,793 M^{-1}·cm^{-1}; ε_{280nm} = 7,072 M^{-1}·cm^{-1}). For isomerization, the sample was illuminated with a blue LED (160 mW·cm^{-2}, Lumileds) at 410 nm for 10 s to reach the photostationary state. Spectra were recorded before and after illumination with UV light using a pulsed laser (355 nm, 5 mJ, 10 Hz, Quantel Ultra 100, Lumibird) for 10 s and after illumination with blue light.

UV-vis spectra of FAD-bound HsCRY1 were recorded using samples containing 30 µM HsCRY1 and 12 µM FAD in the absence of external reductants. Absorbance spectra were measured in fast acquisition mode and a sampling interval of 2 nm at 4 °C to slow down dark recovery of the FAD. The sample was illuminated briefly with a blue LED (48 mW·cm^{-2}, Lumileds) for less than 2 s at 453 nm to produce FADH• but minimize formation of $FADH^-$. A spectrum was recorded and the sample was subsequently illuminated for 8-20 s with a red LED (70 mW·cm^{-2}, Lumileds) at 633 nm for conversion of FADH•. Three measurements were averaged. To extract the absorption spectrum of FADH• in HsCRY1, the contribution of FAD_{ox} in the blue light-induced difference spectrum was removed by subtracting an absorption spectrum of HsCRY1 with FAD_{ox} after normalization to the respective molar extinction coefficients of ε_{580} = 4,800 M^{-1} cm^{-1} and ε_{450} = 11,300 M^{-1} cm^{-1}.[1-2] To extract the absorption spectrum of $FADH^-$, the blue & red light-induced difference spectrum was cleared from 20% remaining FADH• determined from the positive difference absorbance at above 550 nm. The concentration of FAD_{ox} in the difference spectra was determined under the assumption that only the FAD_{ox}, but not $FADH^-$ absorbs in the spectral

region of 475-505 nm. The sum of the cleared blue & red light-induced difference spectrum and the oxidized FAD spectrum, both normalized to molarity, yielded the absorbance spectrum of $FADH^-$.

Kinetics of the decay of FADH• were recorded at 630 nm with a sampling interval of 0.1 s at 10 °C under aerobic conditions. Samples, with a concentration of 80 µM HsCRY1 and 40 µM FAD, were illuminated for only 2 s at 454 nm (35 $mW \cdot cm^{-2}$) to minimize formation of $FADH^-$. Three technical replicates were performed to obtain the time constant.

FTIR Spectroscopy

Spectra were recorded on a Bruker IFS66/S and Bruker Vertex80, respectively, with a mercury cadmium telluride detector at a resolution of 2 cm^{-1} at 10 °C. A long pass filter was placed in front of the detector blocking light at above 2000 cm^{-1} to increase the signal-to-noise ratio and to protect the detector. Fourier transform was performed with a zero filling factor of four. For difference spectroscopy, 2.5 µL of **1a**:HsCRY1 or **1b**:HsCRY1 were placed between two BaF_2 windows and sealed with grease. The sample thickness was adjusted to lead to an absorbance of 0.8-1.2 at 1650 cm^{-1}. For each experiment, 1024 scans were measured before and after illuminating the sample with UV light for 20 s (355 nm, 10 Hz, 16 $mW \cdot cm^{-2}$) using a pulsed Nd:YAG laser (Qantel Ultra 100) and with a blue LED for 10 s (415 nm; 34 $mW \cdot cm^{-2}$, Lumileds), respectively. Spectra resulted from experiments on different technical replicates with a total number of 5,120 scans for **1b** in HsCRY1 illuminated with UV and blue light, respectively. For **1a** in HsCRY1, a total number of 6,144 scans for each, UV and blue light illumination, were averaged. Spectra obtained by blue light illumination were inverted and averaged with spectra obtained by UV light to obtain the difference spectrum corresponding to the *trans* → *cis*-isomerization. Difference spectra of **1a** and **1b** in DMSO were obtained by illumination with UV and blue light, respectively. Spectra resulted from 5,120 scans and 2,048 scans for **1a** and **1b**, respectively, for each illumination.

For measurements on HsCRY1 with FAD, 512 scans were acquired before and after illumination. For the blue light-induced difference spectrum, samples were illuminated for 300 ms at 453 nm (63 $mW \cdot cm^{-2}$) and a total number of 47,616 scans was averaged. The blue & red light-induced difference spectrum was obtained by illuminating the sample for 10 s at 453 nm (63 $mW \cdot cm^{-2}$) and 4 s at 633 nm (97 $mW \cdot cm^{-2}$). A total number of 1,536 scans was averaged.

Quantum Chemical Calculations

Quantum chemical calculations and geometry optimization were conducted using Gaussian16. Density functional theory (DFT) calculations of the *cis*- and *trans*-isomers of **1a** and **1b** were performed with the B3LYP functional and a 6-311+G(2d,p) basis set. The line spectrum of the calculated normal modes was broadened with Lorentzians with a full width at half maximum of 14 cm^{-1} and a single scaling factor of 0.98 was applied to the frequencies.

Difference spectra were obtained by subtracting the calculated spectrum of the compound in the *trans*-configuration from that in the *cis*-configuration.

For calculations on the twisted β-sheet of HsCRY1, backbone coordinates of the AlphaFold2 model of HsCRY1 (AF-Q16526-F1-v4) from the AlphaFold Protein Structure Database[3] were used, in which amino acid residues were replaced by alanine. Coordinates for the untwisted β-sheet of HsCRY1 were generated by rotating the twisted β-sheet around an axis of Cα-atoms of His7 (15°), Cys33 (0°), Ser97 (15-28°) and Ile125 (15-28°) and coordinates for the planar structure were obtained by rotating around an axis of Cα-atoms of His7 (26°), Cys33 (0°), Ser97 (25°) and Ile125 (37°). At the N- and C-terminal ends of the β-strands, the amide group of the Cα-atoms were replaced by a hydrogen and the corresponding Cβ-atoms were fixed for optimization. Normal modes of the twisted and untwisted β-sheet of HsCRY1 were calculated using DFT with BP86 functional with a 6-31G(d) basis set and a polarizable continuum model simulating water as solvent. The line spectrum of the calculated normal modes was broadened with Lorentzians with a full width at half maximum of 14 cm^{-1} and a single scaling factor of 1.0045 was applied to the wavenumbers. Difference spectra were obtained by subtracting the calculated spectra of the twisted β-sheet from that of the untwisted β-sheet.

$^{16}O/^{18}O$ Exchange of Azobenzenecarboxylic Acid **4**

The $^{16}O/^{18}O$ exchange was explored with the less expensive azobenzenecarboxylic acid **4a**. We applied the protocol of Seyfried *et al.*.[4] A suspension of azobenzenecarboxylic acid **4a**, 50 eq of $H_2{}^{18}O$, and 20 eq of 3,5-dimethylpyridine hydrobromide in DMF was stirred at room temperature and 30 eq of 1-ethyl-3-(3-dimethylaminopropyl)carbodiimide hydrochloride (EDC•HCl) were added in 3 portions over the course of 24 hours. After a total reaction time of two days, the ^{18}O-enrichments were only 42% and 36% in two independent experiments. These results differ significantly from the reported >90% ^{18}O-enrichments of *N*-Fmoc protected amino acids.[4] We noticed that azobenzenecarboxylic acid **4a** had not been completely dissolved, which may explain the results. The extension of the reaction time of the first experiment by five days increased the ^{18}O-enrichments only from 42% to 43%. In the case of the second experiment, we added, after the initial two days of reaction time, 10 eq of EDC•HCl and 20 eq of $H_2{}^{18}O$ and heated the suspension to 40 °C. Thereupon the suspension became a solution. This solution was stirred at 40 °C for three days. The ^{18}O-enrichment increased from 36% to 48%. This enrichment is insufficient for the requirements of the IR spectroscopic experiments.

Therefore, we switched from the non-equilibrium, multiple-turnover ^{18}O-labeling reaction[4] to a classic equilibrium-based ^{18}O-labeling reaction:[5] Azobenzene[^{13}C]carboxylic acid **4c** was treated with 50 eq of $H_2{}^{18}O$ and 4.4 eq of HCl in 1,4-dioxane at 100 °C for 17 h. Subsequently, all volatiles (HCl, dioxane, $H_2{}^{16}O$ and $H_2{}^{18}O$) were removed through freeze-drying. This process was repeated in the same vessel four times, achieving ^{18}O-enrichments of 75%, 93%, 95%, and finally 97%. This procedure is based on the protocol of Marecek *et al.*[5]

However, Marecek *et al.* utilized a 0.25 M solution of HCl in $H_2{}^{18}O$ prepared by reacting acetyl chloride with $H_2{}^{18}O$, whereas we opted for a 4 M solution of HCl in dioxane, which is commercially available. This change allowed us to eliminate one step requiring inert conditions and the use of acetyl chloride, which brings along an additional ^{16}O atom, which potentially effects the ^{18}O-enrichment. Furthermore, we used a solvent mixture of $H_2{}^{18}O$/dioxane in a 1:4 volume ratio instead of the 2:3 ratio used in the protocol of Marecek et al., as azobenzene[^{13}C]carboxylic acid **4c** was not soluble in the 2:3 mixture even at 100 °C. Furthermore, the 1:4 solvent ratio allowed the removal of volatiles with freeze-drying instead of vacuum distillation.

Determination of the Isotopic Enrichments

The ^{13}C and ^{18}O isotopic enrichments were determined from mass spectra by fitting the isotope patterns in the experimentally obtained spectra using LabelChecker in the "Universal Mass Calculator (UMC) (Version 3.14.0.47)".[6] The fitting process was performed as follows: The isotope patterns for the components possibly present in the material were calculated. These components included those with (a) natural isotope abundance of all atoms, (b) natural isotope abundance of all atoms, but 1 carbon atom being ^{13}C, (c) natural isotope abundance of all atoms, but 1 or 2 oxygen atoms being ^{18}O, and (d) natural isotope abundance of all atoms, but 1 carbon atom being ^{13}C and 1 or 2 oxygen atoms being ^{18}O. These patterns were then combined with the weights needed to reproduce the measured pattern with minimal deviation using a simplex algorithm.[7] From the weights, which correspond to the proportions of the species, the isotopic enrichments were calculated. To give an example, Figure S11 presents the experimentally determined mass spectrum of the $[M + H]^+$ ion of photo-pharmacological compound **1b** and the calculated mass spectrum for this ion with 99% of ^{13}C and 97% of ^{18}O enrichment. The close similarity of the isotopic distributions let us report the isotopic enrichments 99% of ^{13}C and 97% of ^{18}O for photopharmacological compound **1b**.

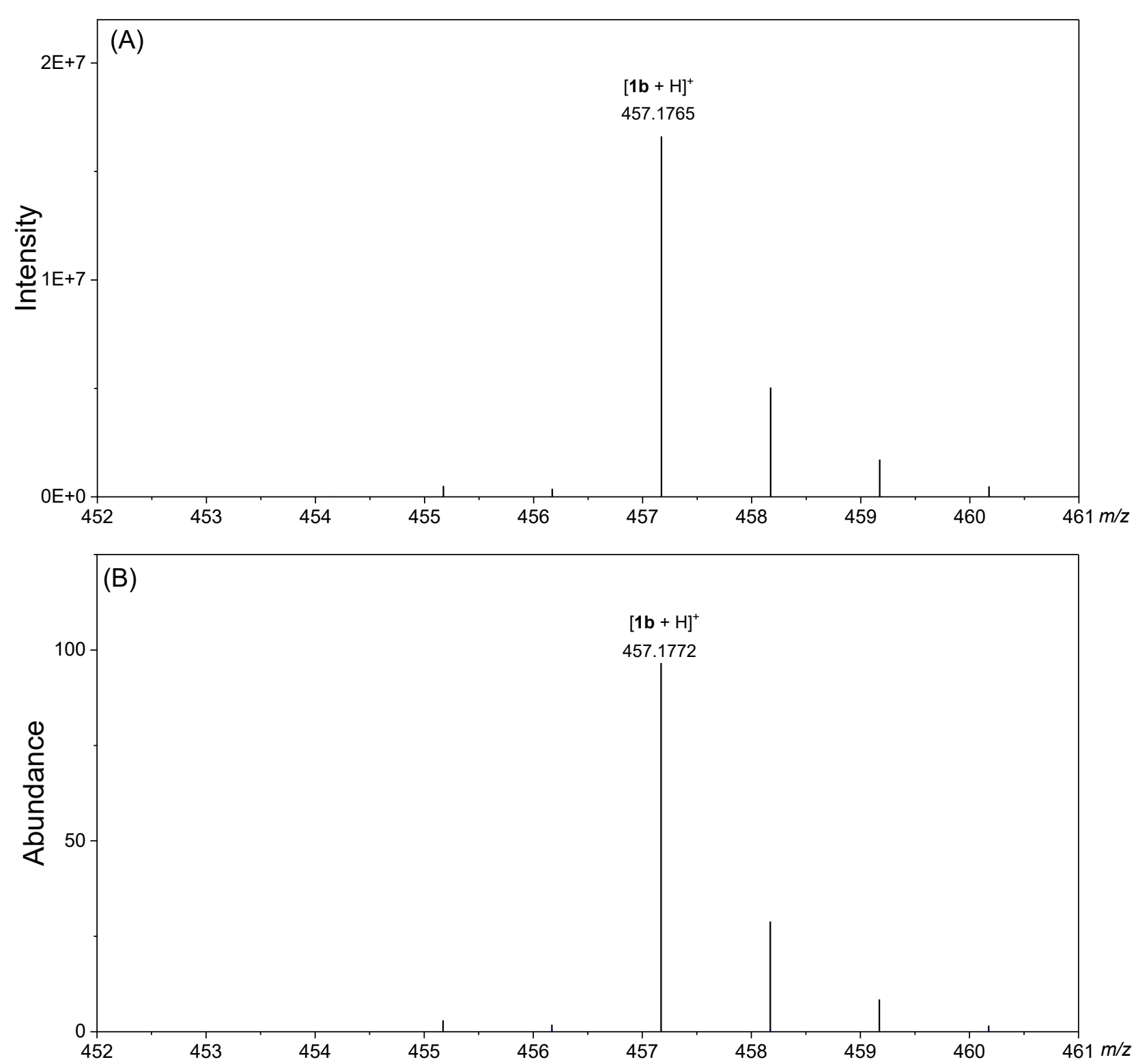

Figure S11. Determination of the isotopic enrichment of **1b** from mass spectra. (A) Experimentally obtained mass spectrum for the $[M + H]^+$ ion of photopharmacological compound **1b**. (B) Calculated mass spectrum of the $[M + H]^+$ ion of photopharmacological compound **1b** with a ^{13}C enrichment of 99% and an ^{18}O enrichment of 97%.

Signals of the *Z*-Isomer of Photopharmacological Compounds **1a** and **1b** in the NMR Spectra

The ^{1}H and ^{13}C NMR spectra of photopharmacological compounds **1a**,**b** (Figure S22 and S27) show several low-intensity signals in addition to the signals of the photo-pharmacological compounds. We assign these signals to the *Z*-isomer of the photo-pharmacological compounds based on the following finding: A solution of photo-pharmacological compound **1a** in CD_2Cl_2 was freshly prepared and the ^{1}H and ^{13}C NMR spectra were recorded. The ^{1}H NMR and ^{13}C NMR spectra are shown in Figure S12A and S13A, in which arrows point out the signals of the *Z*-isomer. The solution was then illuminated with light with a wavelength of 385 nm for five minutes and the ^{1}H and ^{13}C NMR spectra were recorded immediately after. The ^{1}H NMR and the ^{13}C NMR spectra (Figure S12B and S13B) show the signals assigned to the *Z*-isomer with clearly increased intensity. Subsequently, the sample was stored in the dark for six days. The then recorded ^{1}H NMR and ^{13}C NMR spectra (Figure S12C and S13C) are nearly identical to the ones of the sample before illumination. Illumination with light with a wavelength of 385 nm causes partial, photochemical isomerization of the *E*-isomer to the *Z*-isomer and upon storing the sample in the dark a thermal isomerization brings the sample back into equilibrium of these isomers.

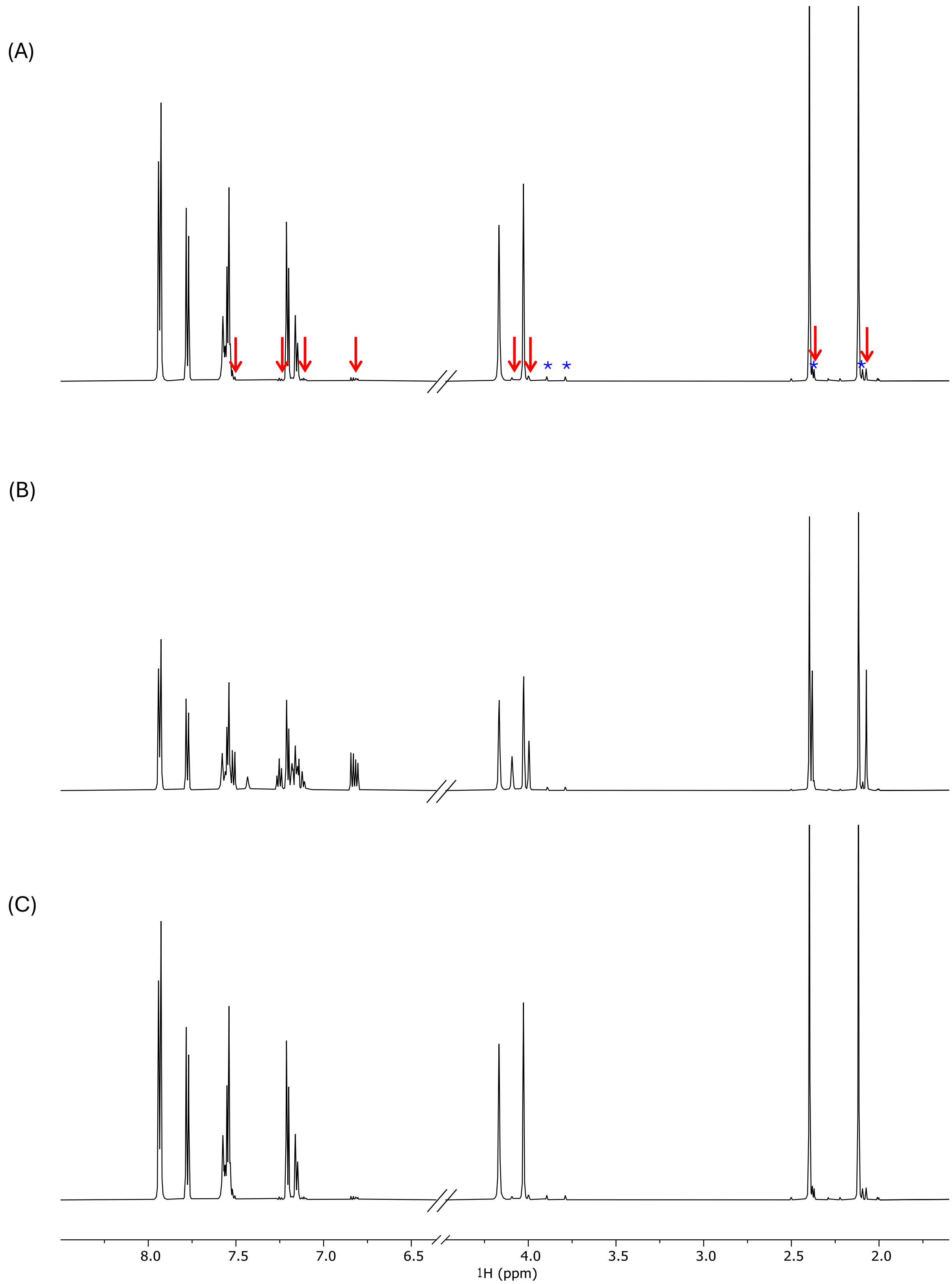

Figure S12. Cutouts of the ^{1}H NMR spectra (600 MHz, CD_2Cl_2) of photopharmacological compound **1a**: (A) freshly prepared sample, (B) after illumination with light of a wavelength of 385 nm for 5 min, and (C) after storage of the illuminated sample in the dark for 6 days. Arrows indicate the signals corresponding to the *Z*-isomer seen in the spectrum of the material before irradiation. The asterisk (*) marks signals of unidentified compounds.

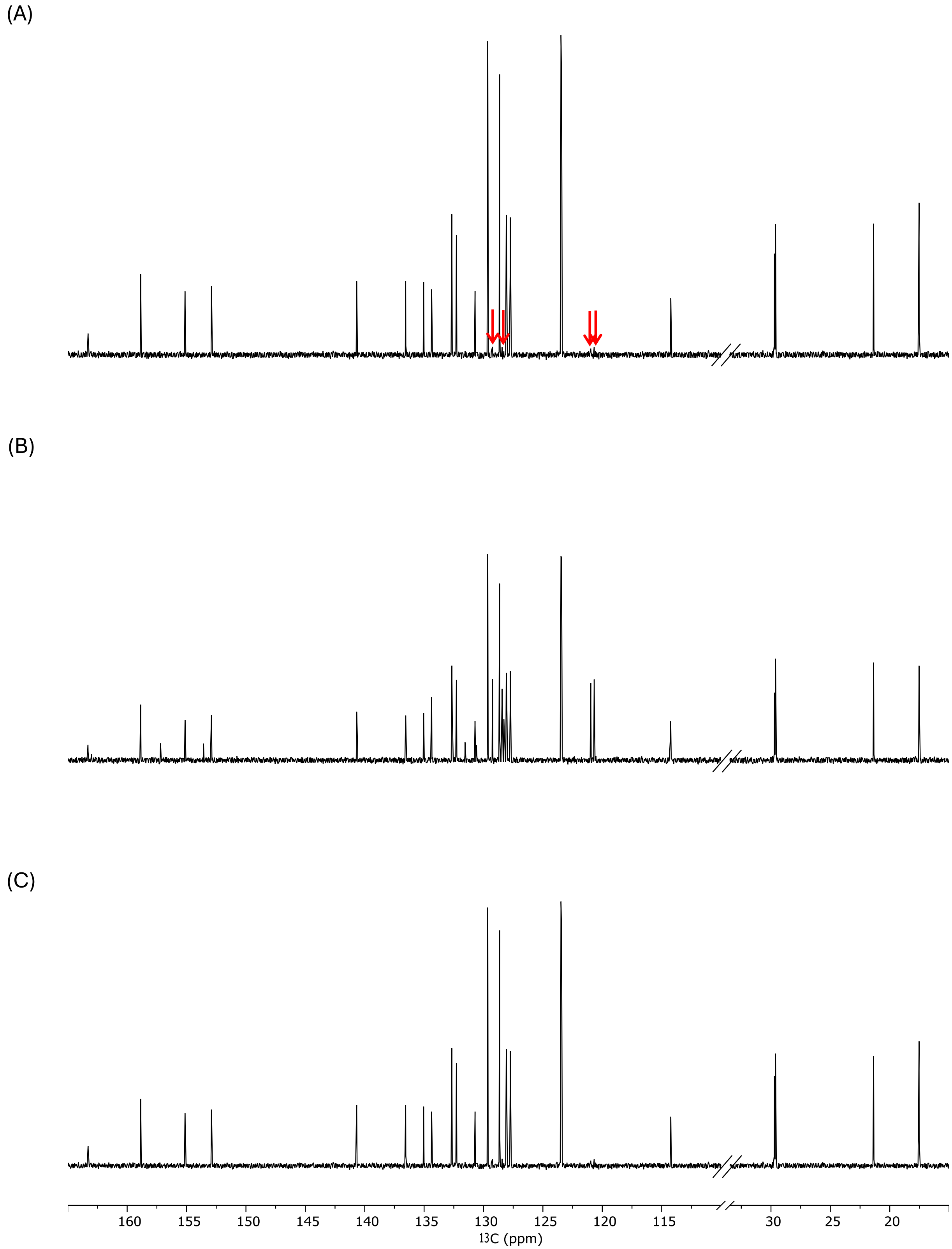

Figure S13. Cutouts of the ^{13}C NMR spectra (600 MHz, CD_2Cl_2) of photopharmacological compound **1a**: (A) freshly prepared sample, (B) after illumination with light of a wavelength of 385 nm for 5 min, and (C) after storage of the illuminated sample in the dark for 6 days. Arrows indicate the signals corresponding to the *Z*-isomer seen in the spectrum of the material before irradiation.

Synthesis of Photopharmacological Compounds **1a** and **1b**

General. Unless otherwise stated, reactions were performed using commercial solvents and reagents. 4-Aminobenzoic [^{13}C]acid (Merck, chemical purity ≥99%, ^{13}C-enrichment ≥99%), $H_2^{18}O$ (^{18}O-enrichment 97%, deutero GmbH), 4-cyano-3-oxotetrahydrothiophene (98%, TCI), (2,4-dimethylphenyl)hydrazine hydrochloride (>99%, BLDpharm), 4-aminobenzoic acid methyl ester (Oxchem, 98%), nitrosobenzene (TCI, >98%), $SOCl_2$ (>99.5%, Acros Organics), 1-ethyl-3-(3-dimethylaminopropyl)carbodiimide hydrochloride (EDC•HCl; 99%, BLDpharm). 3,5-dimethylpyridine (>98%, Thermo Scientific), 4 M HCl in 1,4-dioxane (TCI), $LiOH•H_2O$ (>99%, Roth), NaOH (99%, VWR), aqueous HCl (37 wt.%, p.a., VWR), aqueous HBr (48 wt.%, Riedel-de Haën AG), H_2O (Milli-Q), NaCl (99.9%, VWR), $NaHCO_3$ (≥99.7%, Fisher Scientific), Na_2SO_4 (technical grade), acetic acid (99.9%, VWR), absolute EtOH (99.8%, Fisher Scientific), MeOH (99.9%, HPLC grade, VWR), anhydrous MeOH (99.8%, extra dry over molecular sieve, Acros Organics), CH_2Cl_2 (99.9%, HPLC grade, VWR), anhydrous DMF (99.8%, extra dry over molecular sieve, Acros Organics), MeCN (HPLC grade, Honeywell), anhydrous MeCN (99.9%, extra dry over molecular sieve, Thermo Scientific). Et_3N (p.a. grade) was dried with CaH_2 prior to its use. THF (HPLC grade) was dried with sodium/benzophenone prior to its use. Solvents used for extraction and chromatography were purchased in technical quality and distilled prior to use. Since all commercial compounds had a purity of >95%, their amount of substance given in the preparation procedures was calculated using the weight of the compound without correction by the manufacturer-specified purities.

The composition of solvent mixtures is given in volume ratios. The pH of aqueous solutions was determined using pH indicator strips with a resolution of 0.2 or 0.5 pH. The temperatures given in the preparative procedures are the bath temperatures.

If not otherwise stated, the reaction was run in air. Argon (Linde, 4.6) was passed through anhydrous $CaCl_2$ prior to use. Degassed solutions were obtained through three freeze−pump−thaw cycles and ventilation of the flask with argon. Unless otherwise stated, solvents were removed at ~ 40 °C and reduced pressure using a rotary evaporator and traces of remaining solvents were removed at room temperature at <1 mbar.

Thin layer chromatography was carried out on silica gel coated aluminum foil (Merck, 60 F254) and the chromatogram was analyzed under a UV lamp with λ = 254 nm. For column chromatography, silica gel 60 M (0.040 – 0.063 mm, Macherey-Nagel), was used as the stationary phase and slight pressure was applied. The size of the column is given as diameter × length.

HPLC−MS measurements were performed on a Shimadzu 2040 HPLC system coupled with a Shimadzu LCMS 2050 mass spectrometer, equipped with a heated DUIS (dual ion ESI/APCI Source), an autosampler, degasser, quaternary low-pressure gradient pump, column oven, photodiode array detector, and a C18 column (Phenomenex Kinetex C18, particle size 2.6 µm, pore size 100 Å, column size 2.1 mm × 100 mm) with a column oven temperature of 40 °C. The DUIS operated at a desolvation temperature of 450 °C with a desolvation line

temperature of 200 °C. For ionization, the interface voltage was set at 3.0 kV for positive mode and -2.0 kV for negative mode. Nitrogen, provided by the nitrogen generator NGA CASTORE XS iQ 18, was used as the nebulizer gas (nebulizing flow 2 L/min), the dry gas (drying gas flow 5 L/min), and the heating gas (heating gas flow 7 L/min). The mobile phase had a flow rate of 0.45 mL/min and a linear gradient consisting of A (H_2O/MeCN/HCO_2H, 950:50:1) and B (MeCN/H_2O/HCO_2H, 950:50:1) with the following percentages of B: 0.00−0.50 min, 0%; 0.50−5.50 min, 0%−100%; 5.50−6.00 min, 100%; 6.00−6.01 min, 100%−0%; 6.01−10.00 min, 0%. Typically, 5 µL solution of the analyte (∼1 mM) dissolved in water/acetonitrile were injected.

Accurate Mass (ESI) measurements are performed using a Q-IMS-TOF mass spectrometer Synapt G2Si (Waters GmbH, Manchester, UK) in resolution mode, interfaced to a nano-ESI ion source. Nitrogen serves both as the nebulizer gas and the dry gas. Nitrogen is generated by a nitrogen generator NGM 11. Helium 5.0 is used as buffer gas in the IMS entry cell, nitrogen 5.0 is used for IMS separations. Samples were dissolved in MeCN and introduced by static nano-ESI using in-house pulled glass emitters.

NMR spectra were calibrated using the signal of the solvent as an internal standard [$CDCl_3$: δ(^{1}H of $CHCl_3$) = 7.26 ppm, δ(^{13}C of $CDCl_3$) = 77.16 ppm; CD_2Cl_2: δ(^{1}H of $CHDCl_2$) = 5.32 ppm, δ(^{13}C of CD_2Cl_2) = 53.84 ppm; DMSO-d_6: δ(^{1}H of DMSO-d_5) = 2.50 ppm, δ(^{13}C of DMSO-d_6) = 39.52 ppm. NMR signal assignment is supported by ^{13}C{^{1}H} DEPT 135, ^{1}H,^{1}H-gCOSY, ^{1}H,^{13}C-gHMQC, and ^{1}H,^{13}C-gHMBC NMR experiments. For the assignment of the NMR signals, the hydrogen (H) and carbon (C) atoms of the dimethylphenyl unit are denoted as $H_{dimethylphenyl}$ and $C_{dimethylphenyl}$, the H and C atoms of the phenylene (C_6H_4) unit are denoted as $H_{phenylene}$ and $C_{phenylene}$, and the H and C atoms of the phenyl (C_6H_5) unit are denoted as H_{phenyl} and C_{phenyl}.

Methyl (*E*)-azobenzene-4-carboxylate (3a) This experiment was performed following the reported procedure[8] with minor changes. Methyl 4-aminobenzoate (**2a**; 2.83 g, 18.7 mmol) and nitrosobenzene (2.02 g, 18.8 mmol) were dissolved in acetic acid (37.4 mL) and stirred at room temperature for 24 hours. Removal of all volatiles gave a red-brown solid. This solid was suspended in methanol (47.5 mL) and the suspension was heated to reflux. The suspension was slowly cooled to room temperature, during which orange crystals precipitated. Once the suspension reached room temperature, it was further cooled with an ice-water bath, causing additional crystal formation. The precipitated crystals were collected by filtration and washed with cold methanol and dried at reduced pressure. This gave methyl (*E*)-azobenzene-4-carboxylate (**3a**; 3.52 g, 78%) as orange-red crystals. ^{1}H NMR (500 MHz, $CDCl_3$): δ [ppm] = 8.19 (one half of an AA'XX' spin system, 2 H, $H_{phenylene}$ *ortho* to CO_2Me), 7.98 – 7.92 (m, 4 H, overlapping signals of $H_{phenylene}$ *meta* to CO_2Me and H_{phenyl} *ortho* to N), 7.56 – 7.48 (m, 3 H, overlapping signals of H_{phenyl} *para* and *meta* to N), 3.96 (s, 3 H, CH_3). ^{13}C{^{1}H} NMR (126 MHz, $CDCl_3$): δ [ppm] = 166.7 (C=O), 155.3 ($C_{phenylene}$-N), 152.7 (NC_{phenyl}), 131.9 ($MeO_2C\underline{C}_{phenylene}$), 131.8 ($C_{phenyl}$ *para* to N), 130.8 ($C_{phenylene}$ *ortho* to CO_2Me), 129.3 (C_{phenyl} *meta* to N), 123.3 (C_{phenyl} *ortho* to N), 122.8 ($C_{phenylene}$ *meta* to CO_2Me), 52.5 (Me).

(*E*)-Azobenzene-4-carboxylic acid (4a) This experiment was performed following the reported procedure[9] with minor changes. Methyl (*E*)-azobenzene-4-carboxylate (**3a**; 1.51 g, 6.29 mmol) was dissolved in THF (50 mL) and a solution of $LiOH•H_2O$ (0.53 g, 12.6 mmol) in H_2O (25 mL) was added. The resulting orange-red solution was stirred at room temperature for 4 hours. 1.0 M aqueous HCl (13.8 mL) was added and the mixture was extracted with EtOAc (2 × 70 mL). The combined organic phases were washed with saturated aqueous NaCl solution (2 × 30 mL), dried over Na_2SO_4, and filtered. Removal of the solvent yielded (*E*)-4-azobenzene-4-carboxylic acid (**4a**; 1.41 g, 99%) as an orange solid. ^1H NMR (500 MHz, DMSO-d6): δ [ppm] = 13.25 (br s, 1 H, CO_2H), 8.15 (one half of an AA'XX' spin system, 2 H, $H_{phenylene}$ *ortho* to CO_2H), 7.97 (other half of the AA'XX' spin system, 2 H, $H_{phenylene}$ *meta* to CO_2H), 7.95 – 7.91 (m, 2 H, H_{phenyl} *ortho* to N), 7.65 – 7.59 (m, 3 H, overlapping signals of H_{phenyl} *para* and *meta* to N). $^{13}C\{^1H\}$ NMR (126 MHz, DMSO-d6): δ [ppm] = 166.7 (C=O), 154.3 ($C_{phenylene}$-N), 151.9 (NC_{phenyl}), 132.9 ($HO_2C\underline{C}_{phenylene}$), 132.2 ($C_{phenyl}$ *para* to N), 130.6 ($C_{phenylene}$ *ortho* to CO_2H), 129.6 (C_{phenyl} *meta* to N), 122.8 (C_{phenyl} *ortho* to N), 122.6 ($C_{phenylene}$ *meta* to CO_2H). MS (DUIS) *m/z* = 227.2 $[M + H]^+$, 225.0 $[M - H]^-$.

Pyrazolamine 5. Pyrazolamine **5** was synthesized by following the reported procedure[10] with minor changes. 4-Cyano-3-oxotetrahydrothiophene (3.15 g, 24.8 mmol) and (2,4-dimethylphenyl)hydrazine hydrochloride (4.58 g, 26.5 mmol) were suspended in absolute ethanol (119 mL). The resulting orange suspension was heated to reflux. Within 30 minutes, it transformed into an orange solution. This solution was further stirred under reflux for an additional 135 minutes. Removal of all volatiles gave an orange-brown solid (7.88 g). This solid was dissolved in CH_2Cl_2 (60 mL) and the solution was washed with 1.0 M aqueous NaOH solution (30 mL) and saturated aqueous NaCl solution (40 mL), dried over Na_2SO_4, and filtered. Removal of the solvent yielded pyrazolamine **5** (5.58 g, 92%) as a pale orange-brown solid. ^1H NMR (500 MHz, $CDCl_3$): δ [ppm] = 7.15 (d, $^3J_{HH}$ = 7.9 Hz, 1 H, $H_{dimethylphenyl}$ *ortho* to N), 7.13 (m, 1 H, $H_{dimethylphenyl}$ *ortho* to both methyl groups), 7.09 (doublet of multiplet, $^3J_{HH}$ = 7.9 Hz, 1 H, $H_{dimethylphenyl}$ *para* to Me), 3.95 (t, $^4J_{HH}$ = 1.6 Hz, 2 H, $N_{pyrazole}$=$C_{pyrazole}CH_2S$), 3.78 (t, $^4J_{HH}$ = 1.6 Hz, 2 H, $SCH_2C_{pyrazole}$=$C_{pyrazole}$), 3.51 (br s, 2 H, NH_2), 2.36 (s, 3 H, CH_3 *para* to N), 2.12 (s, 3 H, CH_3 *ortho* to N). $^{13}C\{^1H\}$ NMR (126 MHz, $CDCl_3$): δ [ppm] = 158.1 ($C_{pyrazole}$=$N_{pyrazole}$), 139.6 ($Me\underline{C}_{dimethylphenyl}$ *para* to N), 139.4 ($C_{pyrazole}$-NH_2), 136.4 ($Me\underline{C}_{dimethylphenyl}$ *ortho* to N), 134.3 ($\underline{C}_{dimethylphenyl}N$), 132.0 ($HC_{dimethylphenyl}$ *ortho* to both methyl groups), 127.9 ($HC_{dimethylphenyl}$ *ortho* to N), 127.6 ($HC_{dimethylphenyl}$ *para* to Me), 104.8 ($\underline{C_{pyrazole}}$=$C_{pyrazole}$-NH_2), 29.7 ($N_{pyrazole}$=$C_{pyrazole}\underline{C}H_2S$), 27.1 ($S\underline{C}H_2C_{pyrazole}$=$C_{pyrazole}$), 21.3 ($CH_3$ *para* to N), 17.5 (CH_3 *ortho* to N). MS (DUIS) *m/z* = 246.1 $[M + H]^+$.

Photopharmacological compound 1a. This reaction was carried out under argon and following the reported procedure[9] with changes. (*E*)-4-azobenzene-4-carboxylic acid (**4a**; 1.01 g, 4.46 mmol) was suspended in $SOCl_2$ (11 mL) and the red suspension was stirred at 80 °C for 1.5 hours. Then all volatiles were removed under reduced pressure by a short path distillation and the residual orange solid was dissolved in anhydrous CH_2Cl_2 (30 mL). Pyrazolamine **5** (732 mg, 2.98 mmol) and Et_3N (3 mL) were added. The dark red solution was stirred at room temperature for 21 hours. During this time a colorless precipitate formed and the color of the mixture changed from dark red to red-orange. 1 M aqueous HCl (20 mL) was added to the reaction mixture. After thorough mixing,

the organic and the aqueous phases were separated. The aqueous phase was extracted with CH_2Cl_2 (2 × 90 mL). The combined organic phases were washed with saturated aqueous NaCl solution (10 mL), dried over Na_2SO_4, and filtered. Removal of the solvents gave a red-orange solid (1.55 g). The red-orange solid was suspended in EtOH (55 mL) under reflux, the dark-red suspension was slowly cooled to room temperature and then cooled to 5 °C and kept at 5 °C for 13 hours. Within this time an orange precipitate formed. The precipitate (0.900 g) was collected by filtration and suspended in EtOH (16 mL) under reflux. The suspension was then cooled to 5 °C and kept at this temperature for 11 hours. The orange precipitate (0.829 g) was isolated by filtration. The orange precipitate was dissolved in EtOAc/CH_2Cl_2 (2:1, 900 mL), silica gel (4.54 g) was added, and the solvents were removed. The silica gel with the adsorbed material was loaded onto a flash chromatography column (3 cm × 30 cm). Chromatography with CH_2Cl_2/EtOH (40:1) as the eluent gave photopharmacological compound **1a** (0.529 g, 39%) as an orange solid. ^{1}H NMR (600 MHz, CD_2Cl_2): δ [ppm] = 7.95 – 7.91 (m, 4 H, overlapping signals of $H_{phenylene}$ *meta* to C=O and H_{phenyl} *para* to N), 7.78 (one half of an AA'XX' spin system, 2 H, $H_{phenylene}$ *ortho* to C=O), 7.59 – 7.52 (m, 3 H, overlapping signals of H_{phenyl} *para* and *meta* to N), 7.22 – 7.19 (m, 2 H, overlapping signals of $H_{dimethylphenyl}$ *ortho* to N and $H_{dimethylphenyl}$ *ortho* to both methyl groups), 7.17 – 7.14 (m, 1 H, $H_{dimethylphenyl}$ *para* to Me), 4.17 (s, 2 H, $N_{pyrazole}$=$C_{pyrazole}CH_2S$), 4.03 (s, 2 H, $SCH_2C_{pyrazole}$=$C_{pyrazole}$), 2.40 (s, 3 H, CH_3 *para* to N), 2.12 (s, 3 H, CH_3 *ortho* to N). $^{13}C\{^1H\}$ NMR (151 MHz, CD_2Cl_2): δ [ppm] = 163.3 (C=O), 158.9 (C=N), 155.1 ($C_{phenylene}$-N), 152.9 (NC_{phenyl}), 140.7 (Me$\underline{C}_{dimethylphenyl}$ *para* to N), 136.6 (Me$\underline{C}_{dimethylphenyl}$ *ortho* to N), 135.0 (O=C$\underline{C}_{phenylene}$), 134.4 ($\underline{C}_{dimethylphenyl}$N), 132.7 ($HC_{dimethylphenyl}$ *ortho* to both methyl groups), 132.3 (C_{phenyl} *para* to N), 130.7 ($C_{pyrazole}$N), 129.6 (C_{phenyl} *meta* to N), 128.6 ($C_{phenylene}$ *ortho* to C=O), 128.1 ($HC_{dimethylphenyl}$ *para* to Me), 127.8 ($HC_{dimethylphenyl}$ *ortho* to N), 123.48 (C_{phenyl} *ortho* to N), 123.43 ($C_{phenylene}$ *meta* to C=O), 114.2 ($\underline{C}_{pyrazole}$=$C_{pyrazole}$-NH), 29.7 and 29.6 ($\underline{C}H_2S$), 21.4 (CH_3 *para* to N), 17.5 (CH_3 *ortho* to N). Accurate MS (ESI): *m/z* calcd. for $[M + H]^+$ $C_{26}H_{24}N_5OS^+$: 454.1696; found: 454.1695.

Methyl 4-aminobenzene-1-[^{13}C]carboxylate (2b). This reaction was carried out under argon. 4-Aminobenzoic [^{13}C]acid (100 mg, 724 μmol) was dissolved in anhydrous MeOH (3 mL). Under stirring and cooling with an ice-water bath $SOCl_2$ (0.16 mL, 2.20 mmol) was added dropwise over 3 minutes whereupon the reaction solution turned into a suspension. The suspension was stirred under cooling für 37 minutes, the ice-water bath was removed, and the suspension was stirred at room temperature for 17 hours. During this time the suspension turned into a solution. CH_2Cl_2 (3 mL), H_2O (3 mL), and saturated aqueous $NaHCO_3$ (7 mL) were added successively. The pH of the aqueous phase was 8. After separation of the phases, the aqueous phase was extracted with CH_2Cl_2 (5 × 5 mL). The combined organic phases were washed with H_2O (10 mL), dried with Na_2SO_4, and filtered. Removal of the solvents gave methyl 4-aminobenzene-1-[^{13}C]carboxylate (**2b**; 118 mg, 98%) along with a very small amount of unidentified compounds as a beige solid. ^{1}H NMR (600 MHz, $CDCl_3$): δ [ppm] = 7.5 (one half of AA'XX' spin system split into d through coupling with ^{13}C, see Figure S23a, 2 H, $H_{phenylene}$ *ortho* to CO_2Me), 7.64 (other half of the AA'XX' spinsystem sligthly broadened, 2 H, $H_{phenylene}$ *meta* to CO_2Me), 4.06 (br s, 2 H, NH_2), 3.85 (d, 3 H, $^3J_{HC}$ = 3.8 Hz, CH_3). $^{13}C\{^1H\}$ NMR (151 MHz, $CDCl_3$): δ [ppm] = 167.3 (C=O), 150.9 ($C_{phenylene}$-N), 131.7 (d, $^2J_{CC}$

= 2.8 Hz, $C_{phenylene}$ *ortho* to CO_2Me), 119.9 (d, ${}^1J_{CC}$ = 78.1 Hz, MeO_2C-$\underline{C}_{phenylene}$), 113.9 (d, ${}^2J_{CC}$ = 4.8 Hz, $C_{phenylene}$ *meta* to CO_2Me), 51.7 (d, ${}^2J_{CC}$ = 2.5 Hz, Me). Accurate MS (ESI): *m/z* calcd. for $[M + H]^+$ $C_7{}^{13}CH_{10}NO_2{}^+$: 153.0740; found: 153.0750.

Methyl (*E*)-azobenzene-4-[^{13}C]carboxylate (3b). Methyl 4-aminobenzene-1-[^{13}C]carboxylate (**2b**; 108 mg, 710 µmol) and nitrosobenzene (77 mg, 719 µmol) were dissolved in acetic acid (3 mL) and the solution was stirred at room temperature for 17.5 hours. During this time the reaction mixture turned from a green solution into a yellow-brown suspension. A small amount of the reaction mixture was taken, dried under reduced pressure, and analyzed via ^{1}H NMR spectroscopy, which indicated that the conversion of methyl 4-aminobenzene-1-[^{13}C]carboxylate (**2b**) was incomplete. Nitrosobenzene (39 mg, 364 µmol) was added to the reaction suspension and the suspension was stirred at room temperature for 23.5 hours. Removal of all volatiles gave methyl (*E*)-azobenzene-4-[^{13}C]carboxylate (**3b**; 168 mg, 98%) along with a very small amount of unidentified compounds as a brown-gold solid. ^{1}H NMR (600 MHz, CD_2Cl_2): δ [ppm] = 8.19 (one half of AA'XX' spin system split into d through coupling with ^{13}C, see Figure S24a, 2 H, $H_{phenylene}$ *ortho* to CO_2Me), 7.98 – 7.93 (m, 4 H, overlapping signals of $H_{phenylene}$ *meta* to CO_2Me and H_{phenyl} *ortho* to N), 7.58 – 7.51 (m, 3 H, overlapping signals of H_{phenyl} *para* and *meta* to N), 3.94 (d, ${}^3J_{HC}$ = 3.8 Hz, CH_3). $^{13}C\{^1H\}$ NMR (126 MHz, $CDCl_3$): δ [ppm] = 166.7 (C=O), 155.5 ($C_{phenylene}$-N), 153.0 (NC_{phenyl}), 132.4 (d, ${}^1J_{CC}$ = 75.2 Hz, $MeO_2C\underline{C}_{phenylene}$), 132.15 ($C_{phenyl}$ *para* to N), 130.91 (d, ${}^2J_{CC}$ = 2.5 Hz, $C_{phenylene}$ *ortho* to CO_2Me), 129.6 (C_{phenyl} *meta* to N), 123.5 (C_{phenyl} *ortho* to N), 123.0 (d, ${}^3J_{CC}$ = 4.9 Hz, $C_{phenylene}$ *meta* to CO_2Me), 52.6 (d, ${}^2J_{CC}$ = 2.5, Me). Accurate MS (ESI): *m/z* calcd. for $[M + H]^+$ $C_{13}{}^{13}CH_{13}N_2O_2{}^+$: 242.1005; found: 242.1015.

(*E*)-Azobenzene-4-[^{13}C]carboxylic acid (4c). Methyl (*E*)-azobenzene-4-[^{13}C]carboxylate (**3b**; 168 mg, 696 µmol) was dissolved in THF (6 mL) and H_2O (2 mL). A solution of LiOH•H_2O (62 mg, 1.5 mmol) in H_2O (3 mL) was added. The resulting solution was stirred at room temperature for 4 hours. 1.0 M aqueous HCl (1.5 mL) was added to reduce the pH of the solution to 2 and the mixture was extracted with EtOAc (1 × 10 mL, 2 × 5 mL). The combined organic phases were washed with H_2O (2 × 5 mL) and saturated aqueous NaCl solution (1 × 5 mL), dried over Na_2SO_4, and filtered. Removal of the solvents yielded (*E*)-azobenzene-4-[^{13}C]carboxylic acid (**4c**; 154 mg, 97%) along with a very small amount of unidentified compounds as an orange-brown solid. ^{1}H NMR (500 MHz, DMSO-d6): δ [ppm] = 13.23 (br s, 1 H, CO_2H), 8.15 (one half of AA'XX' spin system split into d through coupling with ^{13}C, see Figure S25a, 2 H, $H_{phenylene}$ *ortho* to CO_2H), 7.98 (other half of the AA'XX' spinsystem sligthly broadened, 2 H, $H_{phenylene}$ *meta* to CO_2H), 7.96 – 7.92 (m, 2 H, H_{phenyl} *ortho* to N), 7.66 – 7.59 (m, 3 H, overlapping signals of H_{phenyl} *para* and *meta* to N). $^{13}C\{^1H\}$ NMR (126 MHz, DMSO-d6): δ [ppm] = 166.7 (C=O), 154.3 ($C_{phenylene}$-N), 151.9 (NC_{phenyl}), 132.8 (d, ${}^1J_{CC}$ = 71.6 Hz, $HO_2C\underline{C}_{phenylene}$), 132.2 ($C_{phenyl}$ *para* to N), 130.7 (d, ${}^2J_{CC}$ = 2.7 Hz, $C_{phenylene}$ *ortho* to CO_2H), 129.6 (C_{phenyl} *meta* to N), 122.8 (C_{phenyl} *ortho* to N), 122.6 (d, ${}^3J_{CC}$ = 4.5 Hz, $C_{phenylene}$ *meta* to CO_2H). Accurate MS (ESI): *m/z* calcd. for $[M + H]^+$ $C_{12}{}^{13}CH_{11}N_2O_2{}^+$: 228.0849; found: 228.0859.

(*E*)-Azobenzene-4-[^{13}C,$^{18}O_2$]carboxylic acid (4d). This reaction was carried out under argon. (*E*)-Azobenzene-4-[^{13}C]carboxylic acid (**4c**; 50 mg, 220 µmol) was suspended in anhydrous 1,4-dioxane (0.96 mL). $H_2{}^{18}O$ (0.25 mL, 14 mmol) and 4 M solution of HCl in 1,4-dioxane (15.3 µL, 61.2 µmol) were added. The resulting orange suspension was stirred at 100 °C for 17 hours: Shortly after the bath had reached 100 °C, the orange suspension turned into a red solution. The volatile components were removed at room temperature through freeze-drying, giving an orange-brown solid. This procedure was applied all over four times in the same vessel. After each run an HPLC-MS analysis was performed and revealed ^{18}O-enrichments of 75%, 93%, 95%, and 97%, respectively. (*E*)-Azobenzene-4-[^{13}C,$^{18}O_2$]carboxylic acid (**4d**; 46 mg, yield 92%, ^{13}C-enrichment 99%, ^{18}O enrichment 97%) was obtained contaminated with a very small amount of unidentified compounds as an orange-brown solid. ^1H NMR (500 MHz, DMSO-d6): δ [ppm] = 13.20 (br s, 1 H, CO_2H), 8.15 (one half of AA'XX' spin system split into d through coupling with ^{13}C, see Figure S26a, 2 H, $H_{phenylene}$ *ortho* to CO_2H), 7.97 (other half of the AA'XX' spinsystem sligthly broadened, 2 H, $H_{phenylene}$ *meta* to CO_2H), 7.96 – 7.92 (m, 2 H, H_{phenyl} *ortho* to N), 7.66 – 7.59 (m, 3 H, overlapping signals of H_{phenyl} *para* and *meta* to N). $^{13}C\{^1H\}$ NMR (126 MHz, DMSO-d6): δ [ppm] = 166.6 (C=O), 154.3 ($C_{phenylene}$-N), 151.9 (NC_{phenyl}), 132.8 (d, $^1J_{CC}$ = 71.6 Hz, $HO_2C\underline{C}_{phenylene}$), 132.2 ($C_{phenyl}$ *para* to N), 130.6 (d, $^2J_{CC}$ = 2.7 Hz, $C_{phenylene}$ *ortho* to CO_2H), 129.6 (C_{phenyl} *meta* to N), 122.8 (C_{phenyl} *ortho* to N), 122.6 (d, $^3J_{CC}$ = 4.5 Hz, $C_{phenylene}$ *meta* to CO_2H). Accurate MS (ESI): *m/z* calcd. for $[M + H]^+$ $C_{12}{}^{13}CH_{11}N_2{}^{18}O_2{}^+$: 232.0934; found: 232.0944.

Photopharmacological compound 1b. (*E*)-Azobenzene-4-[^{13}C,$^{18}O_2$]carboxylic acid (**4d**; 46 mg, 200 µmol) was suspended in $SOCl_2$ (560 µL) and the red suspension was stirred at 80 °C for 1.5 hours. Shortly after the bath had reached 80 °C the orange suspension turned into a red solution. All volatiles were removed under reduced pressure by a short path distillation and the residual orange solid was dissolved in anhydrous CH_2Cl_2 (1.1 mL). Pyrazolamine **5** (59 mg, 240 µmol) and Et_3N (200 µL) were added. The solution was stirred at room temperature for 18 hours. During this time the reaction mixture turned from a solution into a suspension. 1 M aqueous HCl (1 mL), CH_2Cl_2 (5 mL), and H_2O (5 mL) were added to the reaction mixture. After thorough mixing, the organic and the aqueous phases were separated. The aqueous phase was extracted with CH_2Cl_2 (2 × 5 mL). The combined organic phases were washed with H_2O (3 × 5 mL), dried over Na_2SO_4, and filtered. Removal of the solvents gave an orange-brown solid (119 mg). Column chromatography (2 cm × 31 cm, CH_2Cl_2/EtOAc, 10:1) provided photopharmacological compound **1b** (21 mg, yield 23%, ^{18}O-enrichment 97%, ^{13}C-enrichment 99%; R_f = 0.45) as an orange solid. ^1H NMR (600 MHz, CD_2Cl_2): δ [ppm] = 7.93 (m, 4 H, overlapping signals of $H_{phenylene}$ *meta* to C=O and H_{phenyl} *para* to N), 7.78 (one half of AA'XX' spin system split into d through coupling with ^{13}C, see Figure S27a, 2 H, $H_{phenylene}$ *ortho* to C=O), 7.57 – 7.52 (m, 3 H, overlapping signals of H_{phenyl} *para* and *meta* to N), 7.22 – 7.19 (m, 2 H, overlapping signals of $H_{dimethylphenyl}$ *ortho* to N and $H_{dimethylphenyl}$ *ortho* to both methyl groups), 7.17 – 7.14 (m, 1 H, $H_{dimethylphenyl}$ *para* to Me), 4.17 (s, 2 H, $N_{pyrazole}$=$C_{pyrazole}CH_2S$), 4.03 (s, 2 H, $SCH_2C_{pyrazole}$=$C_{pyrazole}$), 2.40 (s, 3 H, CH_3 *para* to N), 2.12 (s, 3 H, CH_3 *ortho* to N). $^{13}C\{^1H\}$ NMR (151 MHz, CD_2Cl_2): δ [ppm] = 163.2 (C=O), 158.9 (C=N), 155.1 ($C_{phenylene}$-N), 152.9 (NC_{phenyl}), 140.7 ($Me\underline{C}_{dimethylphenyl}$ *para* to N), 136.6

(Me$\underline{C}_{dimethylphenyl}$ *ortho* to N), 135.0 (d, $^{1}J_{CC}$ = 66.8 Hz, O=C$\underline{C}_{phenylene}$), 134.4 ($\underline{C}_{dimethylphenyl}$N), 132.7 ($HC_{dimethylphenyl}$ *ortho* to both methyl groups), 132.3 (C_{phenyl} *para* to N), 130.7 ($C_{pyrazole}$N), 129.6 (C_{phenyl} *meta* to N), 128.6 (d, $^{2}J_{CC}$ = 2.5 Hz, $C_{phenylene}$ *ortho* to C=O), 128.1 ($HC_{dimethylphenyl}$ *para* to Me), 127.8 ($HC_{dimethylphenyl}$ *ortho* to N), 123.48 (C_{phenyl} *ortho* to N), 123.44 (d, $^{3}J_{CC}$ = 4.5 Hz, $C_{phenylene}$ *meta* to C=O), 114.2 ($\underline{C}_{pyrazole}$=$C_{pyrazole}$-NH), 29.7 and 29.6 ($\underline{C}H_2S$), 21.4 (H_3C *para* to N), 17.5 (H_3C *ortho* to N). Accurate MS (ESI): *m/z* calcd. for $[M + H]^+$ $C_{25}{}^{13}CH_{24}N_5{}^{18}OS^+$: 457.1772; found: 457.1765.

(*E*)-Azobenzene-4-[$^{18}O_2$]carboxylic acid (4b). Both experiments were performed following the reported protocol[4] with changes. In line with the protocol, immediately before the experiments, all reagents and starting materials were thoroughly dried by creating sonicated suspensions in anhydrous CH_3CN, followed by evaporation under reduced pressure to remove any water as an azeotrope with CH_3CN. This process was repeated three times before labeling. The reaction apparatus was dried under an argon flow with heating, and the reactions were carried out under argon using the Schlenk technique.

Experiment 1. 3,5-Dimethylpyridine hydrobromide (850 mg, 4.52 mmol) was suspended in DMF (2 mL). EDC•HCl (435 mg, 2.27 mmol) and $H_2{}^{18}O$ (225 µL, 12.5 mmol) were added. A suspension of (*E*)-azobenzene-4-carboxylic acid (**4a**; 51 mg, 225 µmol) in DMF (2 mL) was added immediately. The suspension was stirred at room temperature for 7 hours. HPLC-MS analysis of the reaction mixture indicated an ^{18}O-enrichment of 27%. A second portion of EDC•HCl (440 mg, 2.29 mmol) was added and the suspension was stirred for 17 hours. HPLC-MS analysis of the reaction mixture indicated an ^{18}O-enrichment of 37%. A third portion of dry EDC•HCl (444 mg, 2.32 mmol) was added and the suspension was stirred at room temperature for 22 hours. HPLC-MS analysis of the reaction mixture indicated an ^{18}O-enrichment of 42%. The reaction suspension was stirred at room temperature for additional 5 days and HPLC-MS analysis of the reaction mixture indicated an ^{18}O-enrichment of 43%.

Experiment 2. 3,5-Dimethylpyridine hydrobromide (850 mg, 4.52 mmol) were suspended in DMF (2 mL). A suspension of EDC•HCl (430 mg, 2.24 mmol) in DMF (1 mL) and $H_2{}^{18}O$ (225 µL, 12.5 mmol) were added. A suspension of (*E*)-azobenzene-4-carboxylic acid (**4a**; 51 mg, 225 µmol) in DMF (2 mL) was added immediately. The suspension was stirred at room temperature for 16 hours. HPLC-MS analysis of the reaction mixture indicated an ^{18}O-enrichment of 9%. A second portion of EDC•HCl (460 mg, 2.40 mmol) was added and the suspension was stirred for 5 hours. HPLC-MS analysis of the reaction mixture indicated an ^{18}O-enrichment of 27%. A third portion of dry EDC•HCl (470 mg, 2.45 mmol) was added and the suspension was stirred at room temperature for 20 hours. HPLC-MS analysis of the reaction mixture indicated an ^{18}O-enrichment of 36%. A fourth portion of dry EDC•HCl (360 mg, 1.88 mmol) was added and the suspension was stirred at 40 °C for 19 hours. Shortly after the bath had reached 40 °C, the orange suspension turned into an orange solution. HPLC-MS analysis of the reaction mixture indicated an ^{18}O-enrichment of 36%. $H_2{}^{18}O$ (100 µL, 5.55 mmol) was added and the reaction solution was stirred at 40 °C for 3 days. HPLC-MS analysis of the reaction mixture indicated an ^{18}O-enrichment of 48%.

MS Spectra

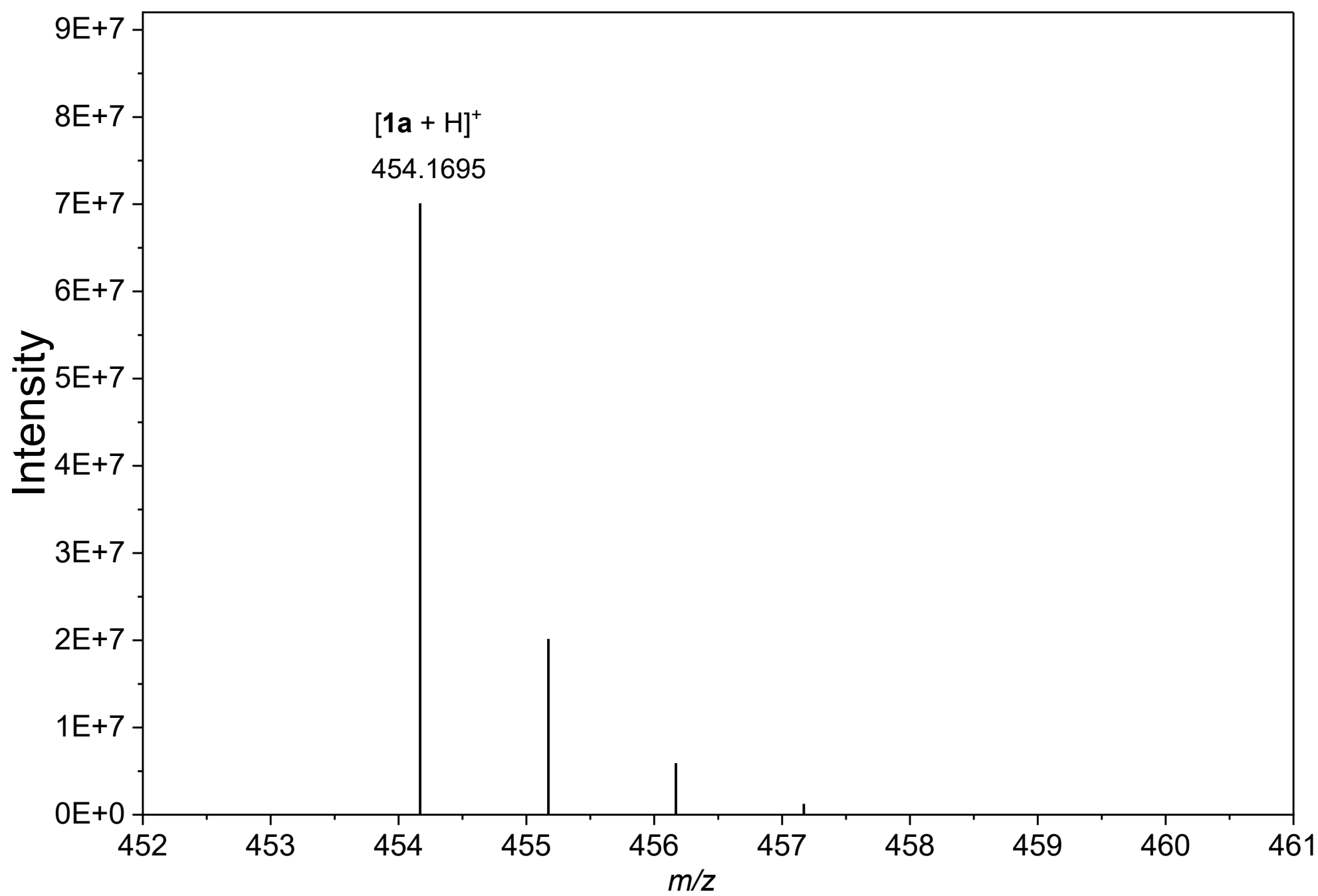

Figure S14. Accurate MS spectrum (ESI, positive mode) of photopharmacological compound **1a**.

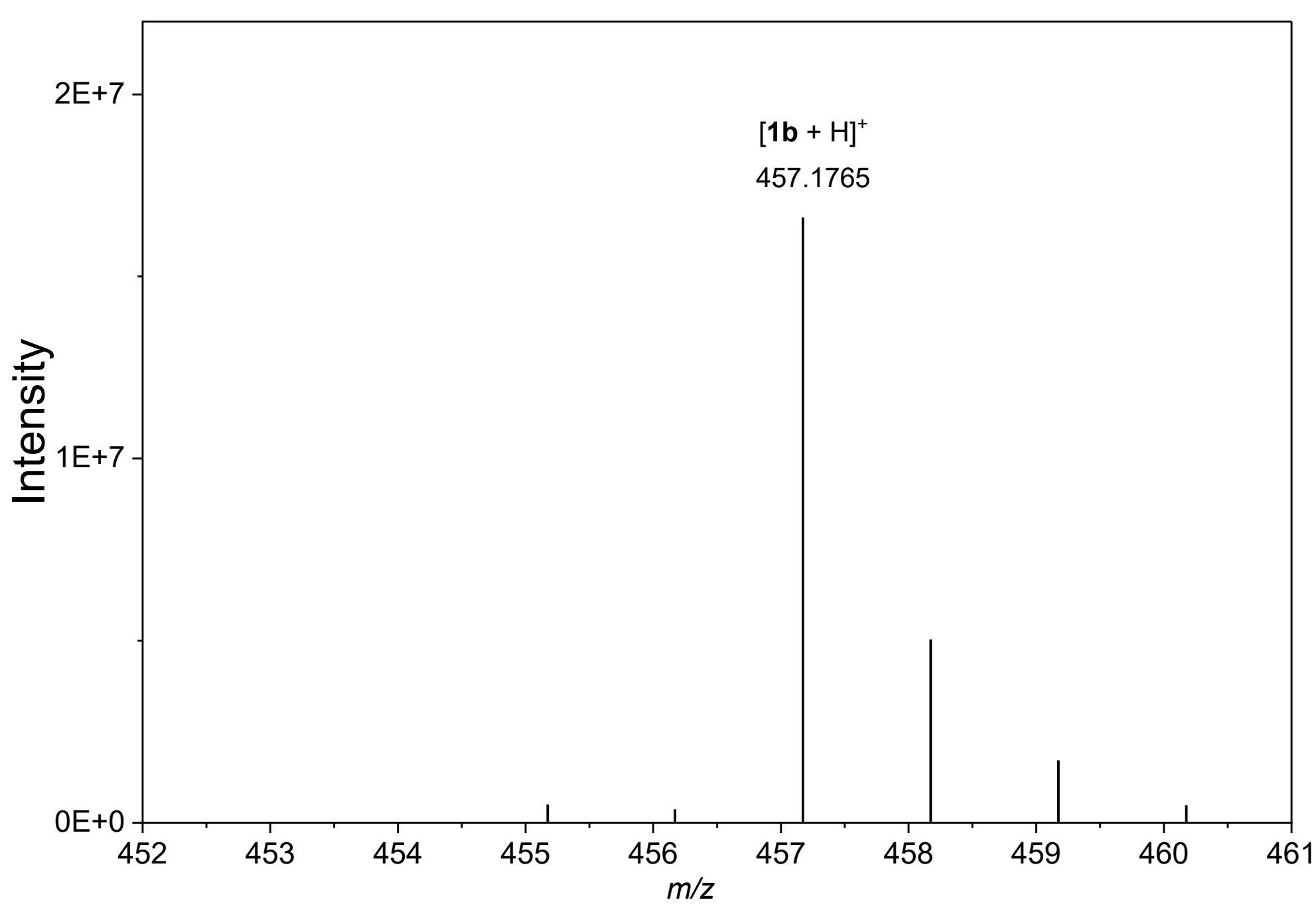

Figure S15. Accurate MS spectrum (ESI, positive mode) of photopharmacological compound **1b**.

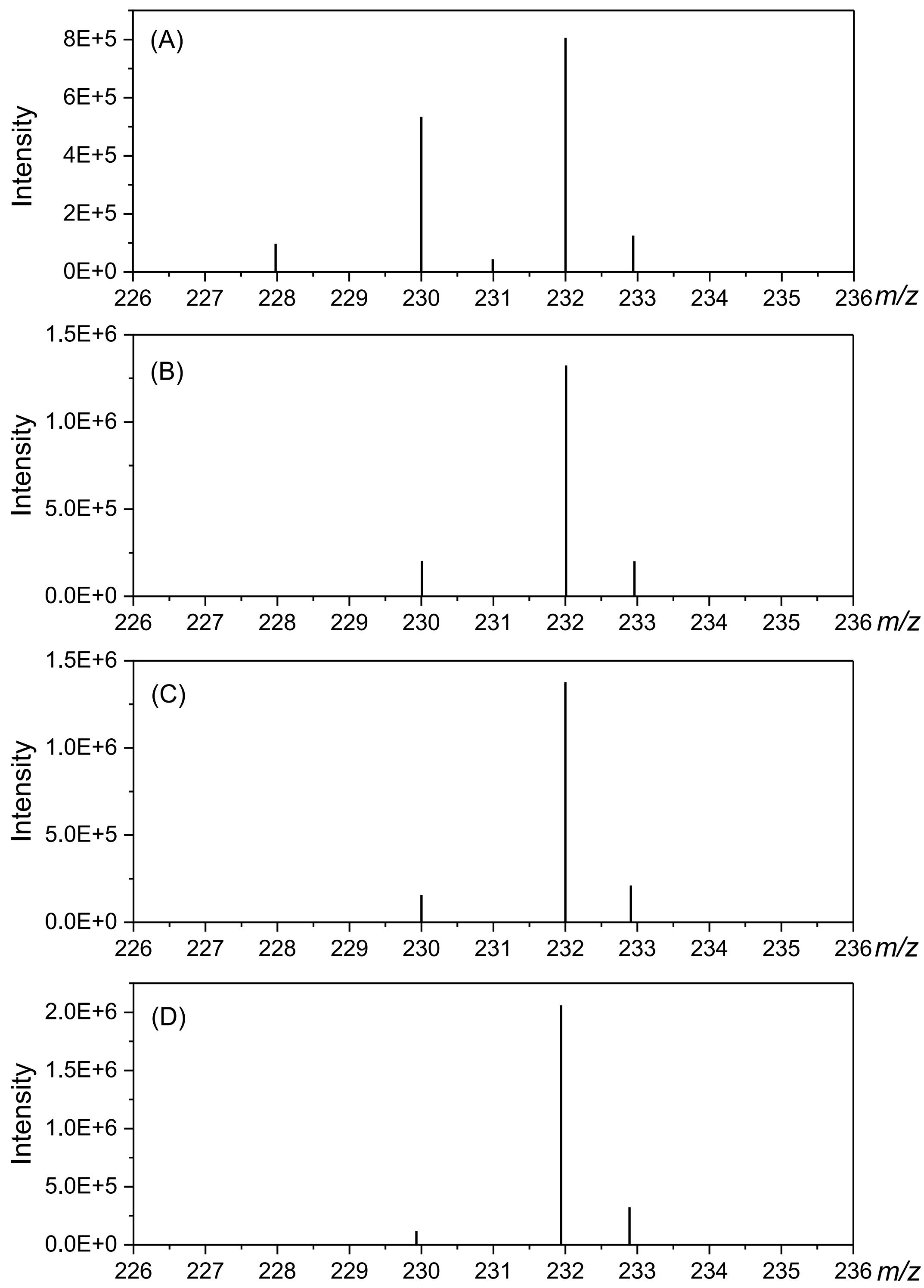

Figure S16. The MS spectra (DUIS, positive mode) from the HPLC-MS analyses of the ^{18}O-labeling of (*E*)-azobenzene-4-[^{13}C]carboxylic acid (**4c**) with four sequential treatments with $H_2{}^{18}O$ and HCl in dioxane. (A)-(D) show the spectra after each treatment, revealing ^{18}O-enrichments of 75%, 93%, 95%, and 97%, respectively.

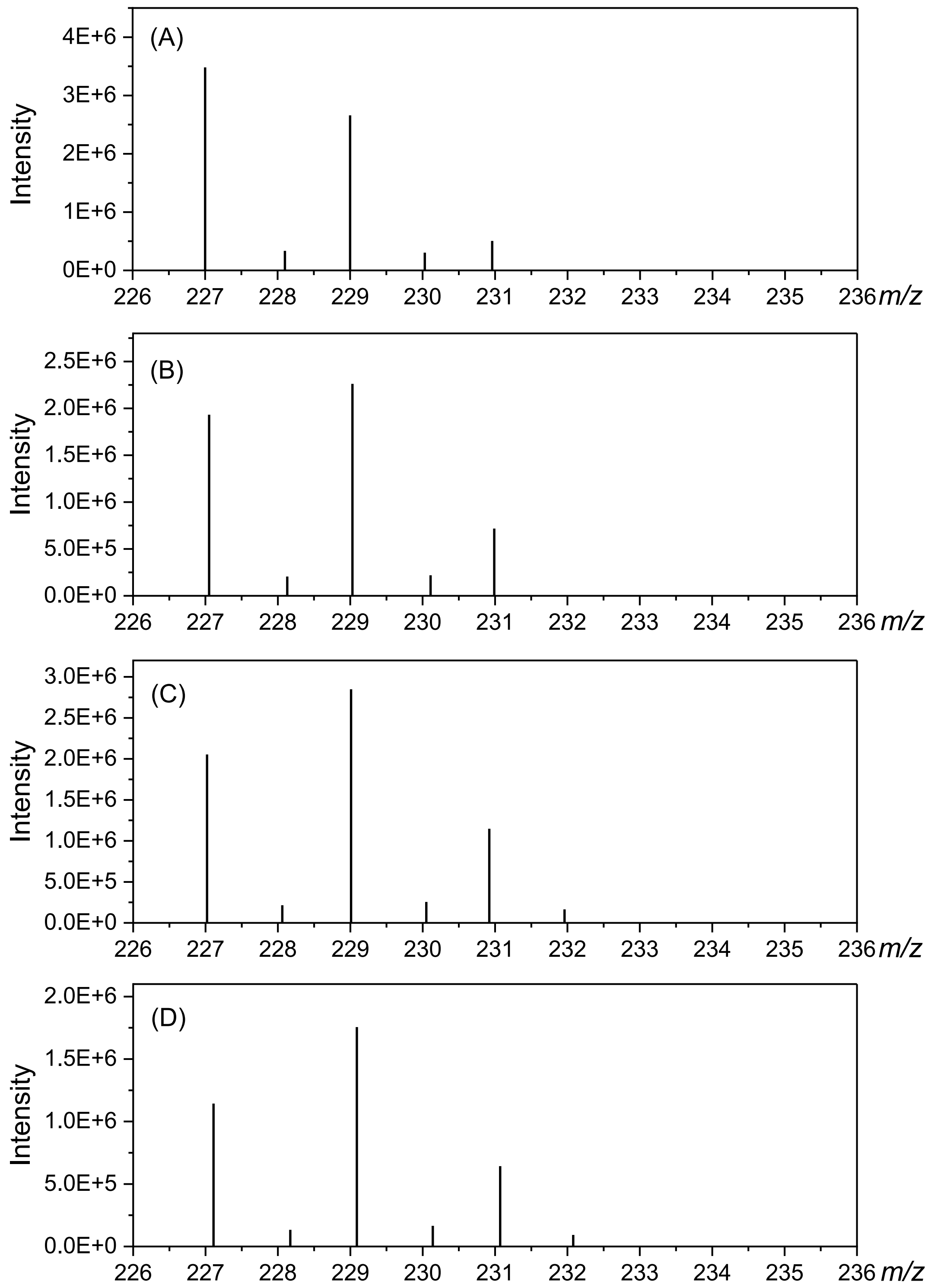

Figure S17. The MS spectra (DUIS, positive mode) from the HPLC-MS analysis of the products of the first attempt of ^{18}O-labeling of (*E*)-4-azobenzene-4-carboxylic acid (**4a**) using $H_2{}^{18}O$ and EDC•HCl in DMF. (A)-(C) present the spectra after each addition of EDC•HCl and (D) shows the spectrum after an additional reaction time of 5 days, revealing ^{18}O-enrichments of 27%, 37%, 42%, and 43%, respectively.

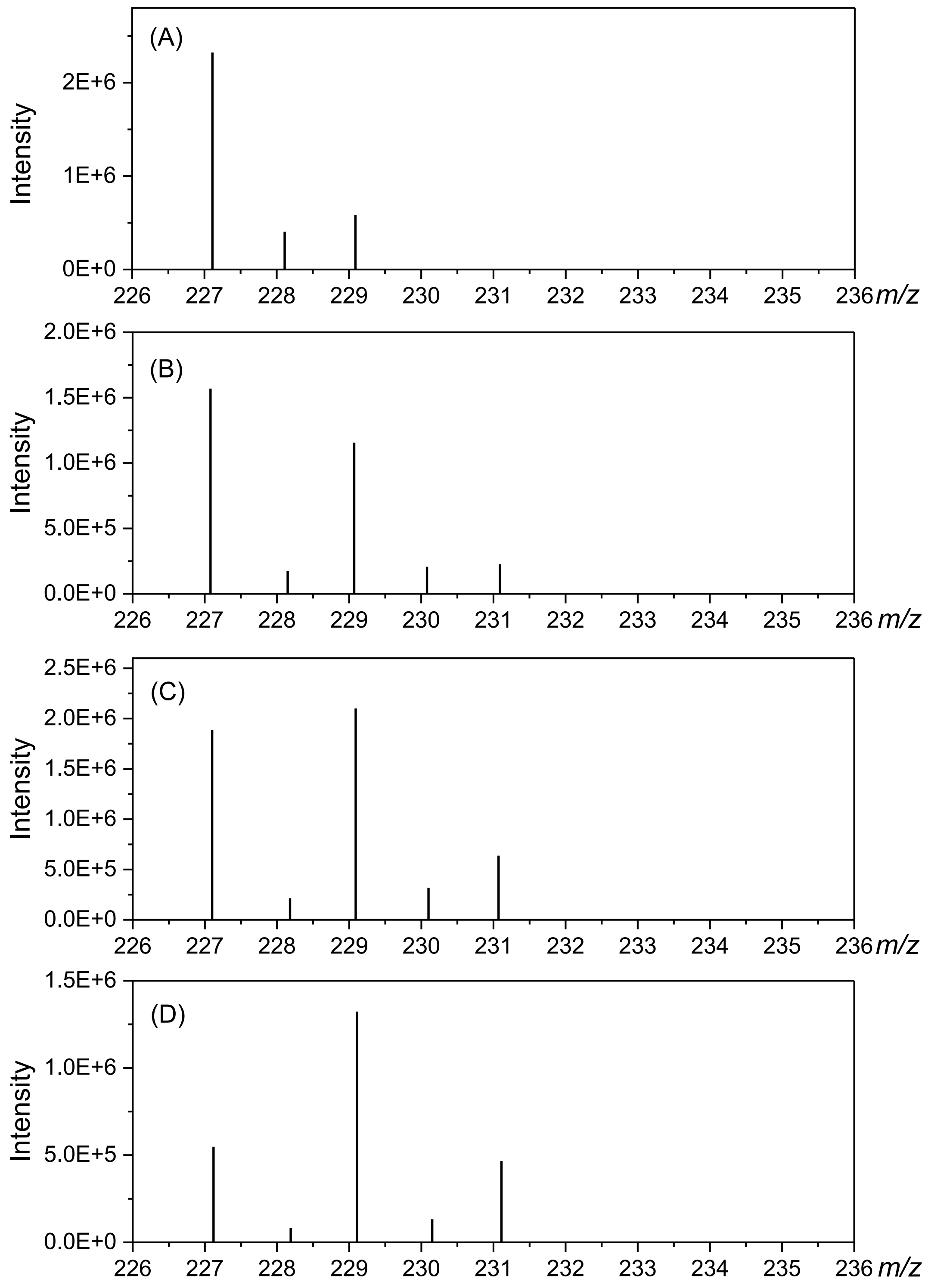

Figure S18. The MS spectra (DUIS, positive mode) from the HPLC-MS analysis of the products of the second attempt of ^{18}O-labeling of (*E*)-4-azobenzene-4-carboxylic acid (**4a**) using $H_2{}^{18}O$ and EDC•HCl in DMF. (A)-(C) present the spectra after each addition of EDC•HCl, and (D) shows the spectrum after an additional reaction time of 3 days at 40 °C, revealing ^{18}O-enrichments of 9%, 27%, 36%, and 48%, respectively.

NMR Spectra

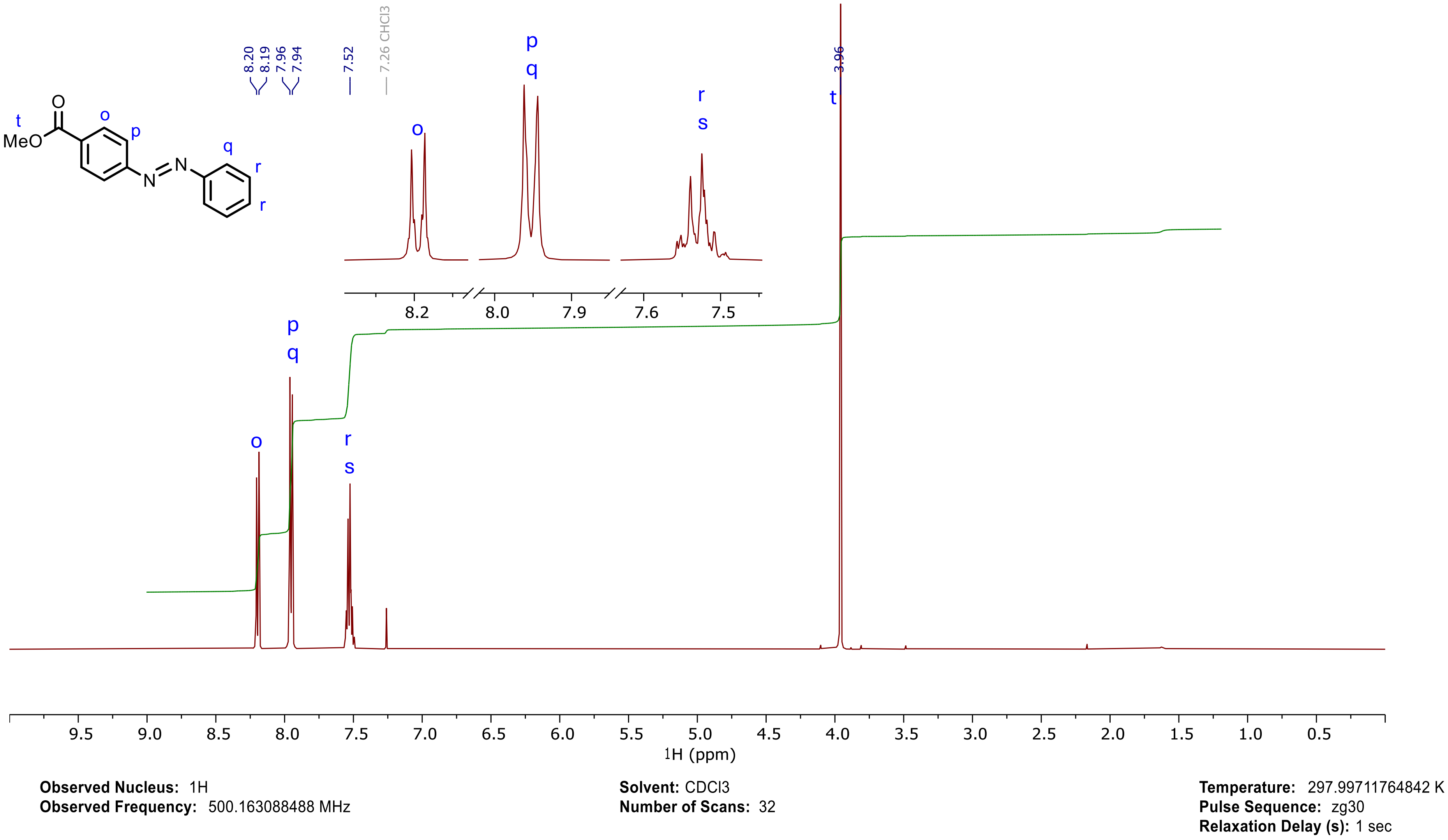

Figure S19a. ^{1}H NMR spectrum (500 MHz, $CDCl_3$) of methyl (*E*)-azobenzene-4-carboxylate (**3a**).

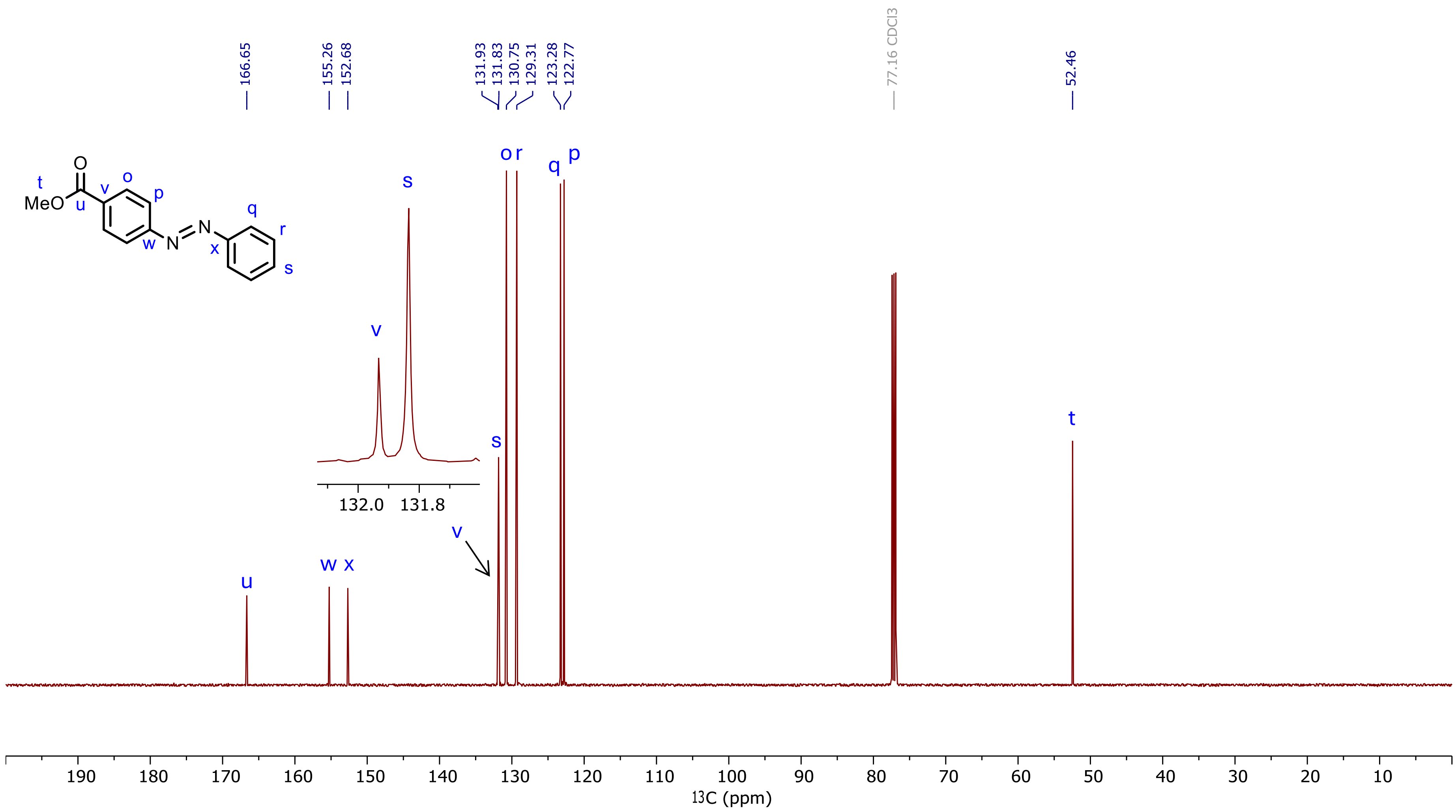

Observed Nucleus: 13C
Observed Frequency: 125.777908565 MHz

Solvent: CDCl3
Number of Scans: 1024

Temperature: 297.999920052533 K
Pulse Sequence: zgpg30
Relaxation Delay (s): 2 sec

Figure S19b. $^{13}C\{^{1}H\}$ NMR spectrum (126 MHz, $CDCl_3$) of methyl (*E*)-azobenzene-4-carboxylate (**3a**).

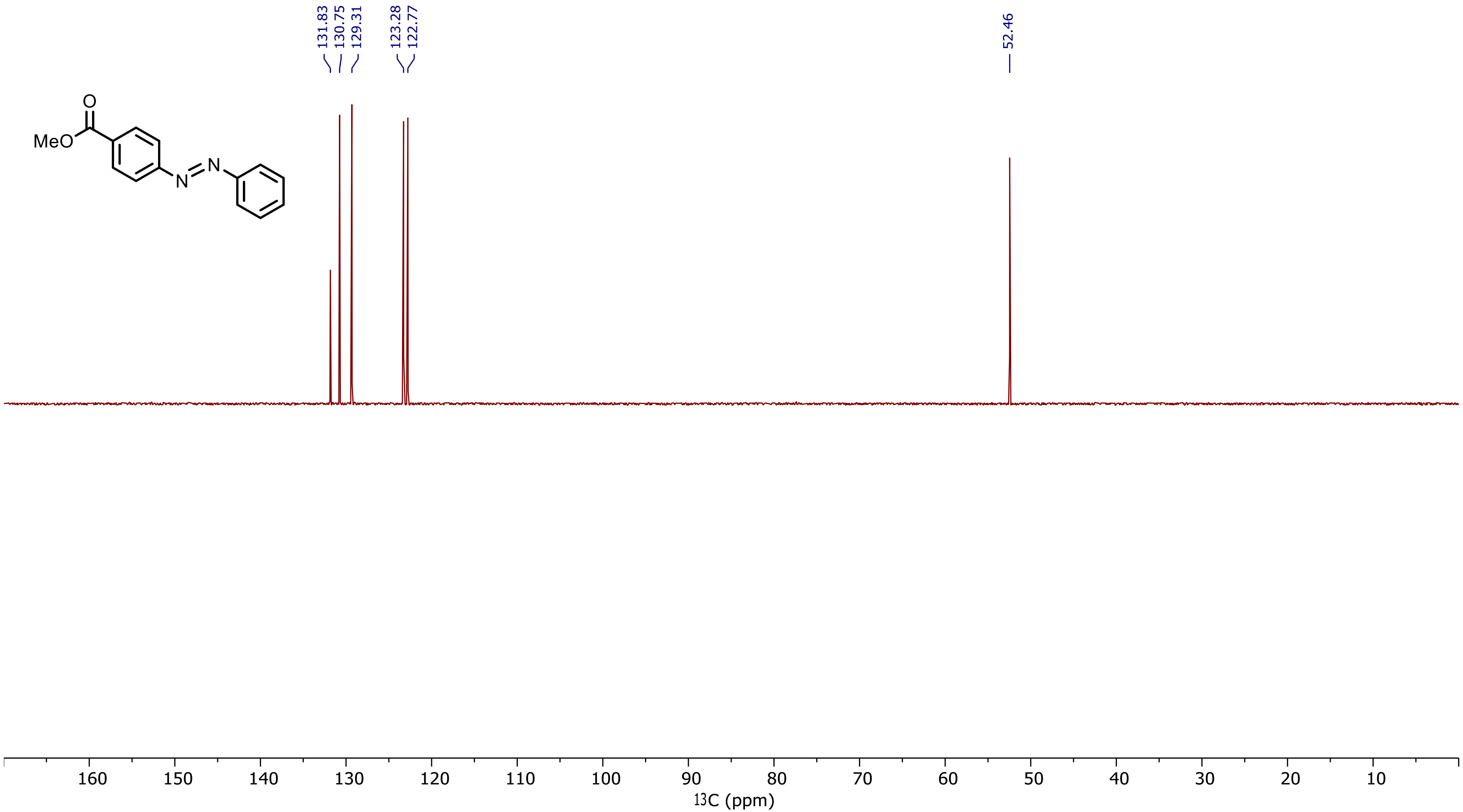

Observed Nucleus: 13C
Observed Frequency: 125.775393258 MHz

Solvent: CDCl3
Number of Scans: 256

Temperature: 298.00218005585 K
Pulse Sequence: deptsp135
Relaxation Delay (s):2 sec

Figure S19c. ^{13}C DEPT 135 NMR spectrum (126 MHz, $CDCl_3$) of methyl (*E*)-azobenzene-4-carboxylate (**3a**).

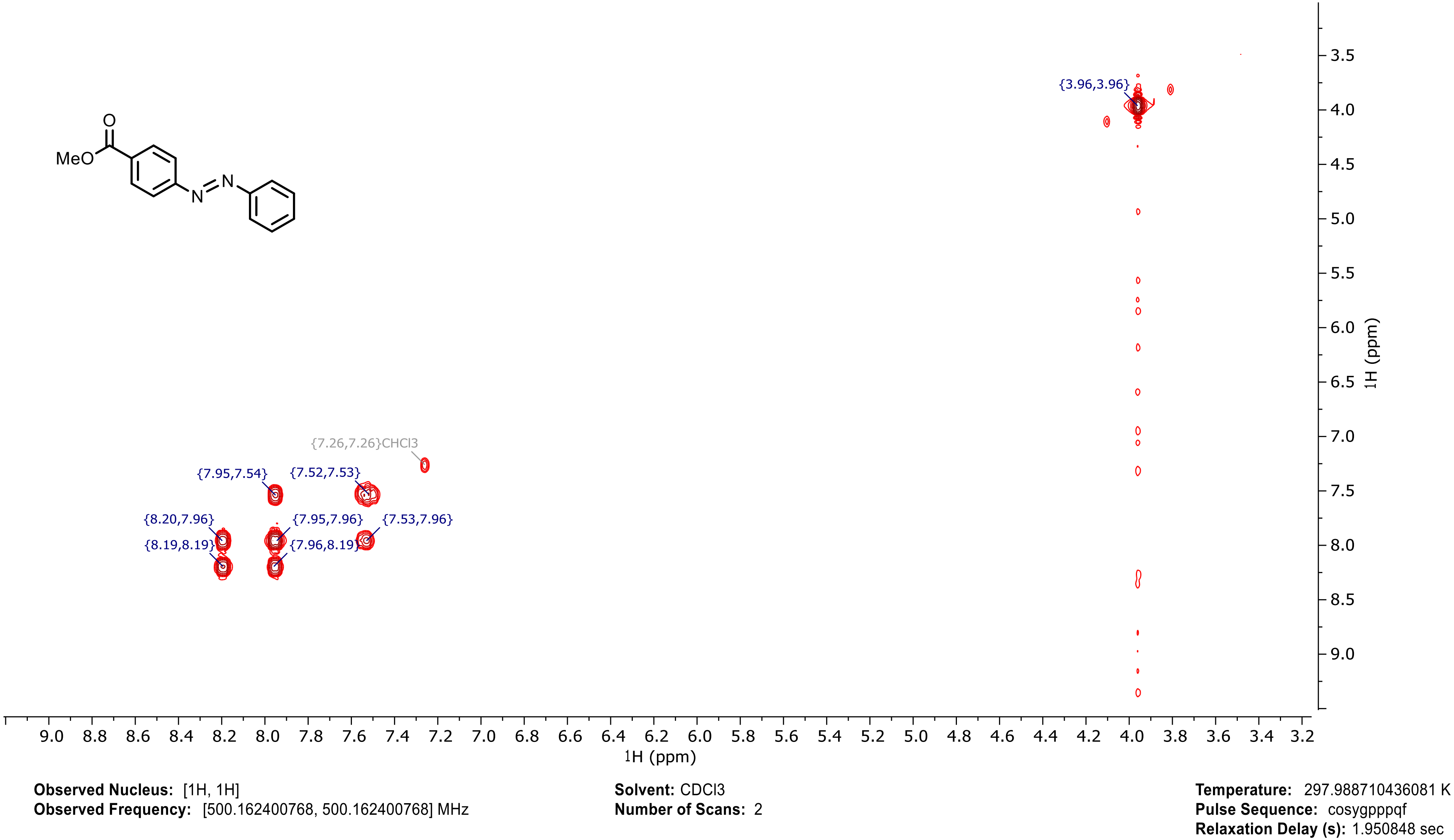

Figure S19d. ^{1}H ^{1}H COSY NMR spectrum (500 MHz, 500 MHz, $CDCl_3$) of methyl (*E*)-azobenzene-4-carboxylate (**3a**).

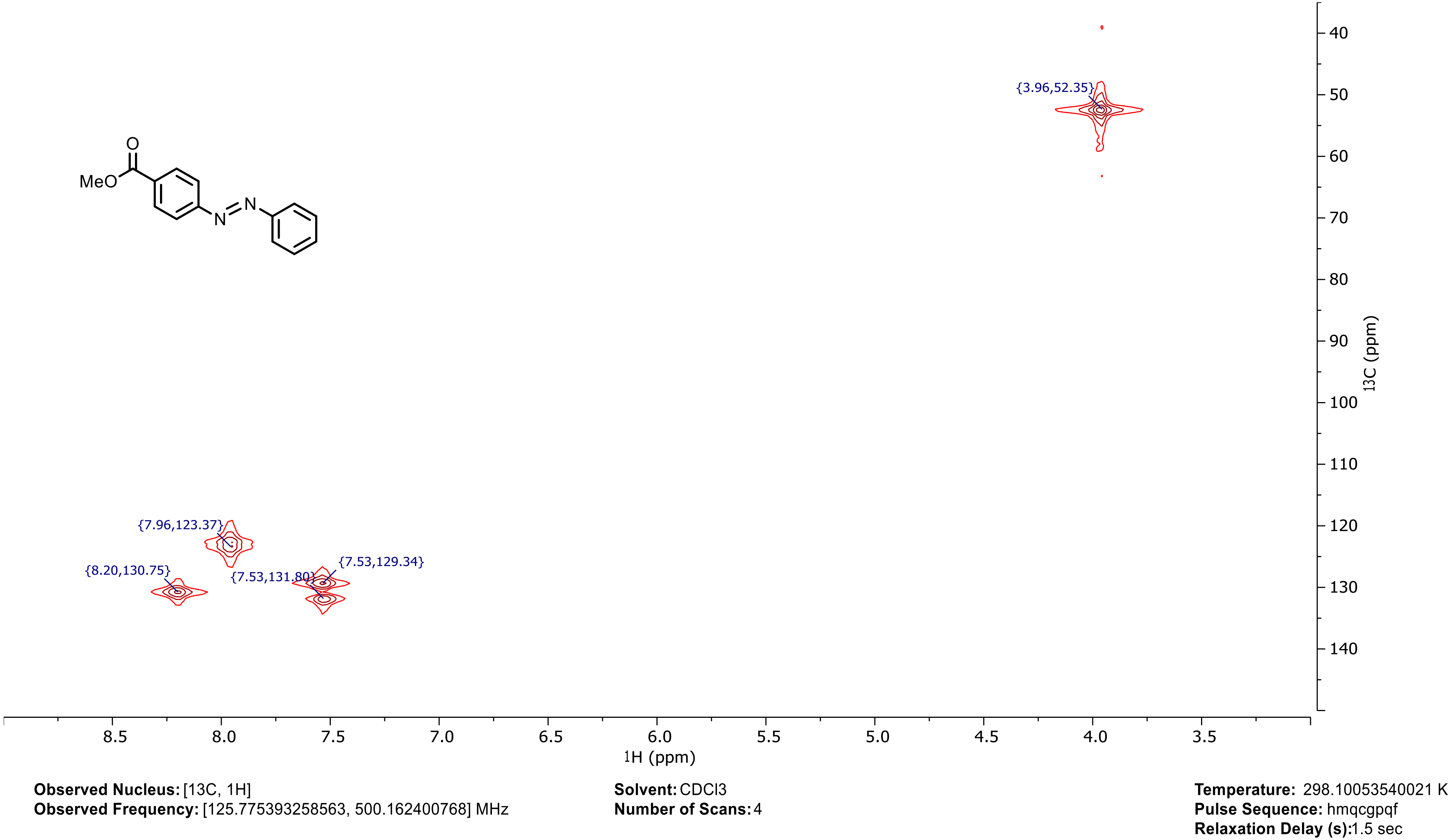

Figure S19e. ^{13}C ^{1}H HMQC NMR spectrum (126 MHz, 500 MHz, $CDCl_3$) of methyl (*E*)-azobenzene-4-carboxylate (**3a**).

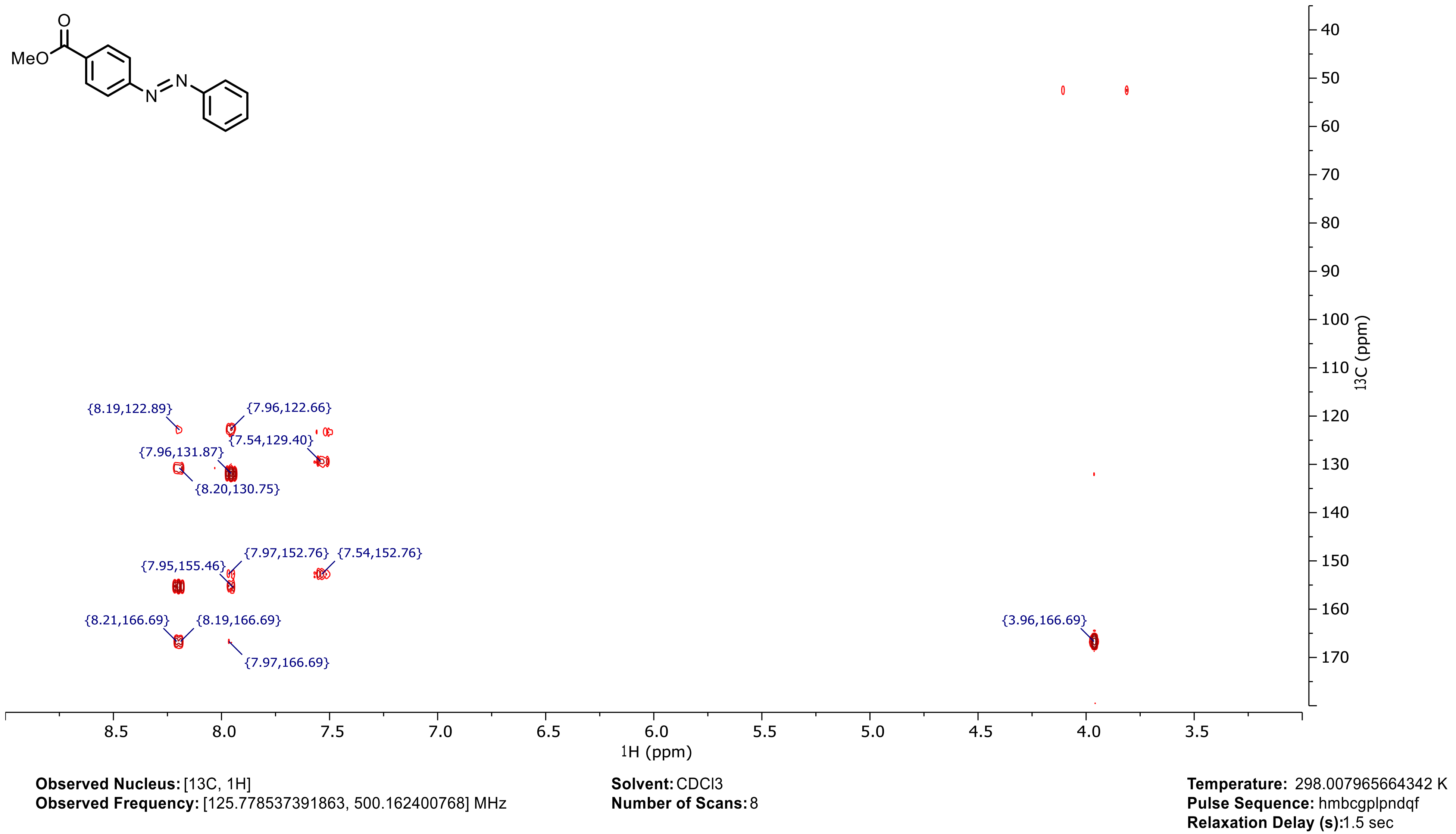

Figure S19f. ^{13}C ^{1}H HMBC NMR spectrum (126 MHz, 500 MHz, $CDCl_3$) of methyl (*E*)-azobenzene-4-carboxylate (**3a**).

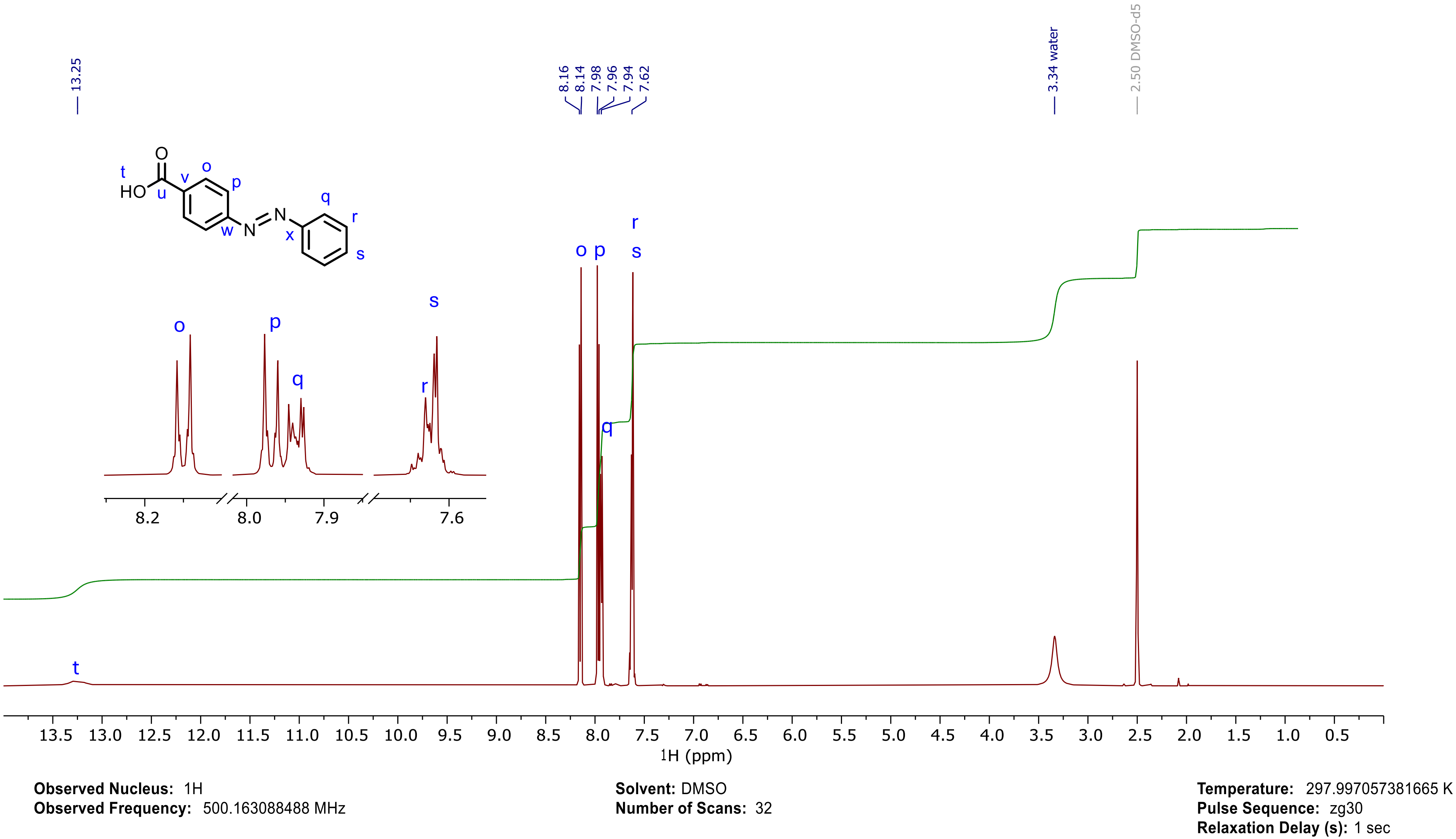

Figure S20a. ^{1}H NMR spectrum (500 MHz, DMSO-d6) of (*E*)-4-azobenzene-4-carboxylic acid (**4a**).

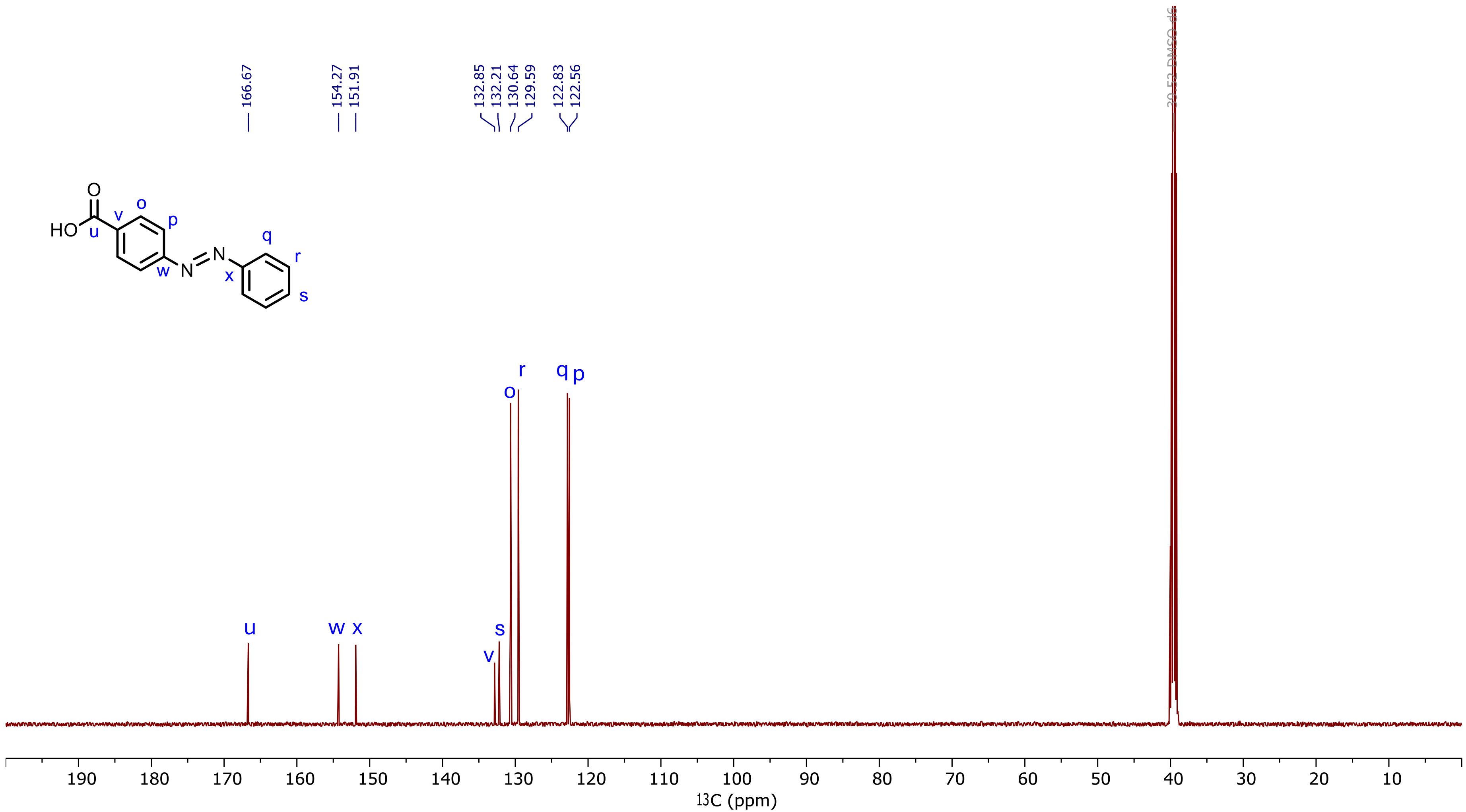

Observed Nucleus: 13C
Observed Frequency: 125.777908565 MHz

Solvent: DMSO
Number of Scans: 1024

Temperature: 298.003355257575 K
Pulse Sequence: zgpg30
Relaxation Delay (s): 2 sec

Figure S20b. $^{13}C\{^{1}H\}$ NMR spectrum (126 MHz, DMSO-d6) of (*E*)-4-azobenzene-4-carboxylic acid (**4a**).

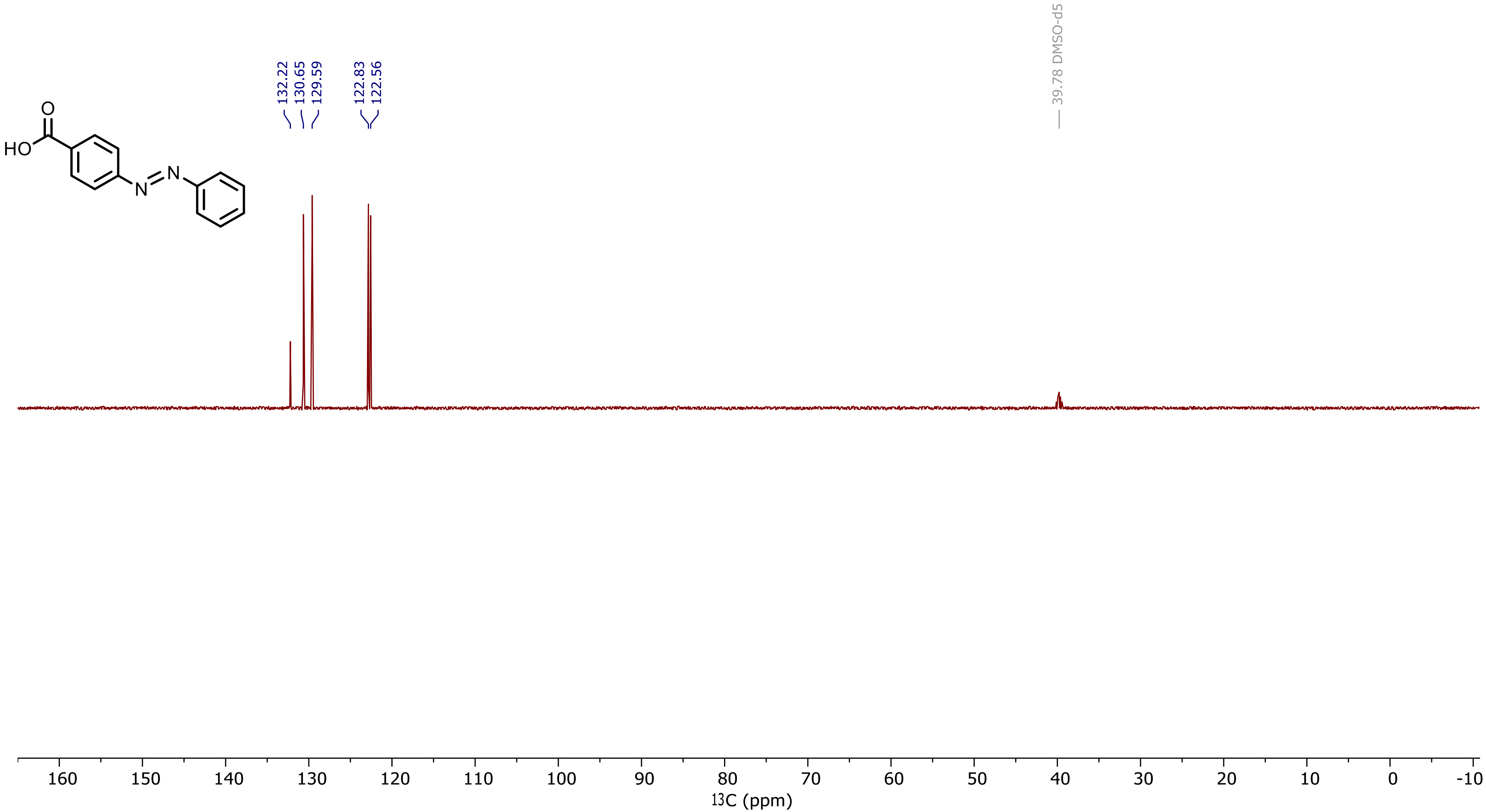

Observed Nucleus: 13C
Observed Frequency: 125.775393258 MHz
Solvent: DMSO
Number of Scans: 256
Temperature: 297.995821913185 K
Pulse Sequence: deptsp135
Relaxation Delay (s): 2 sec

Figure S20c. ^{13}C DEPT 135 NMR spectrum (126 MHz, DMSO-d6) of (*E*)-4-azobenzene-4-carboxylic acid (**4a**).

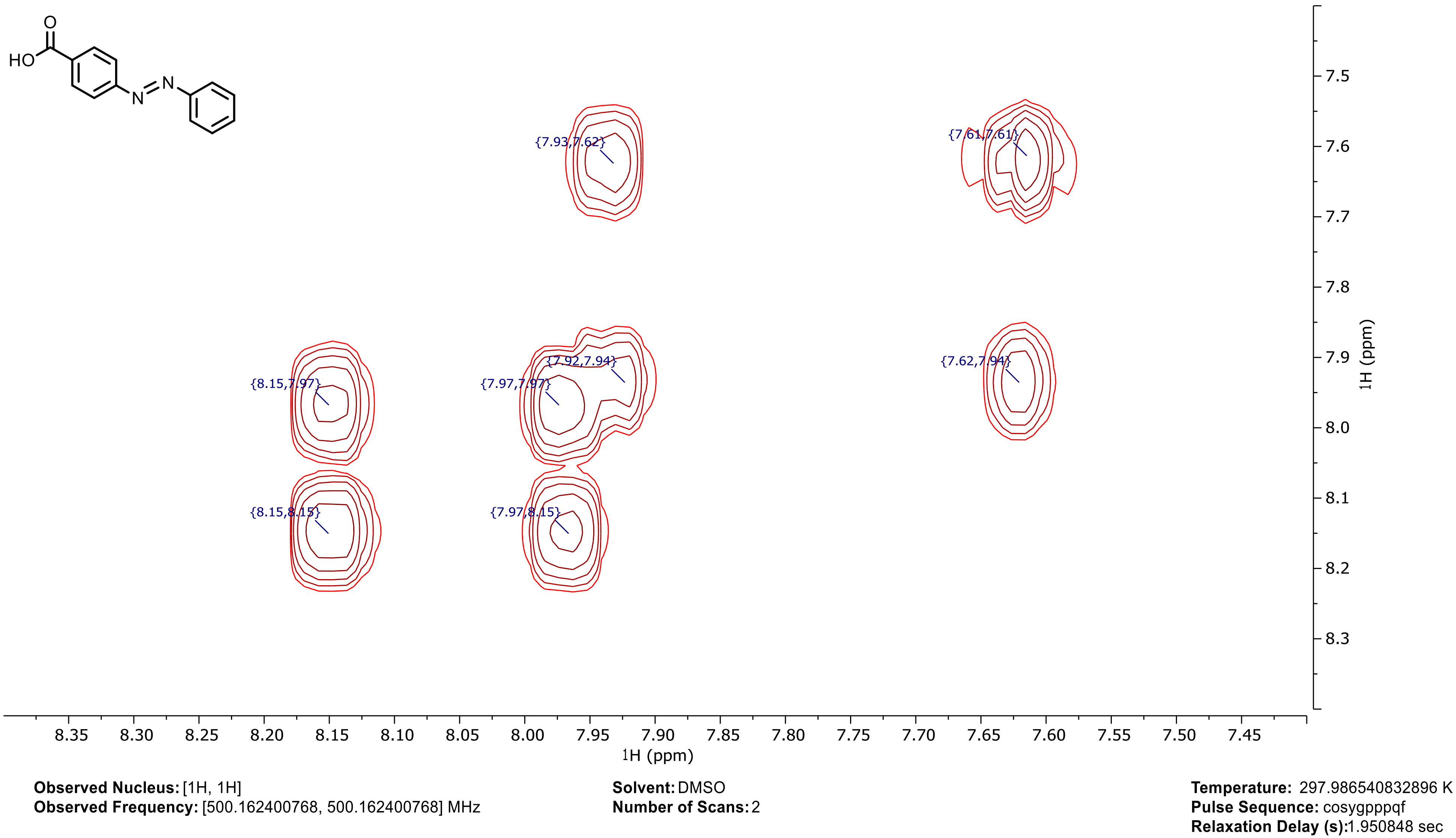

Figure S20d. ^{1}H ^{1}H COSY NMR spectrum (500 MHz, 500 MHz, DMSO-d6) of (*E*)-4-azobenzene-4-carboxylic acid (**4a**).

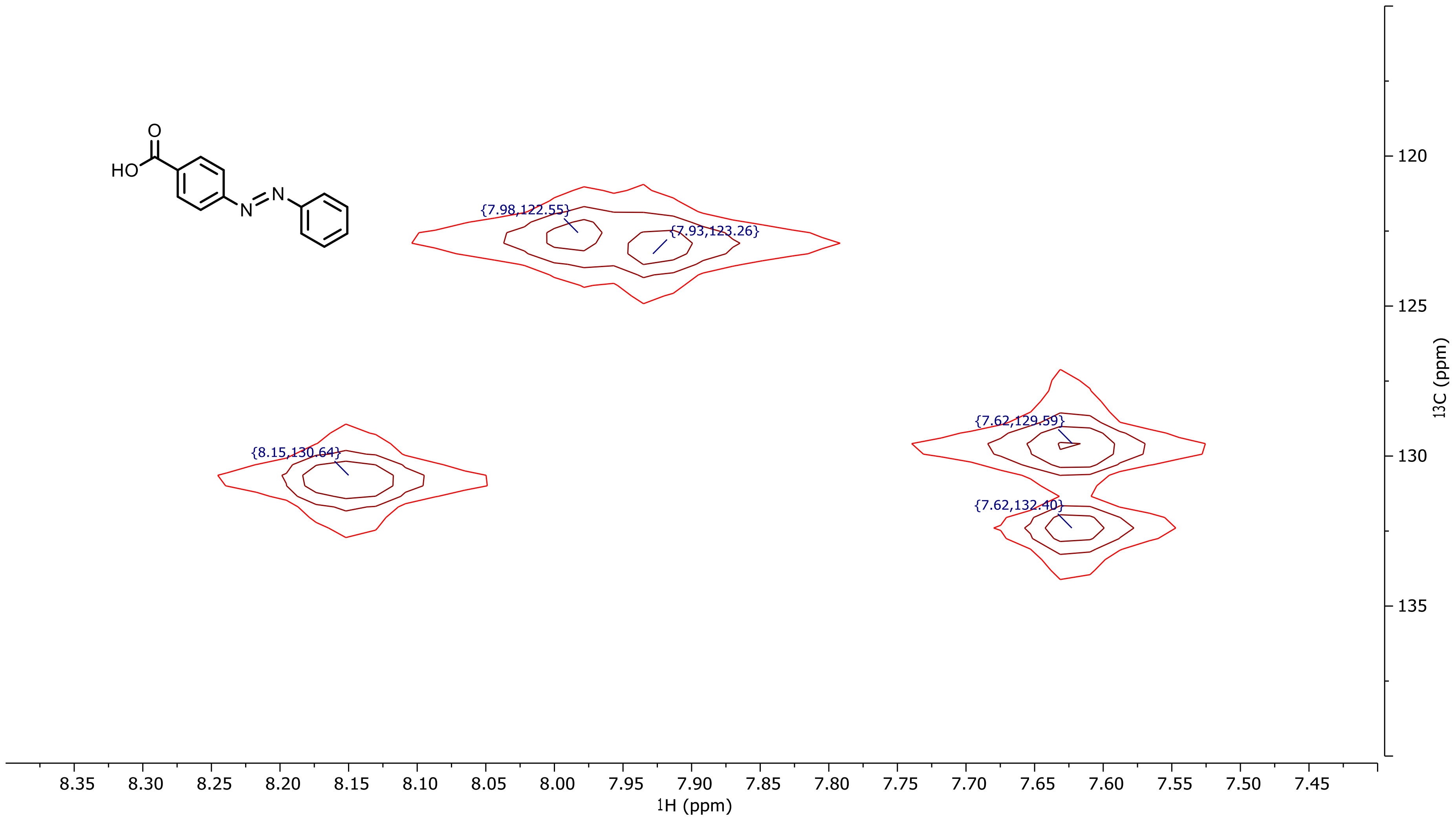

Observed Nucleus: [13C, 1H]
Observed Frequency: [125.775393258563, 500.162400768] MHz

Solvent: DMSO
Number of Scans: 4

Temperature: 298.09336365635 K
Pulse Sequence: hmqcgpqf
Relaxation Delay (s): 1.5 sec

Figure S20e. ^{13}C ^{1}H HMQC NMR spectrum (126 MHz, 500 MHz, DMSO-d6) of (*E*)-4-azobenzene-4-carboxylic acid (**4a**).

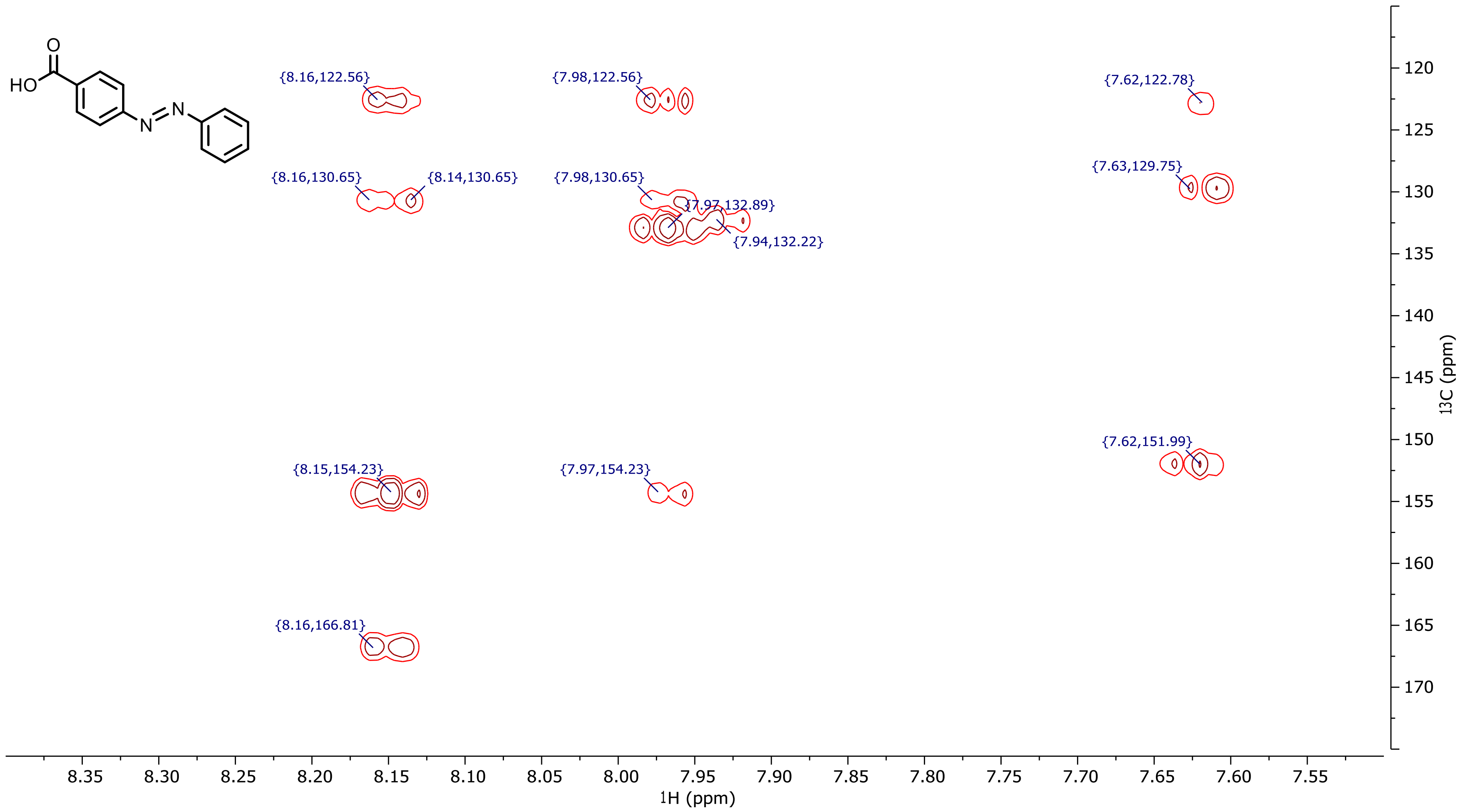

Observed Nucleus: [13C, 1H]
Observed Frequency: [125.778537391863, 500.162400768] MHz

Solvent: DMSO
Number of Scans: 8

Temperature: 297.996183513716 K
Pulse Sequence: hmbcgplpndqf
Relaxation Delay (s): 1.5 sec

Figure S20f. ^{13}C ^{1}H HMBC NMR spectrum (126 MHz, 500 MHz, DMSO-d6) of (*E*)-4-azobenzene-4-carboxylic acid (**4a**).

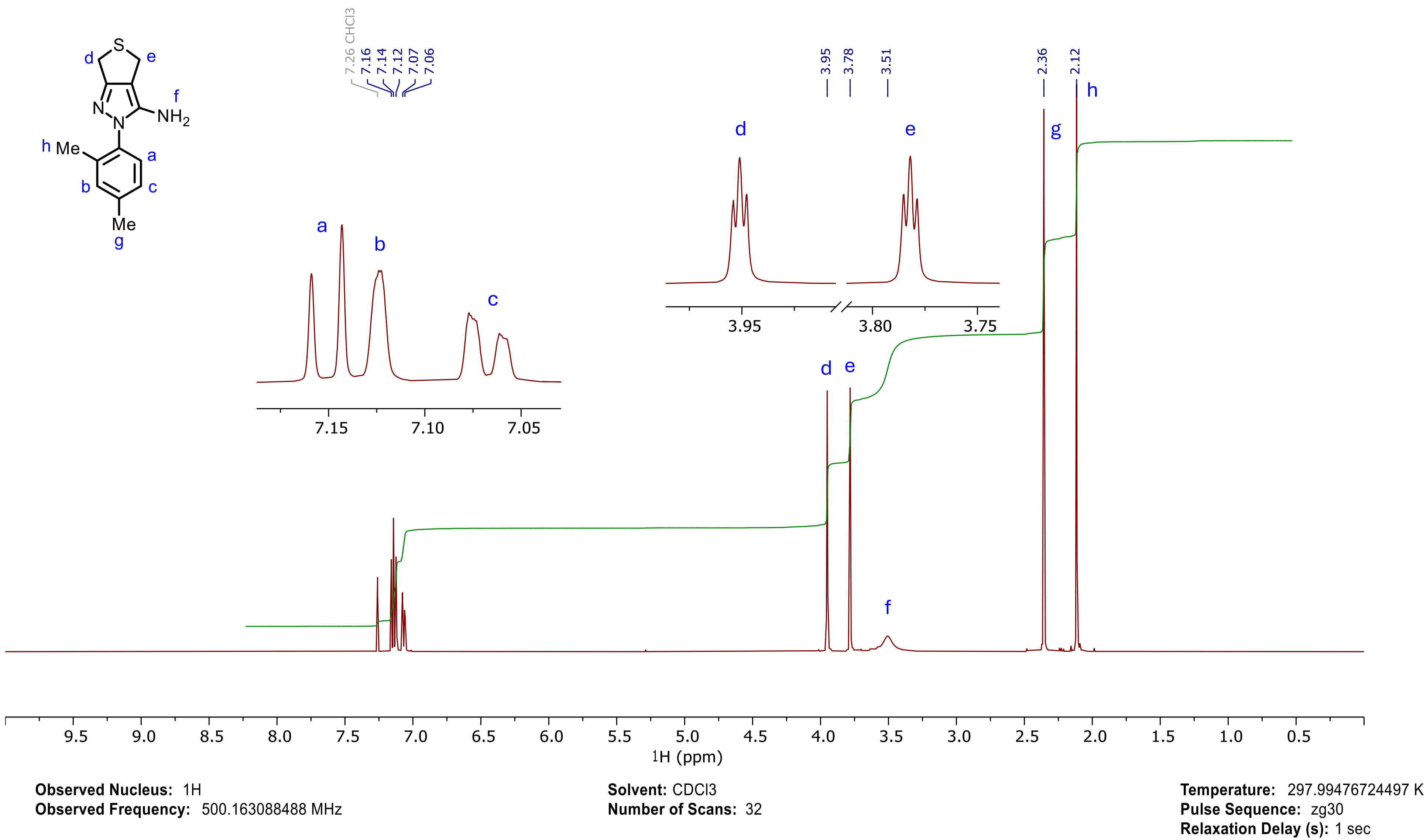

Figure S21a. ^{1}H NMR spectrum (500 MHz, $CDCl_3$) of pyrazolamine **5**.

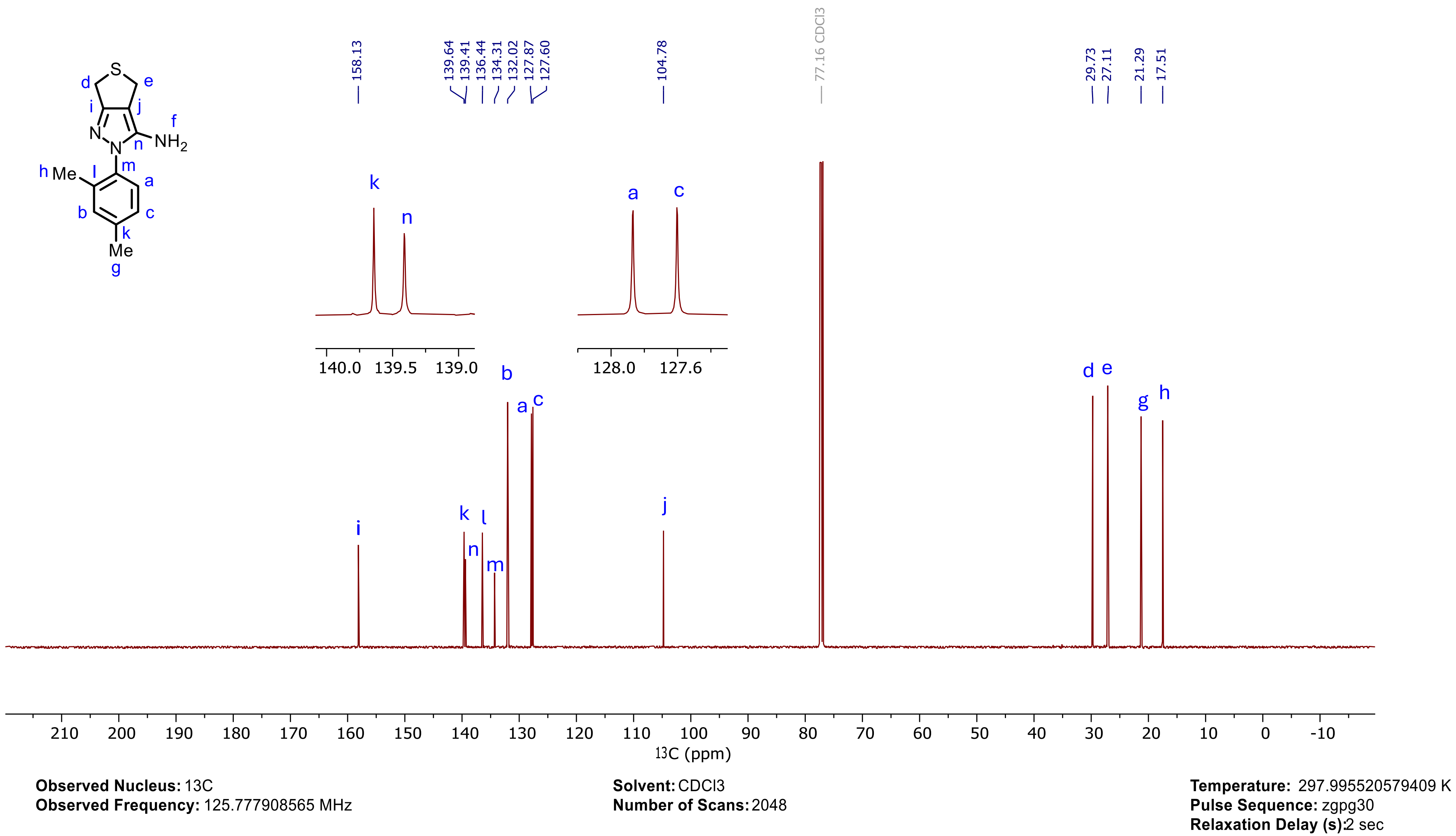

Figure S21b. $^{13}C\{^{1}H\}$ NMR spectrum (126 MHz, $CDCl_3$) of pyrazolamine **5**.

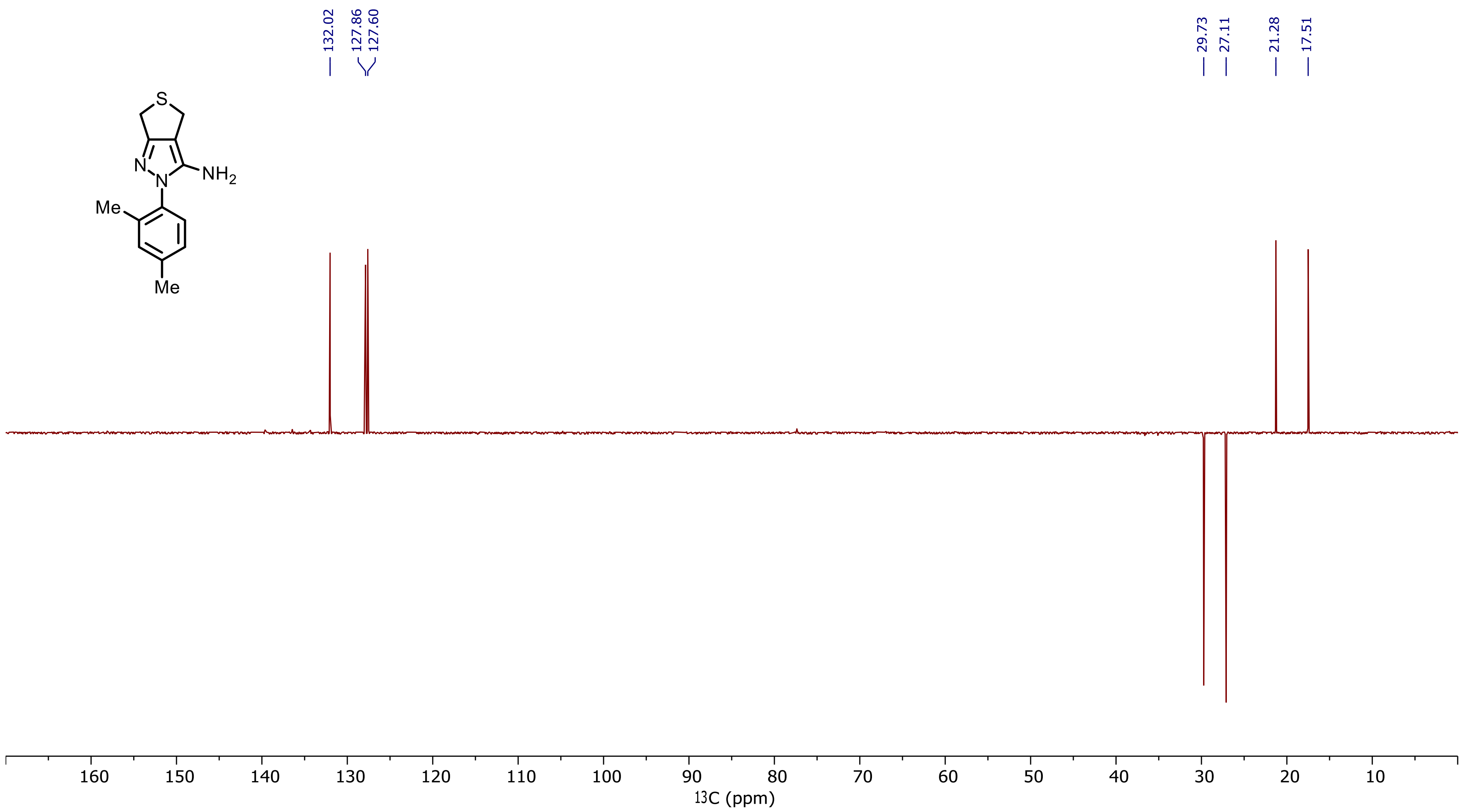

Observed Nucleus: 13C
Observed Frequency: 125.775393258 MHz

Solvent: CDCl3
Number of Scans: 512

Temperature: 297.987926968264 K
Pulse Sequence: deptsp135
Relaxation Delay (s): 2 sec

Figure S21c. ^{13}C DEPT 135 NMR spectrum (126 MHz, $CDCl_3$) of pyrazolamine **5**.

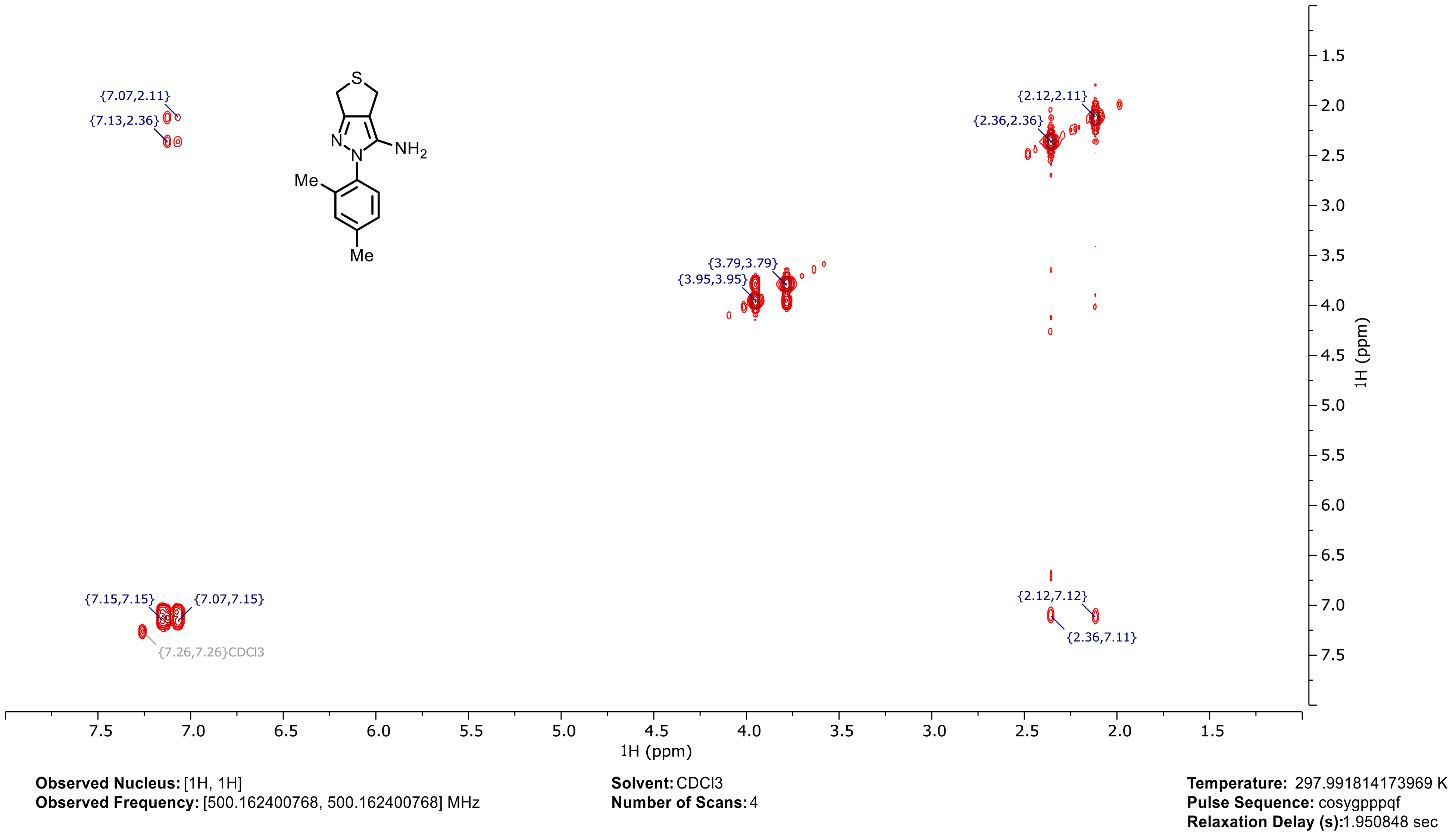

Figure S21d. ^{1}H ^{1}H COSY NMR spectrum (500 MHz, 500 MHz, $CDCl_3$) of pyrazolamine **5**.

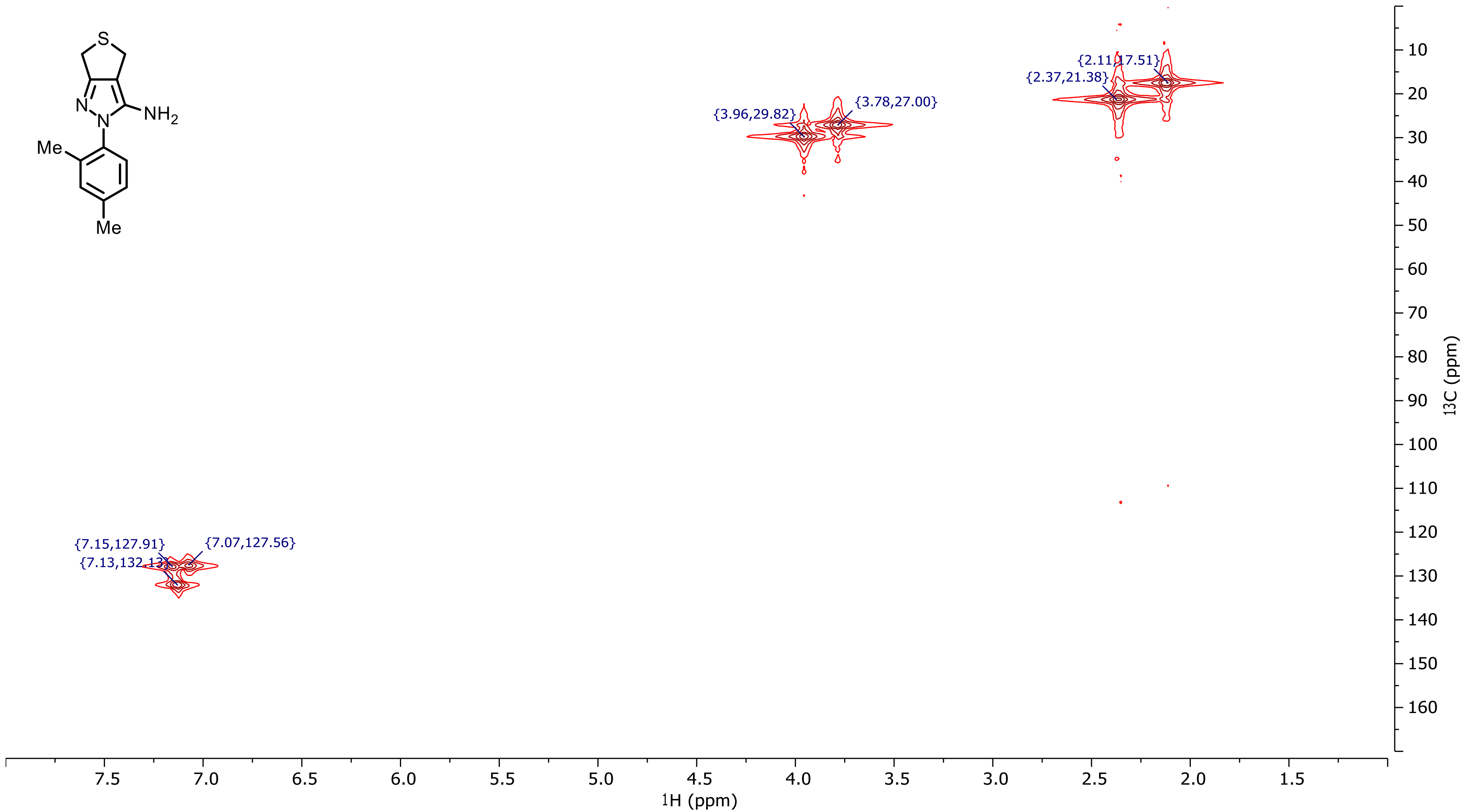

Observed Nucleus: [13C, 1H]
Observed Frequency: [125.775393258563, 500.162400768] MHz

Solvent: CDCl3
Number of Scans: 4

Temperature: 298.095322325892 K
Pulse Sequence: hmqcgpqf
Relaxation Delay (s): 1.5 sec

Figure S21e. ^{13}C ^{1}H HMQC NMR spectrum (126 MHz, 500 MHz, $CDCl_3$) of pyrazolamine **5**.

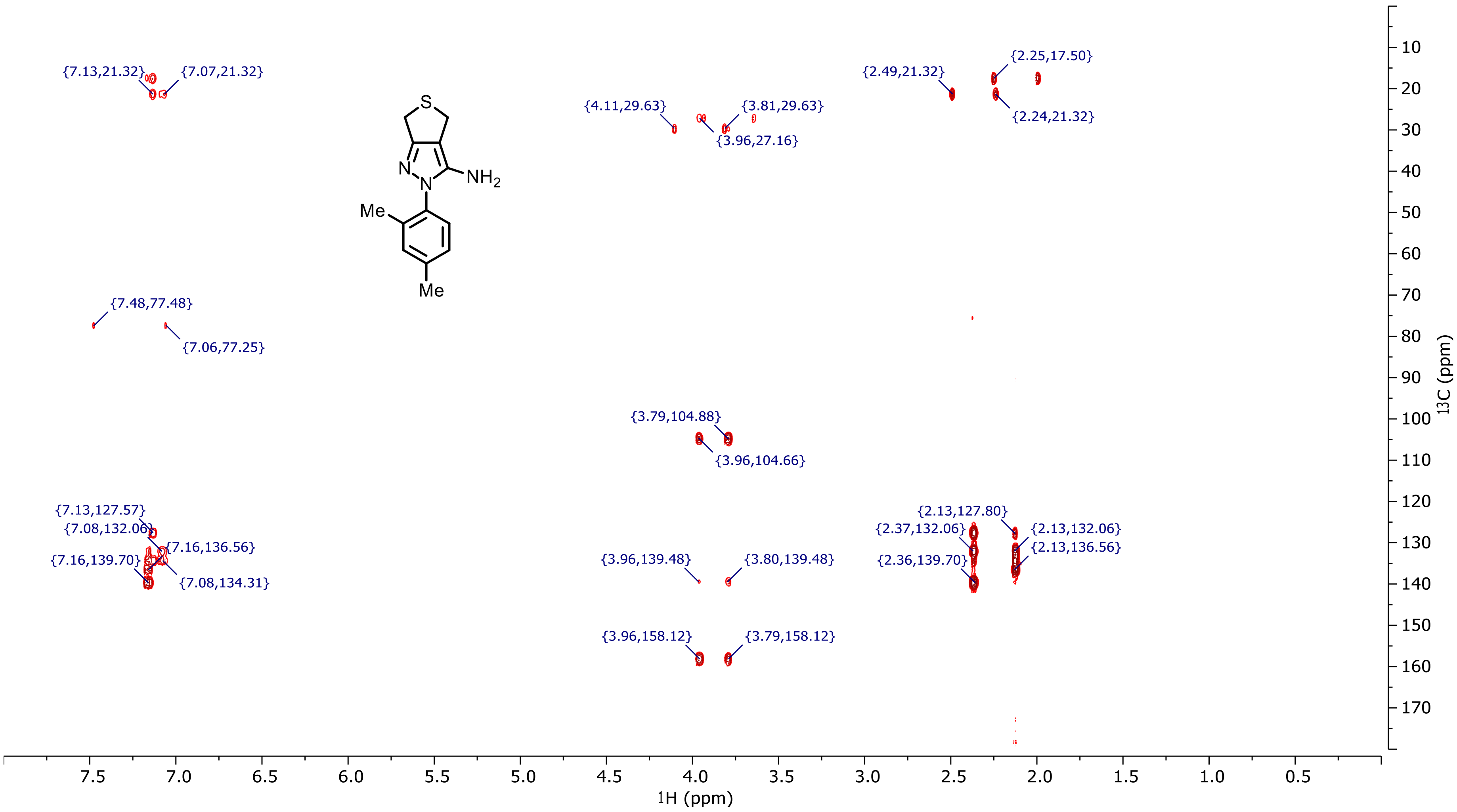

Observed Nucleus: [13C, 1H]
Observed Frequency: [125.778537391863, 500.162400768] MHz

Solvent: CDCl3
Number of Scans: 16

Temperature: 298.005404327249 K
Pulse Sequence: hmbcgplpndqf
Relaxation Delay (s): 1.5 sec

Figure S21f. ^{13}C ^{1}H HMBC NMR spectrum (126 MHz, 500 MHz, $CDCl_3$) of pyrazolamine **5**.

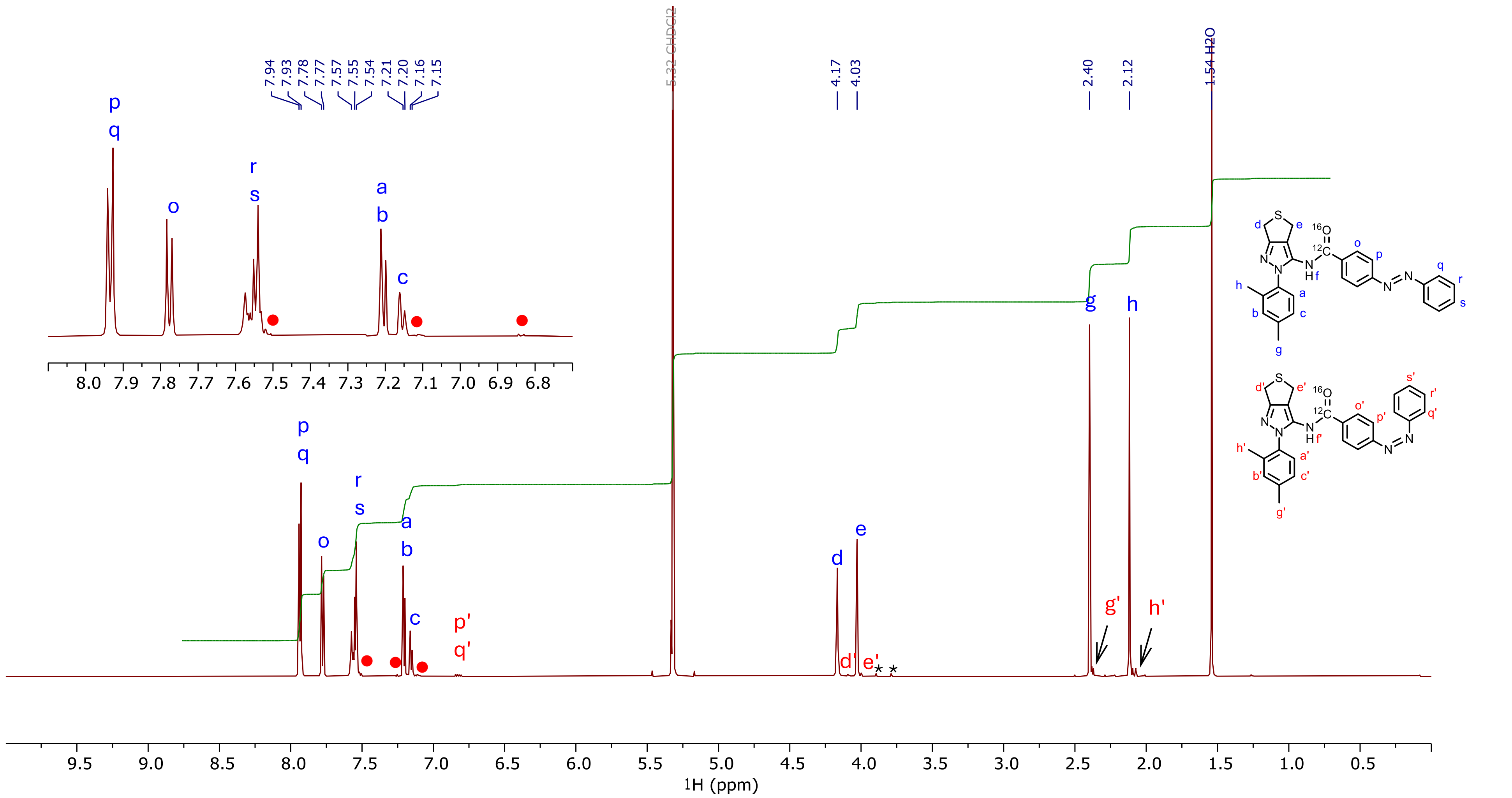

Observed Nucleus: 1H
Observed Frequency: 600.133705802 MHz

Solvent: CD2Cl2
Number of Scans: 64

Temperature: 297.999803841587 K
Pulse Sequence: zg30
Relaxation Delay (s): 1 sec

Figure S22a. ^{1}H NMR spectrum (600 MHz, CD_2Cl_2) of photopharmacological compound **1a**. The red dots (●) mark signals of the (*Z*)-isomer of photopharmacological compound **1a** and the asterisk (*) unidentified compounds.

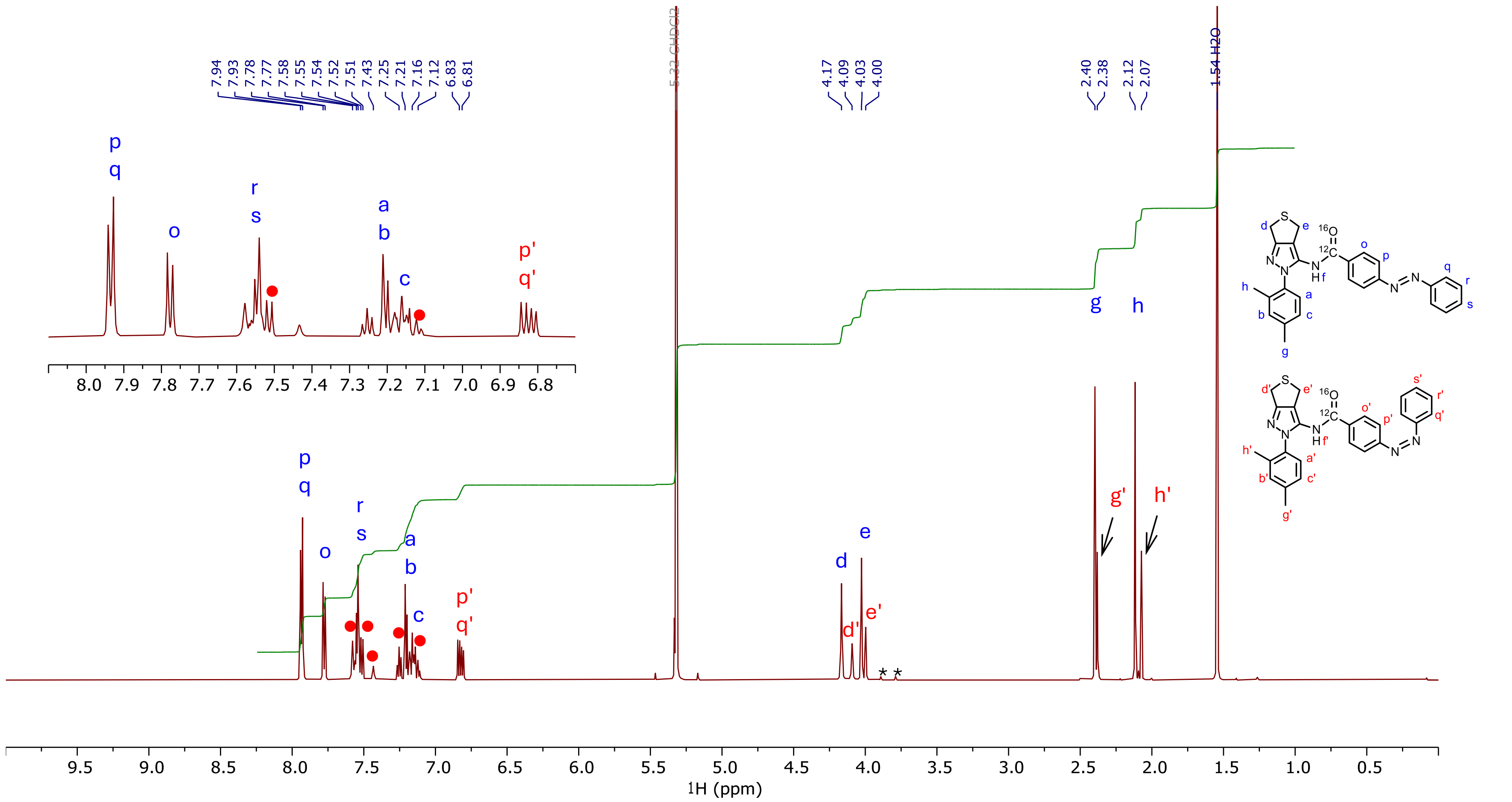

Observed Nucleus: 1H
Observed Frequency: 600.133705802 MHz

Solvent: CD2Cl2
Number of Scans: 64

Temperature: 298.000552865049 K
Pulse Sequence: zg30
Relaxation Delay (s): 1 sec

Figure S22b. ^{1}H NMR spectrum (600 MHz, CD_2Cl_2) of photopharmacological compound **1a** after illumination with light of a wavelength of 385 nm for 5 min. The red dots (●) mark signals of the (*Z*)-isomer of photopharmacological compound **1a** and the asterisk (*) unidentified compounds.

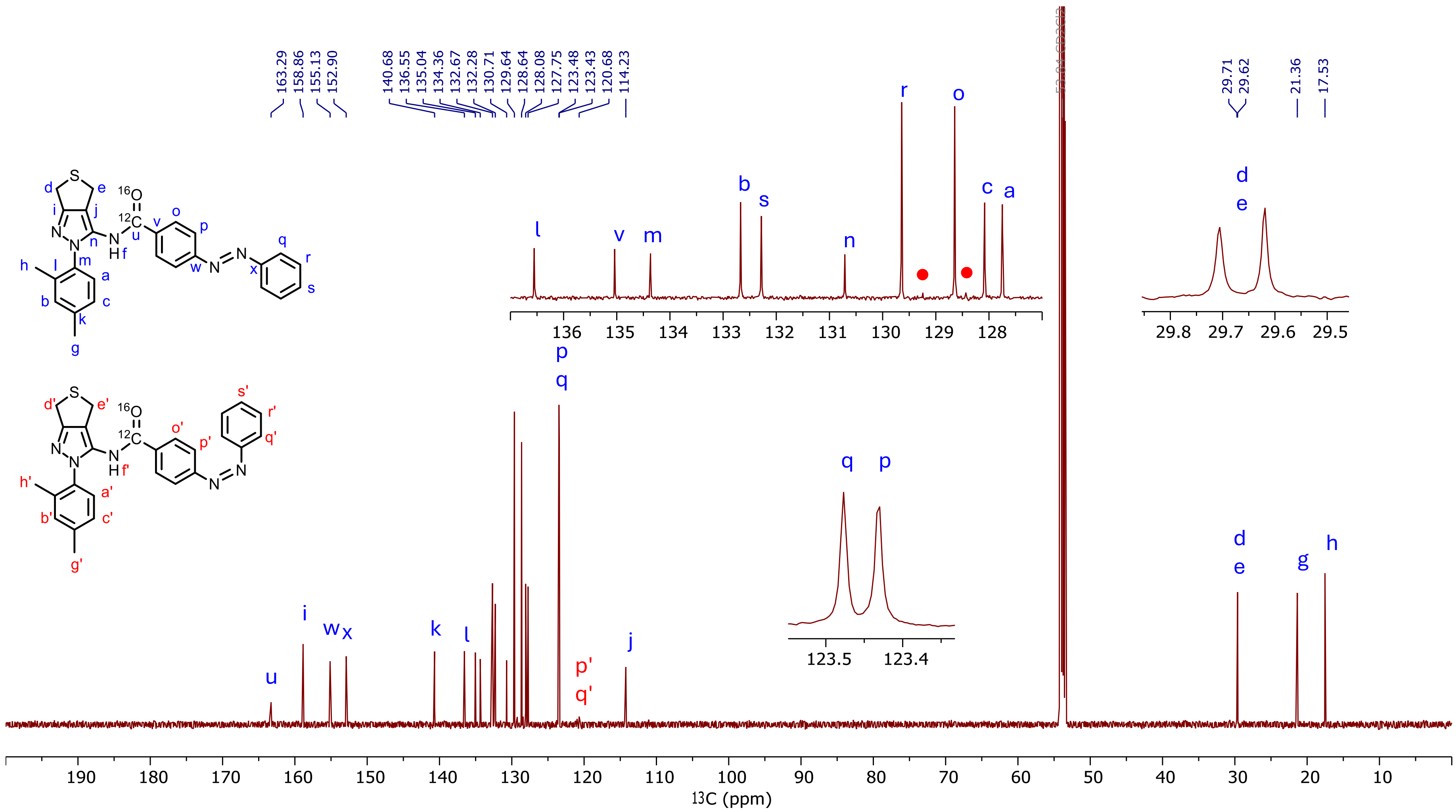

Observed Nucleus: 13C
Observed Frequency: 150.917898807 MHz

Solvent: CD2Cl2
Number of Scans: 2048

Temperature: 297.99870911499 K
Pulse Sequence: zgpg30
Relaxation Delay (s): 2 sec

Figure S22c. $^{13}C\{^1H\}$ NMR spectrum (151 MHz, CD_2Cl_2) of photopharmacological compound **1a**. The red dots (●) mark signals of the (*Z*)-isomer of photopharmacological compound **1a**.

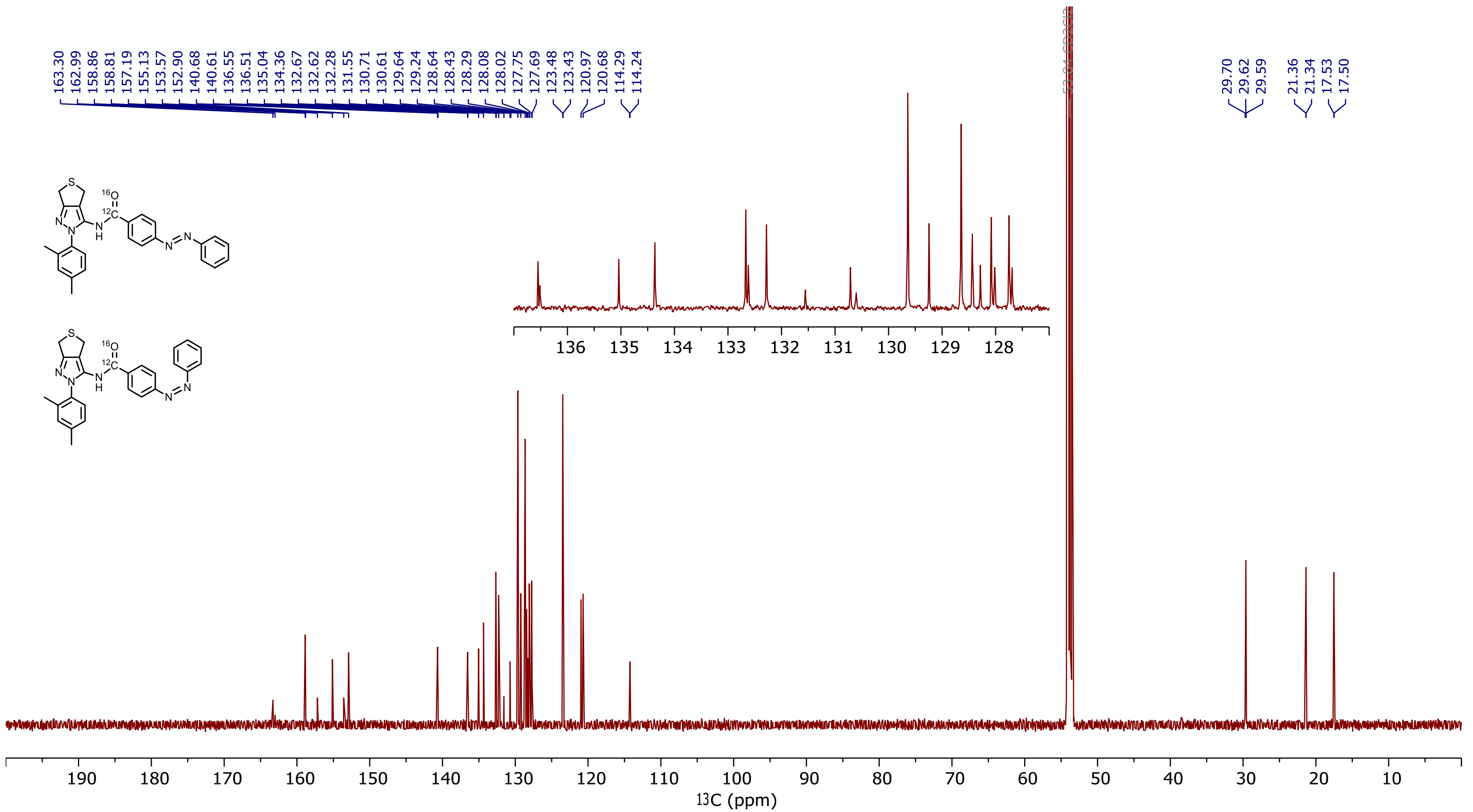

Observed Nucleus: 13C
Observed Frequency: 150.917898807 MHz

Solvent: CD2Cl2
Number of Scans: 2048

Temperature: 298.001532357268 K
Pulse Sequence: zgpg30
Relaxation Delay (s): 2 sec

Figure S22d. $^{13}C\{^{1}H\}$ NMR spectrum (151 MHz, CD_2Cl_2) of photopharmacological compound **1a** after illumination with light of a wavelength of 385 nm for 5 min.

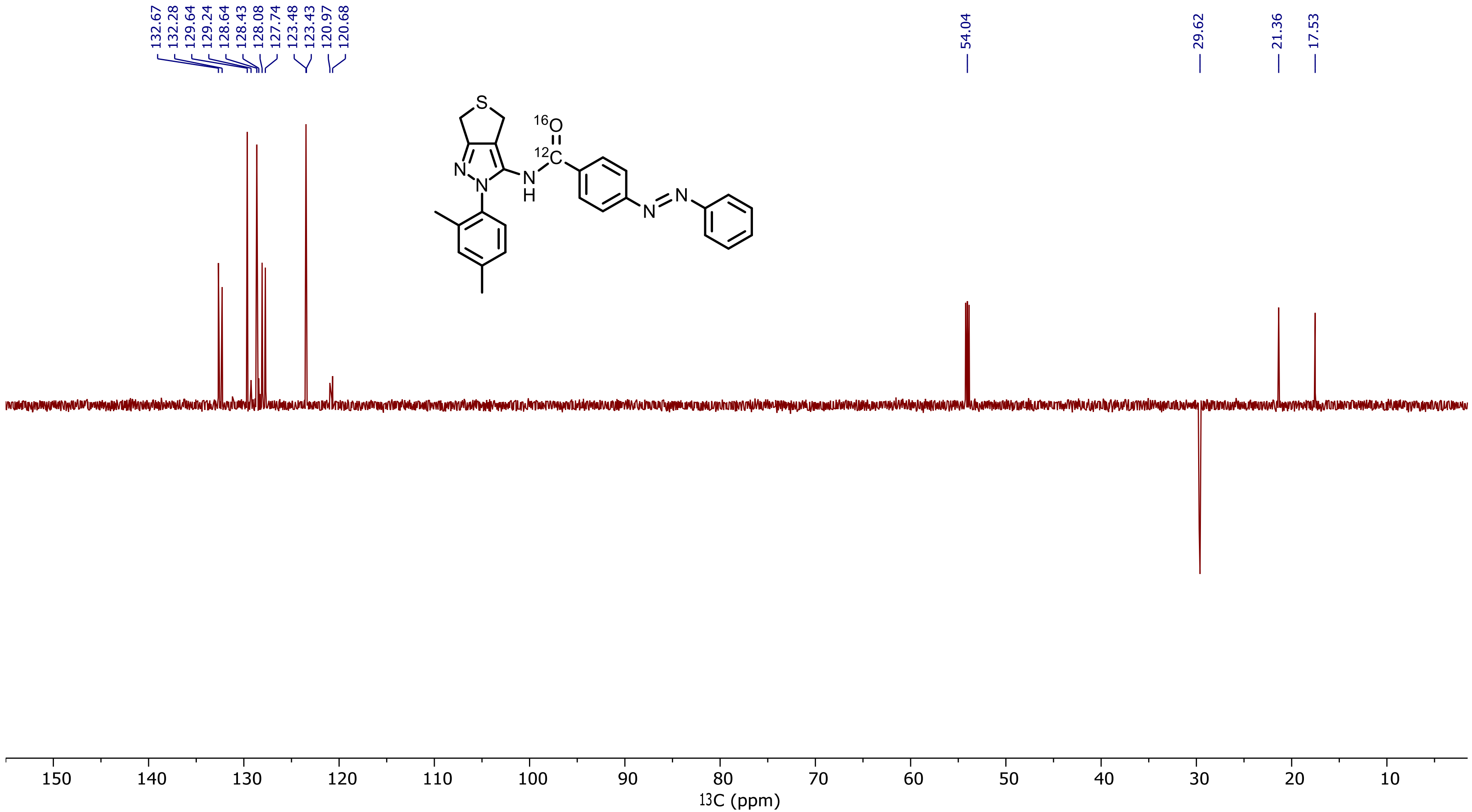

Observed Nucleus: 13C
Observed Frequency: 150.91488075 MHz

Solvent: CD2Cl2
Number of Scans: 256

Temperature: 298.000235970508 K
Pulse Sequence: deptsp135
Relaxation Delay (s): 2 sec

Figure S22e. ^{13}C DEPT 135 NMR spectrum (151 MHz, CD_2Cl_2) of photopharmacological compound **1a**.

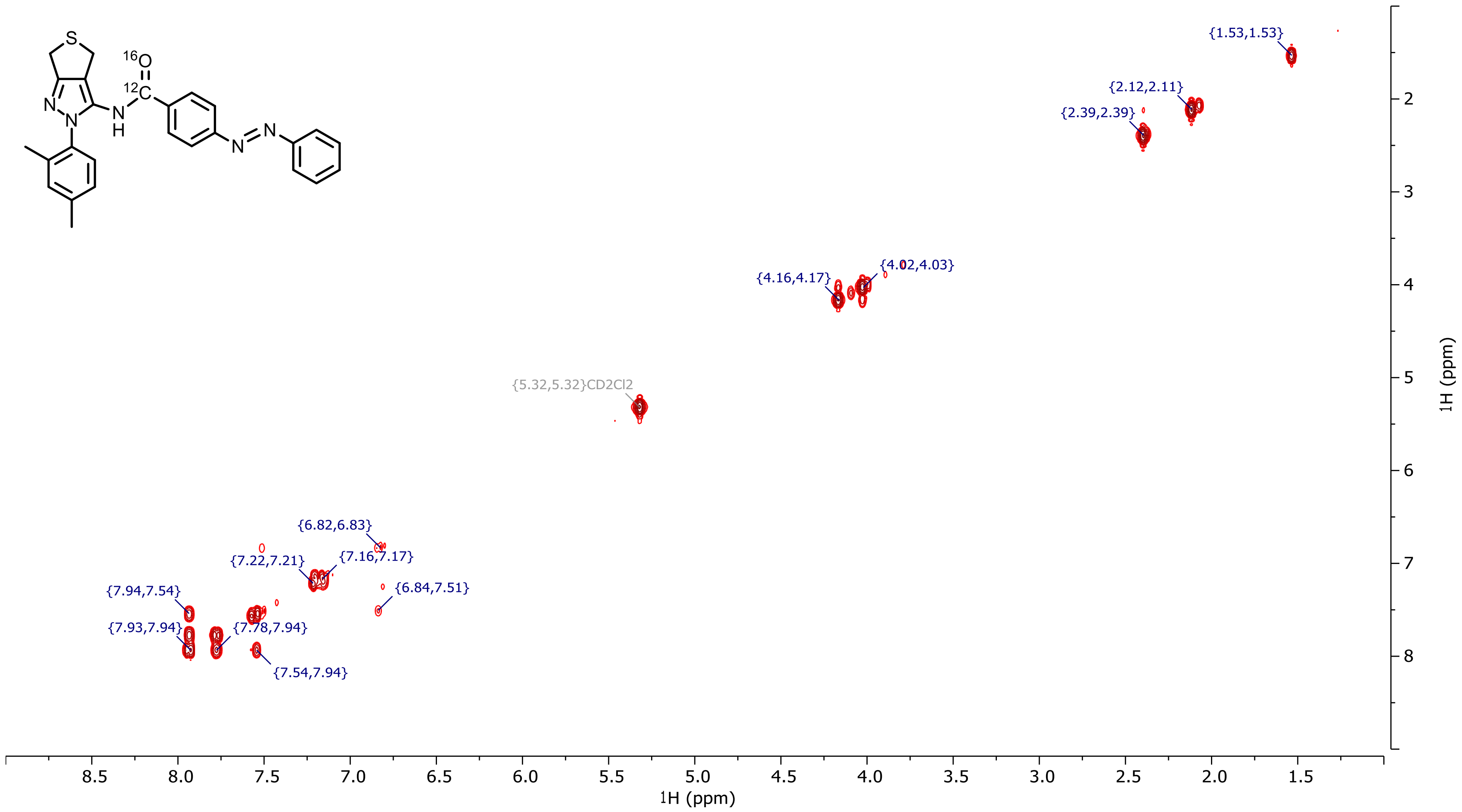

Observed Nucleus: [1H, 1H]
Observed Frequency: [600.13330072, 600.13330072] MHz

Solvent: CD2Cl2
Number of Scans: 2

Temperature: 297.999400521262 K
Pulse Sequence: cosygpppqf
Relaxation Delay (s): 2 sec

Figure S22f. ^{1}H ^{1}H COSY NMR spectrum (600 MHz, 600 MHz, CD_2Cl_2) of photopharmacological compound **1a**.

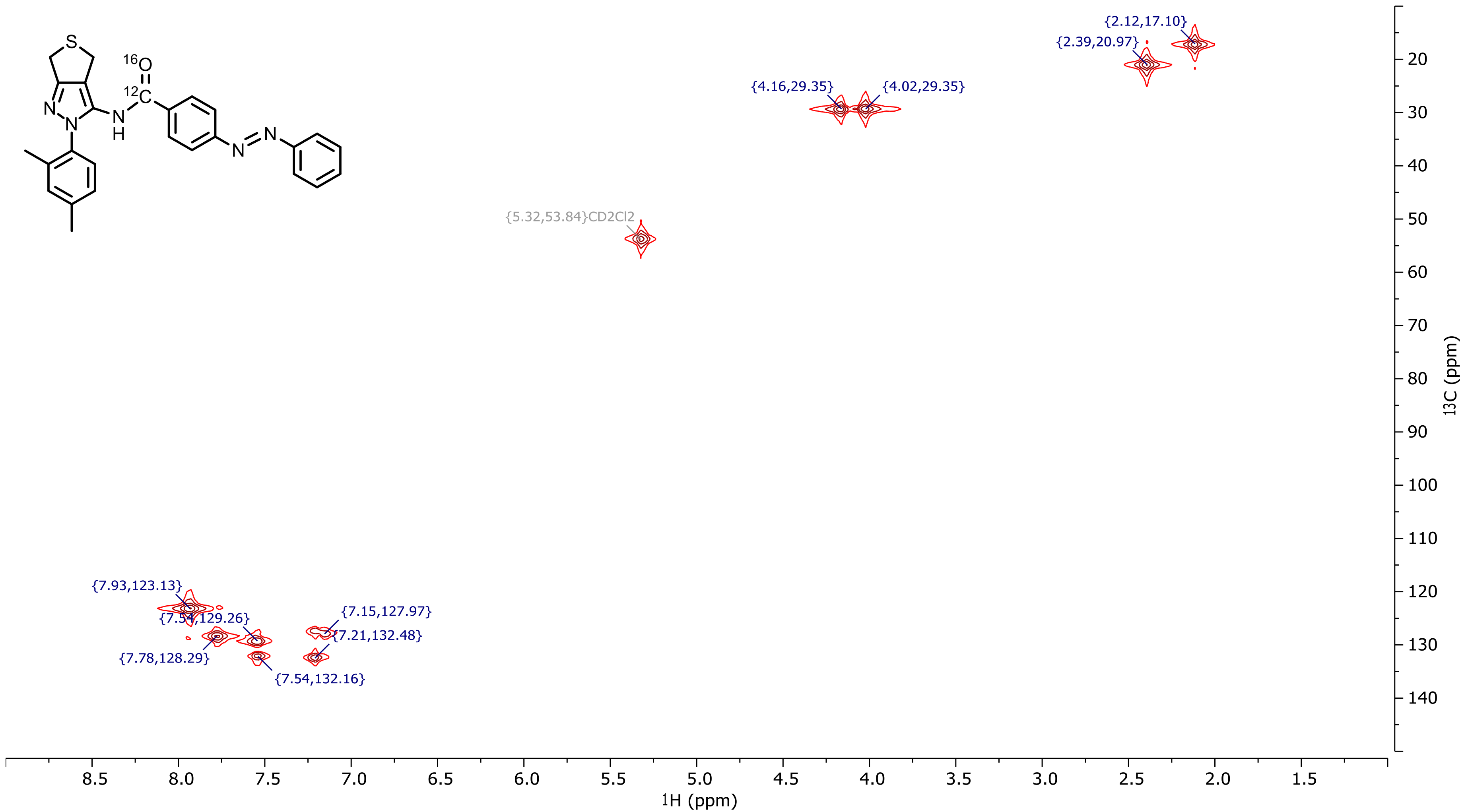

Observed Nucleus: [13C, 1H]
Observed Frequency: [150.91412671, 600.13330072] MHz
Solvent: CD2Cl2
Number of Scans: 4
Temperature: 297.999227669694 K
Pulse Sequence: hmqcgpqf
Relaxation Delay (s): 1.5 sec

Figure S22g. ^{13}C ^{1}H HMQC NMR spectrum (151 MHz, 600 MHz, CD_2Cl_2) of photopharmacological compound **1a**.

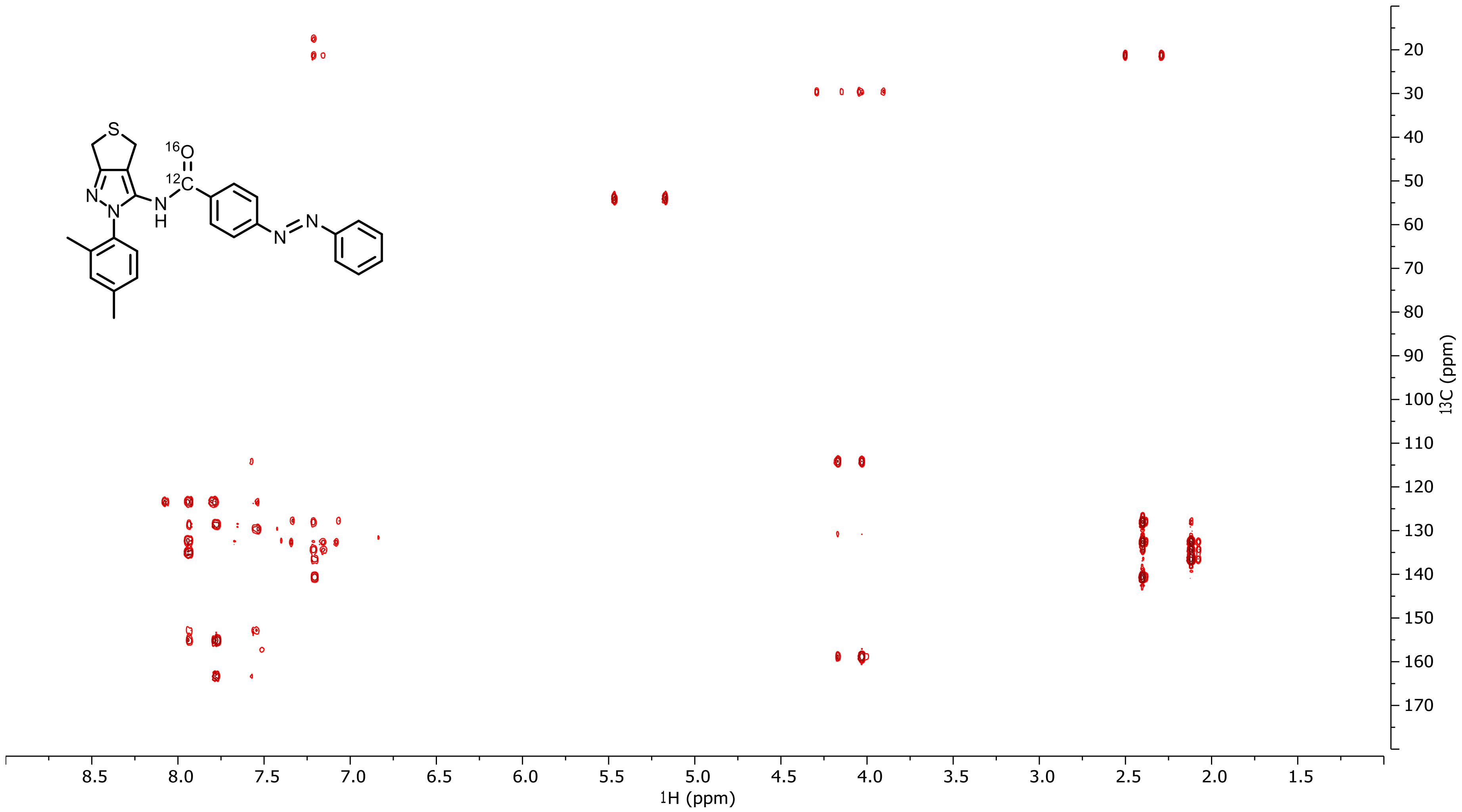

Observed Nucleus: [13C, 1H]
Observed Frequency: [150.92016282, 600.13330072] MHz

Solvent: CD2Cl2
Number of Scans: 8

Temperature: 298.000235970508 K
Pulse Sequence: hmbcgpndqf
Relaxation Delay (s): 1.5 sec

Figure S22h. ^{13}C ^{1}H HMBC NMR spectrum (151 MHz, 600 MHz, CD_2Cl_2) of photopharmacological compound **1a**.

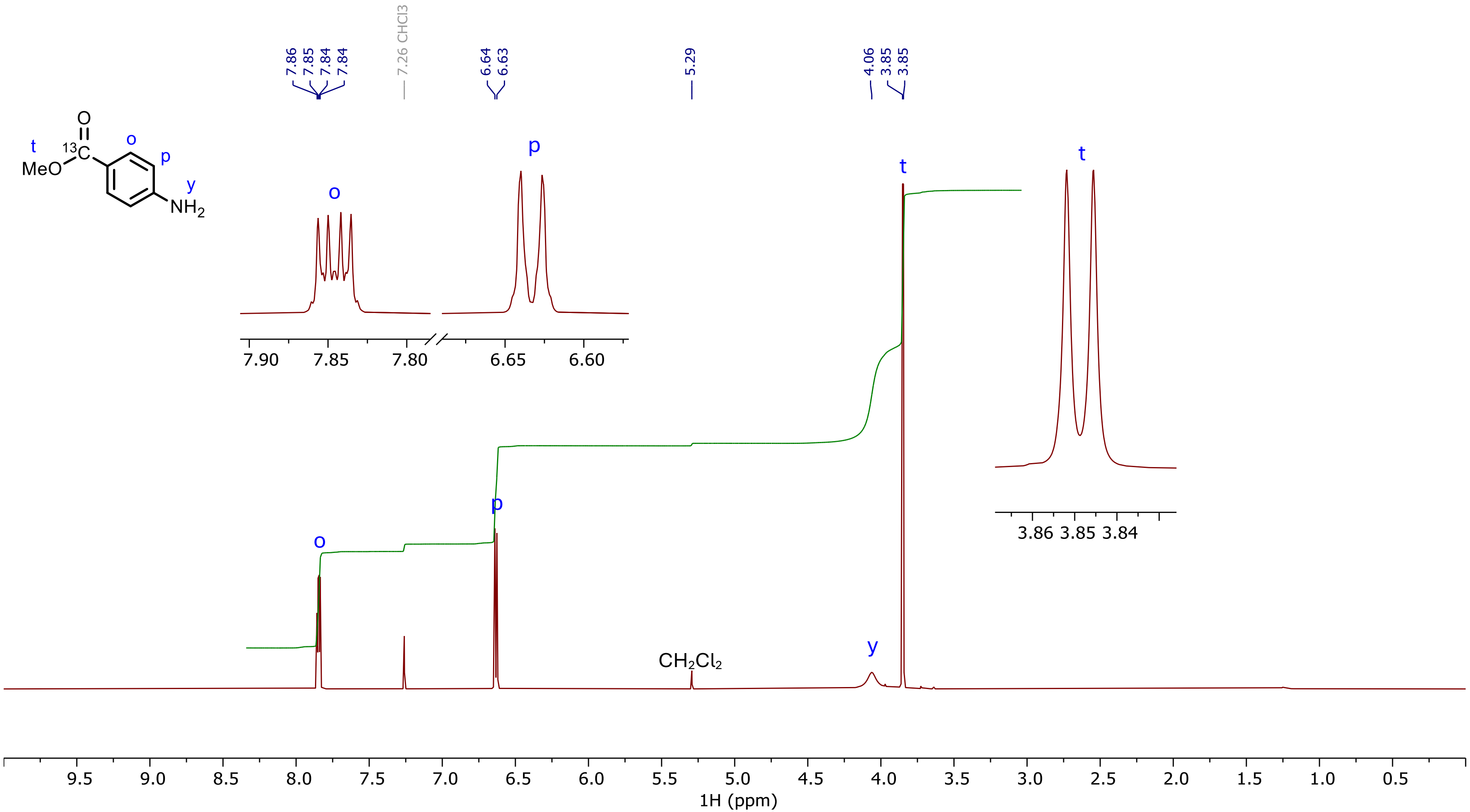

Observed Nucleus: 1H
Observed Frequency: 600.133705802 MHz

Solvent: CDCl3
Number of Scans: 64

Temperature: 298.000322396291 K
Pulse Sequence: zg30
Relaxation Delay (s): 1 sec

Figure S23a. ^{1}H NMR spectrum (600 MHz, $CDCl_3$) of methyl 4-aminobenzene-1-[^{13}C]carboxylate (**2b**).

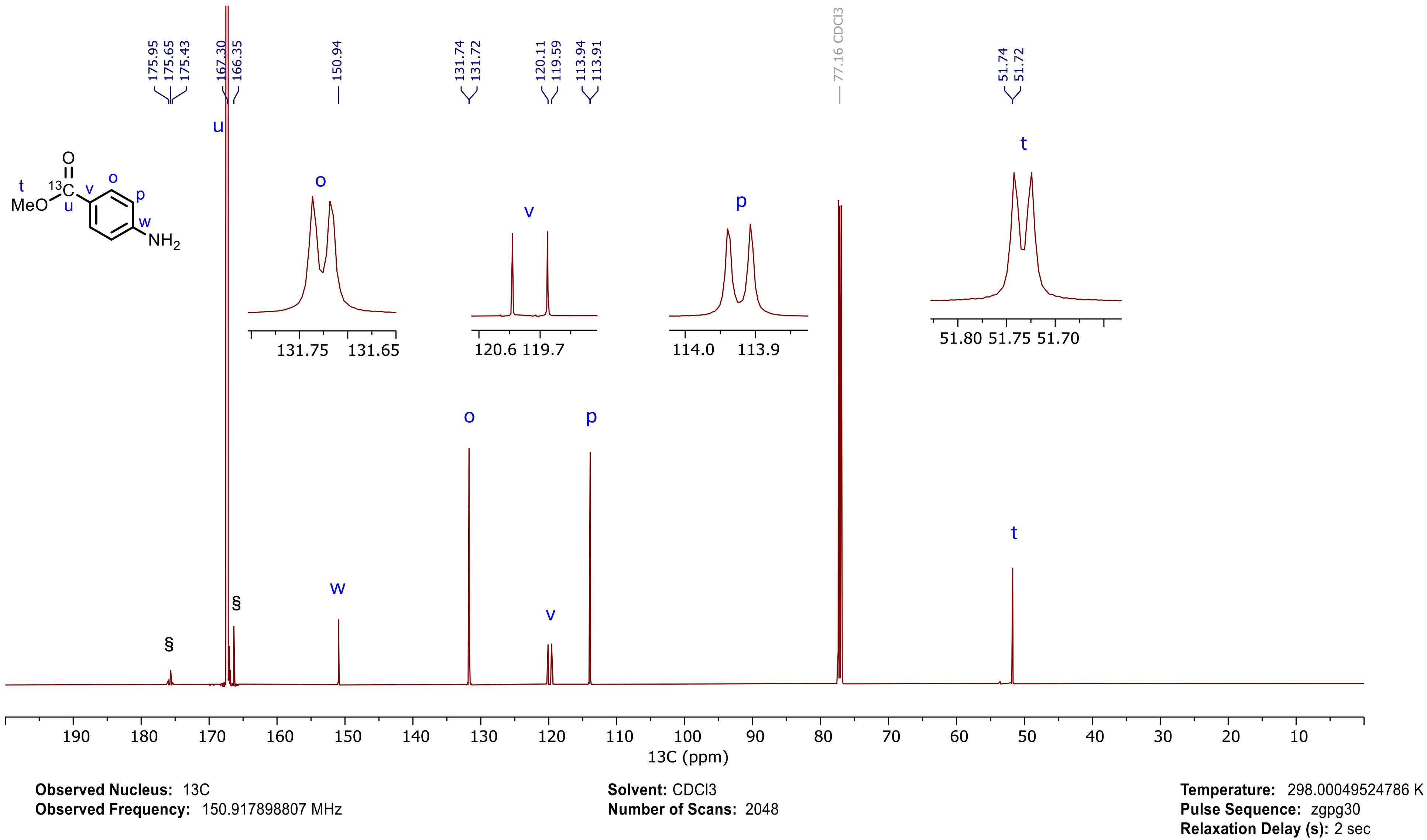

Figure S23b. ^{13}C NMR spectrum (151 MHz, $CDCl_3$) of methyl 4-aminobenzene-1-[^{13}C]carboxylate (**2b**). The section sign (§) mark signals of unidentified compounds. We assume that these signals are caused by contaminants already present in the commercially obtained material and that these signals are only seen because of ^{13}C enrichment. Otherwise we would expect to detect additional signals. Therefore the amount of the contaminants must be extremely small.

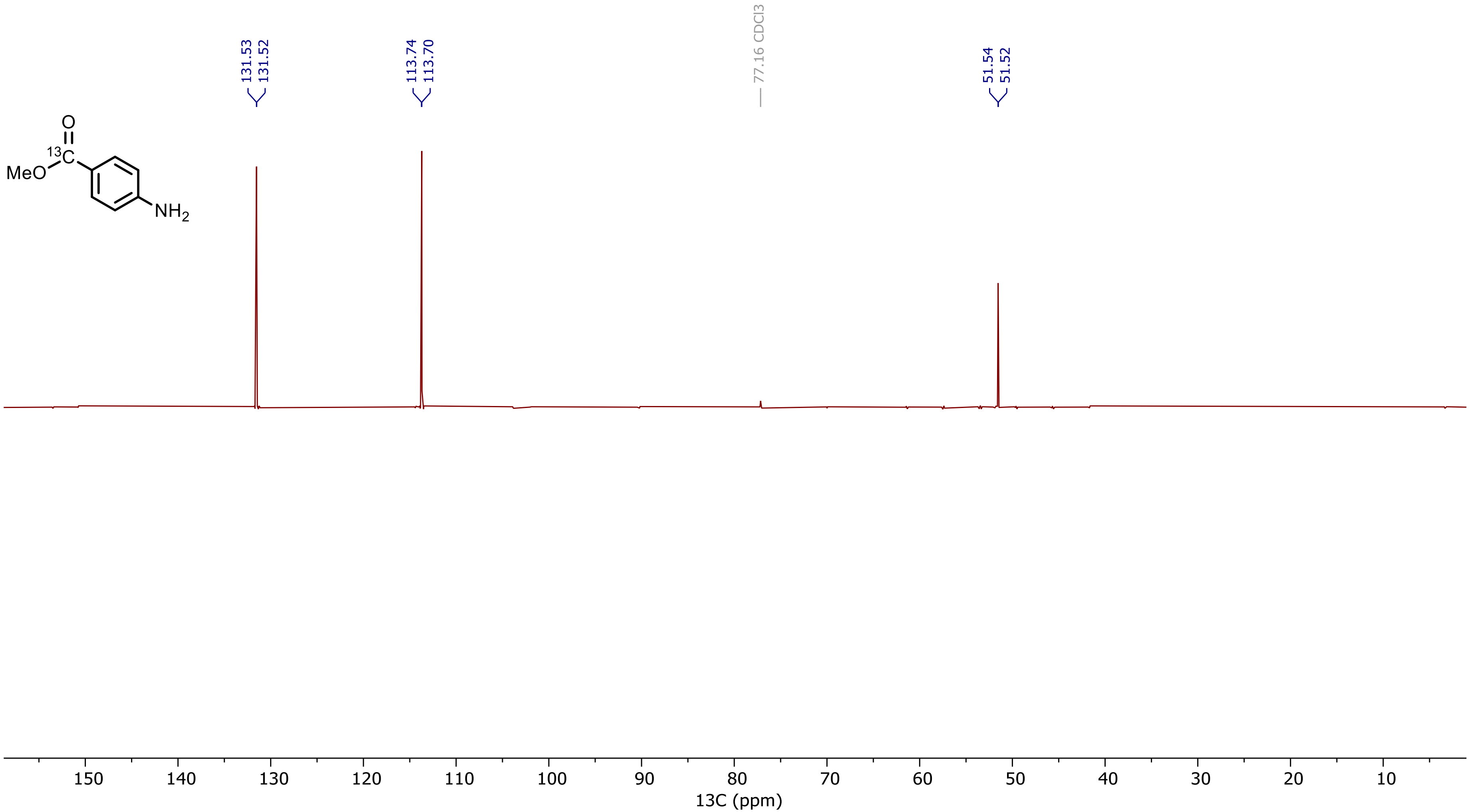

Observed Nucleus: 13C
Observed Frequency: 150.91488075 MHz

Solvent: CDCl3
Number of Scans: 512

Temperature: 298.000149544724 K
Pulse Sequence: deptsp135
Relaxation Delay (s): 2 sec

Figure S23c. ^{13}C DEPT 135 NMR spectrum (151 MHz, $CDCl_3$) of methyl 4-aminobenzene-1-[^{13}C]carboxylate (**2b**).

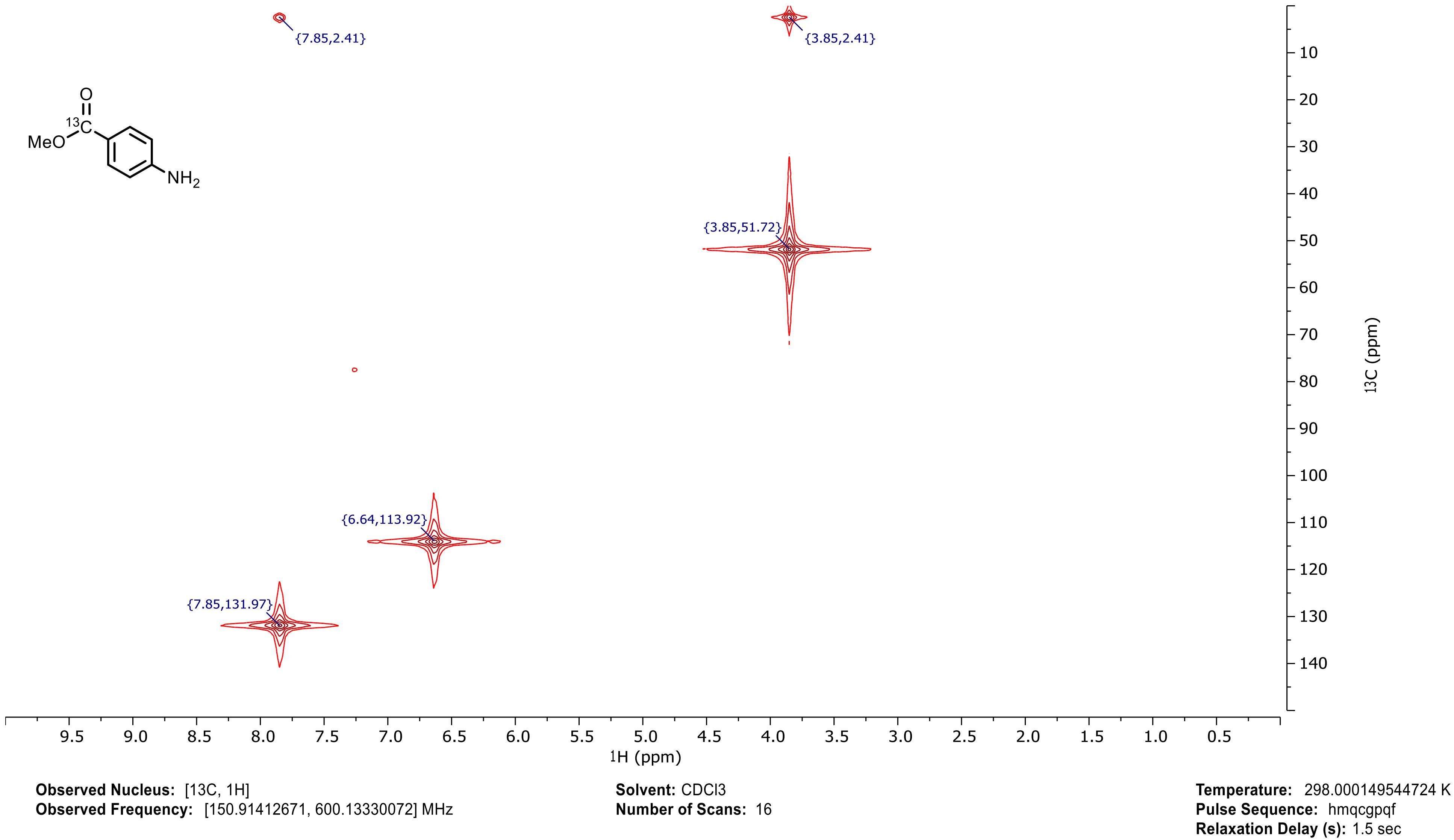

Figure S23d. ^{13}C ^{1}H HMQC NMR spectrum (151 MHz, 600 MHz, $CDCl_3$) of methyl 4-aminobenzene-1-[^{13}C]carboxylate (**2b**).

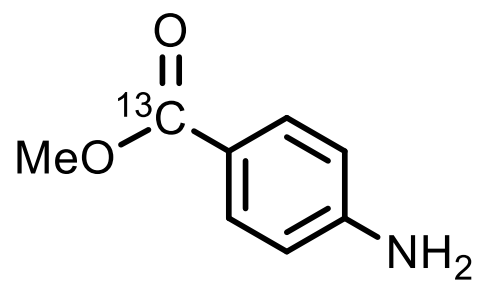

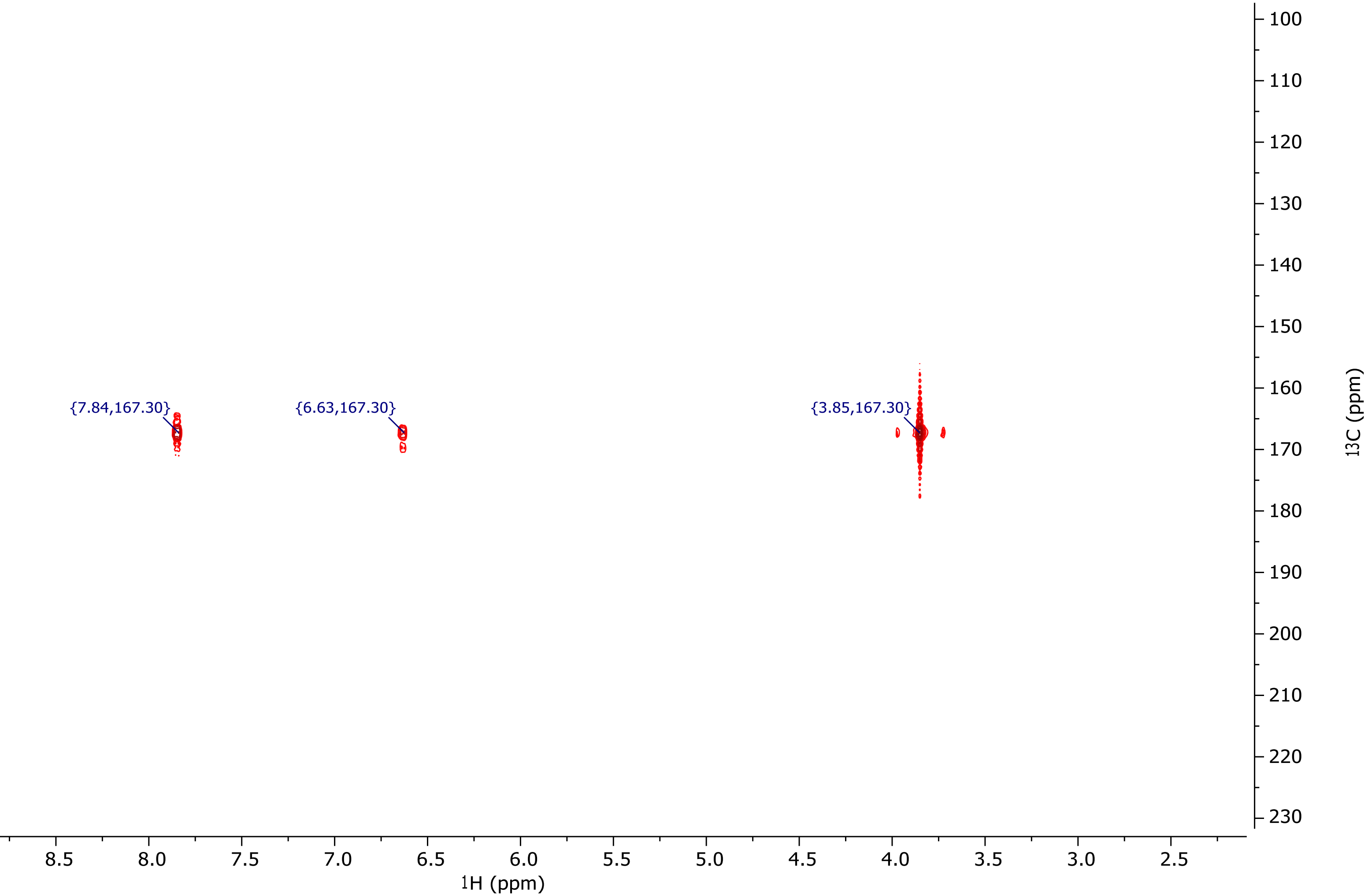

Observed Nucleus: [13C, 1H]
Observed Frequency: [150.92016282, 600.13330072] MHz

Solvent: CDCl3
Number of Scans: 32

Temperature: 297.999400521262 K
Pulse Sequence: hmbcgpndqf
Relaxation Delay (s): 1.5 sec

Figure S23e. ^{13}C ^{1}H HMBC NMR spectrum (151 MHz, 600 MHz, $CDCl_3$) of methyl 4-aminobenzene-1-[^{13}C]carboxylate (**2b**).

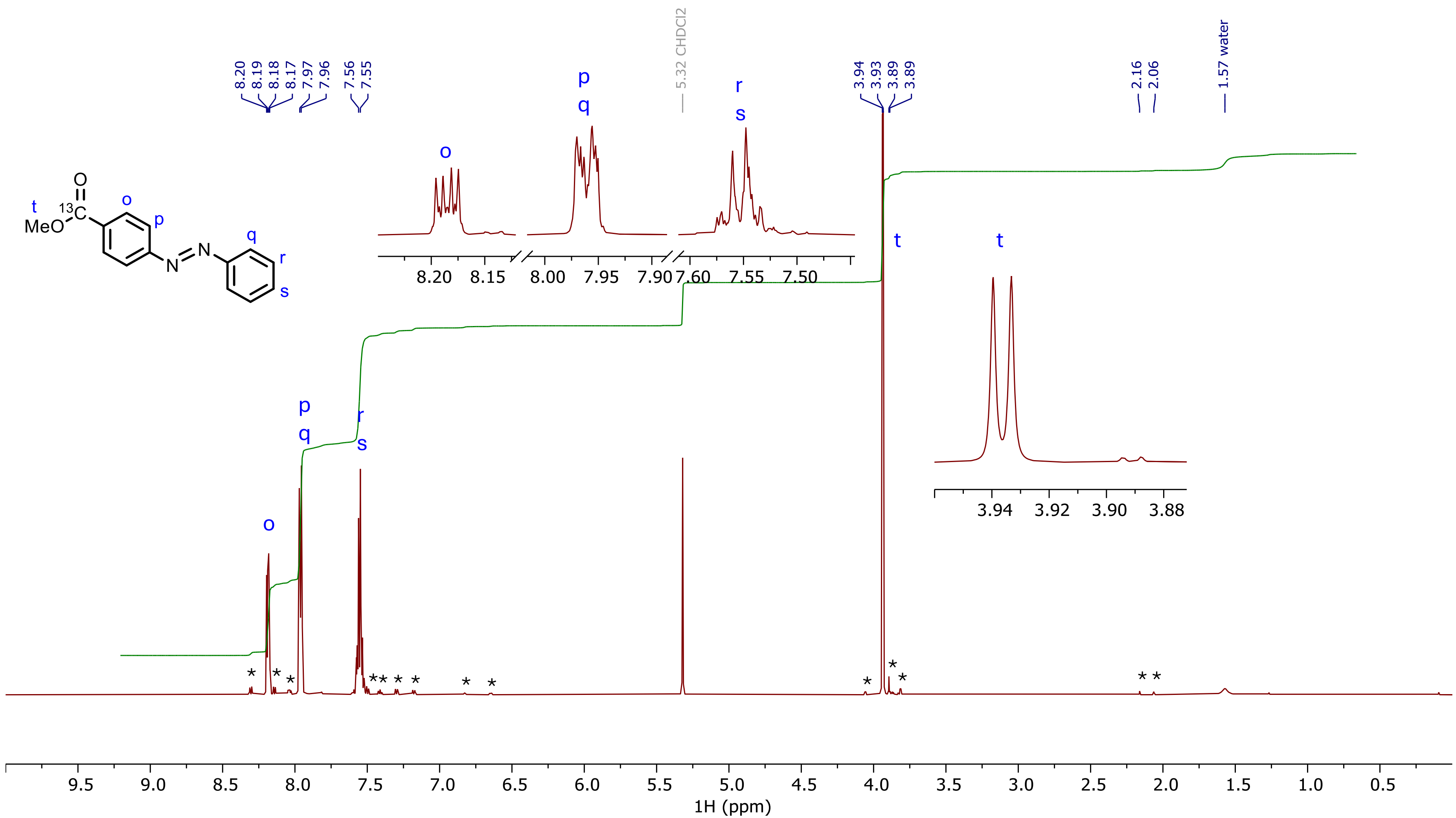

Observed Nucleus: 1H
Observed Frequency: 600.133705802 MHz

Solvent: CD2Cl2
Number of Scans: 32

Temperature: 298.00049524786 K
Pulse Sequence: zg30
Relaxation Delay (s): 1 sec

Figure S24a. ^{1}H NMR spectrum (600 MHz, CD_2Cl_2) of methyl (*E*)-azobenzene-4-[^{13}C]carboxylate (**3b**). The asterisk (*) mark signals of unidentified compounds.

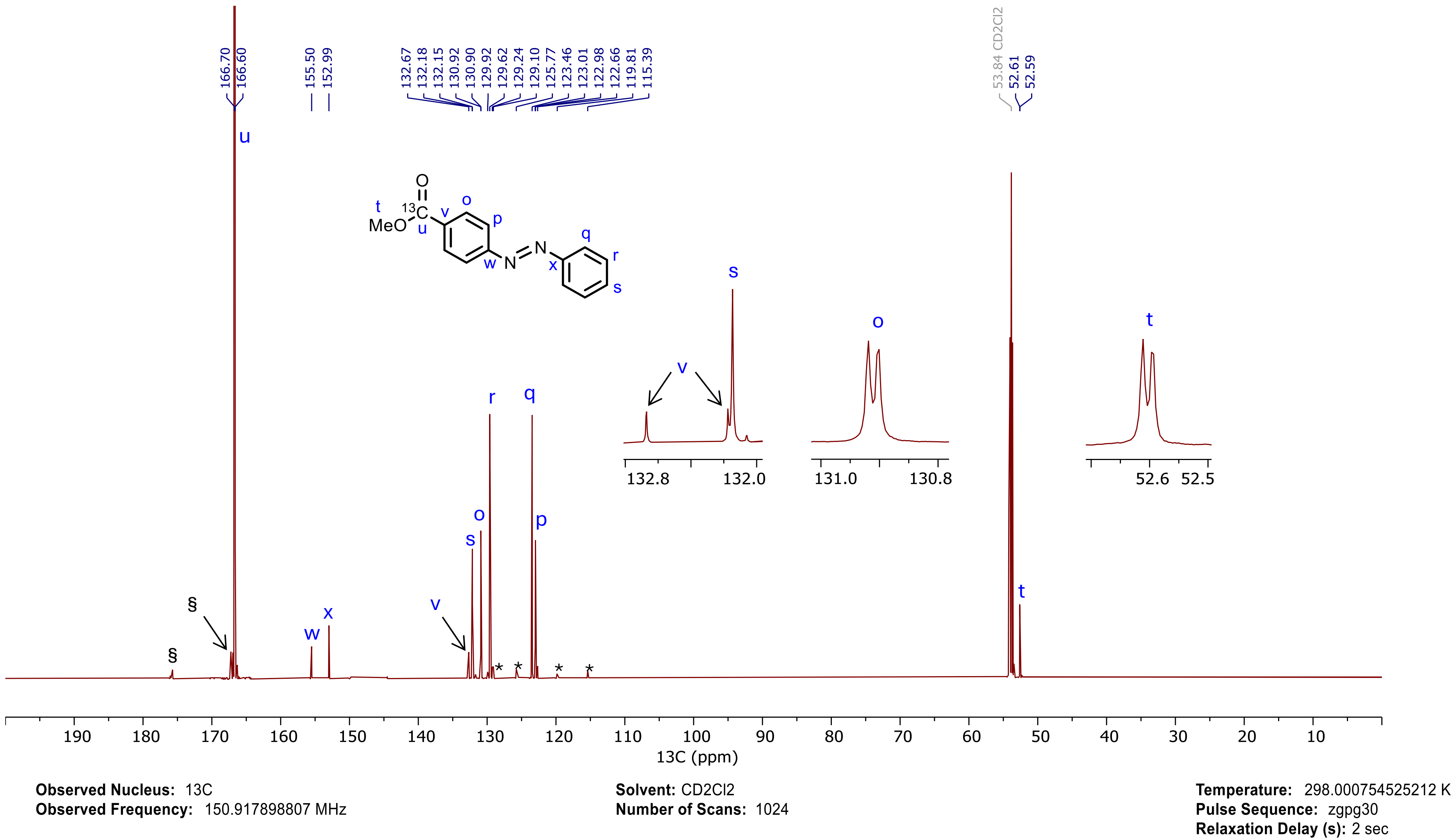

Figure S24b. $^{13}C\{^1H\}$ NMR spectrum (151 MHz, CD_2Cl_2) of methyl (*E*)-azobenzene-4-[^{13}C]carboxylate (**3b**). The asterisk (*) mark signals of unidentified compounds. §: We assume that these signals are caused by contaminants already present in the commercially obtained material and that these signals are only seen because of ^{13}C enrichment (see Figure S13b).

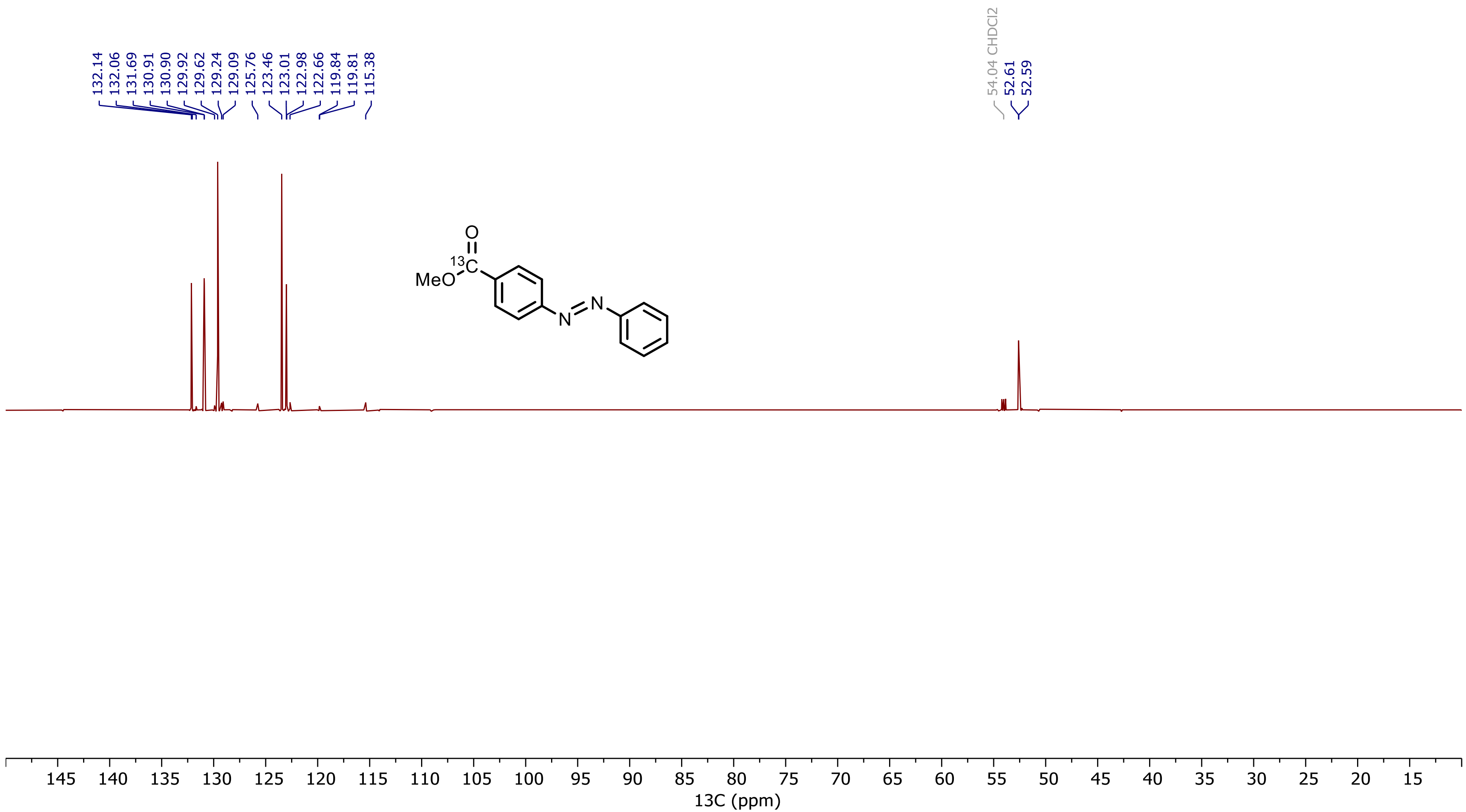

Observed Nucleus: 13C
Observed Frequency: 150.91488075 MHz

Solvent: CD2Cl2
Number of Scans: 256

Temperature: 297.998622689206 K
Pulse Sequence: deptsp135
Relaxation Delay (s): 2 sec

Figure S24c. ^{13}C DEPT 135 NMR spectrum (151 MHz, CD_2Cl_2) of methyl (*E*)-azobenzene-4-[^{13}C]carboxylate (**3b**).

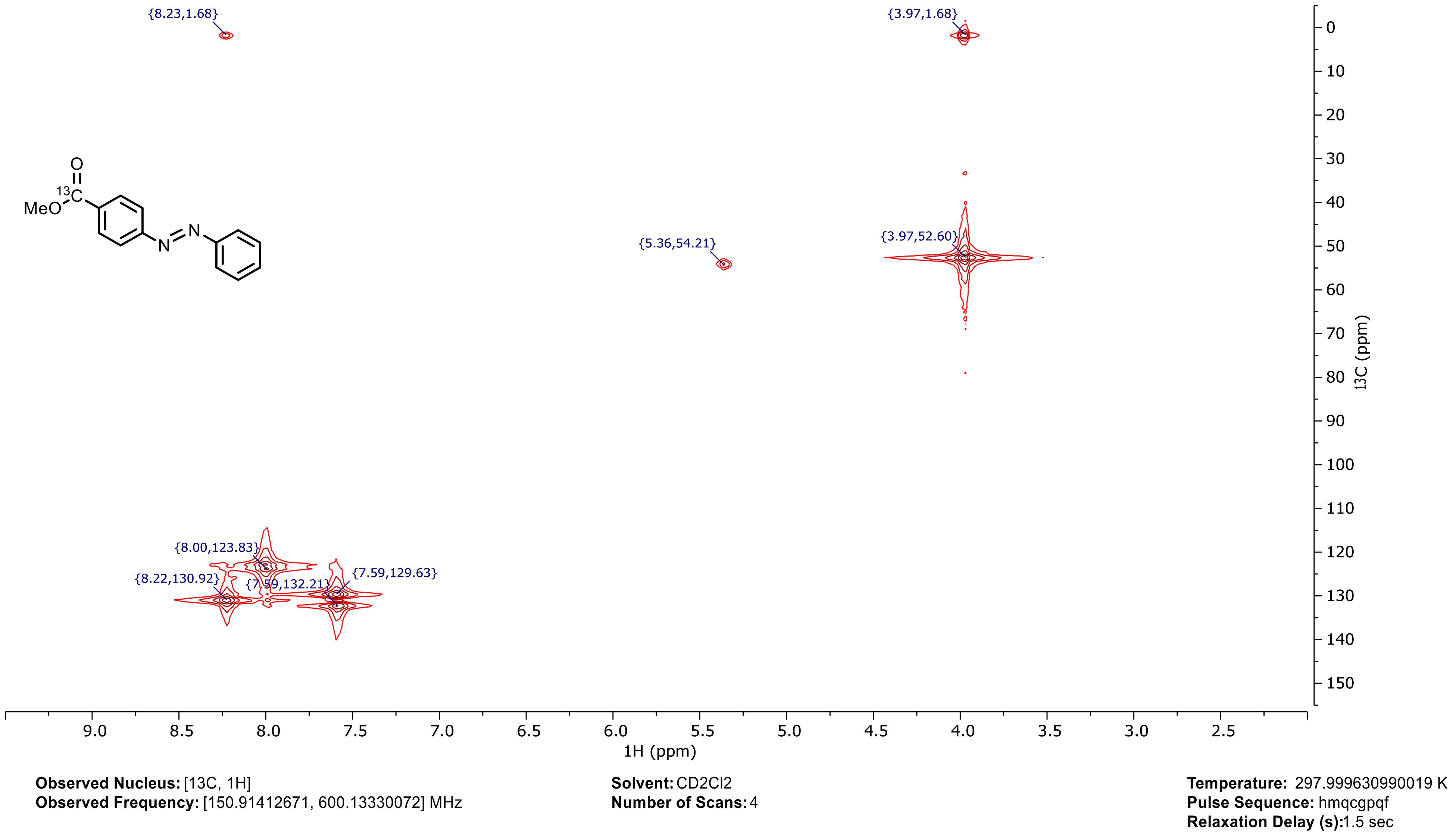

Figure S24d. ^{13}C ^{1}H HMQC 135 NMR spectrum (151 MHz, 600 MHz, CD_2Cl_2) of methyl (*E*)-azobenzene-4-[^{13}C]carboxylate (**3b**).

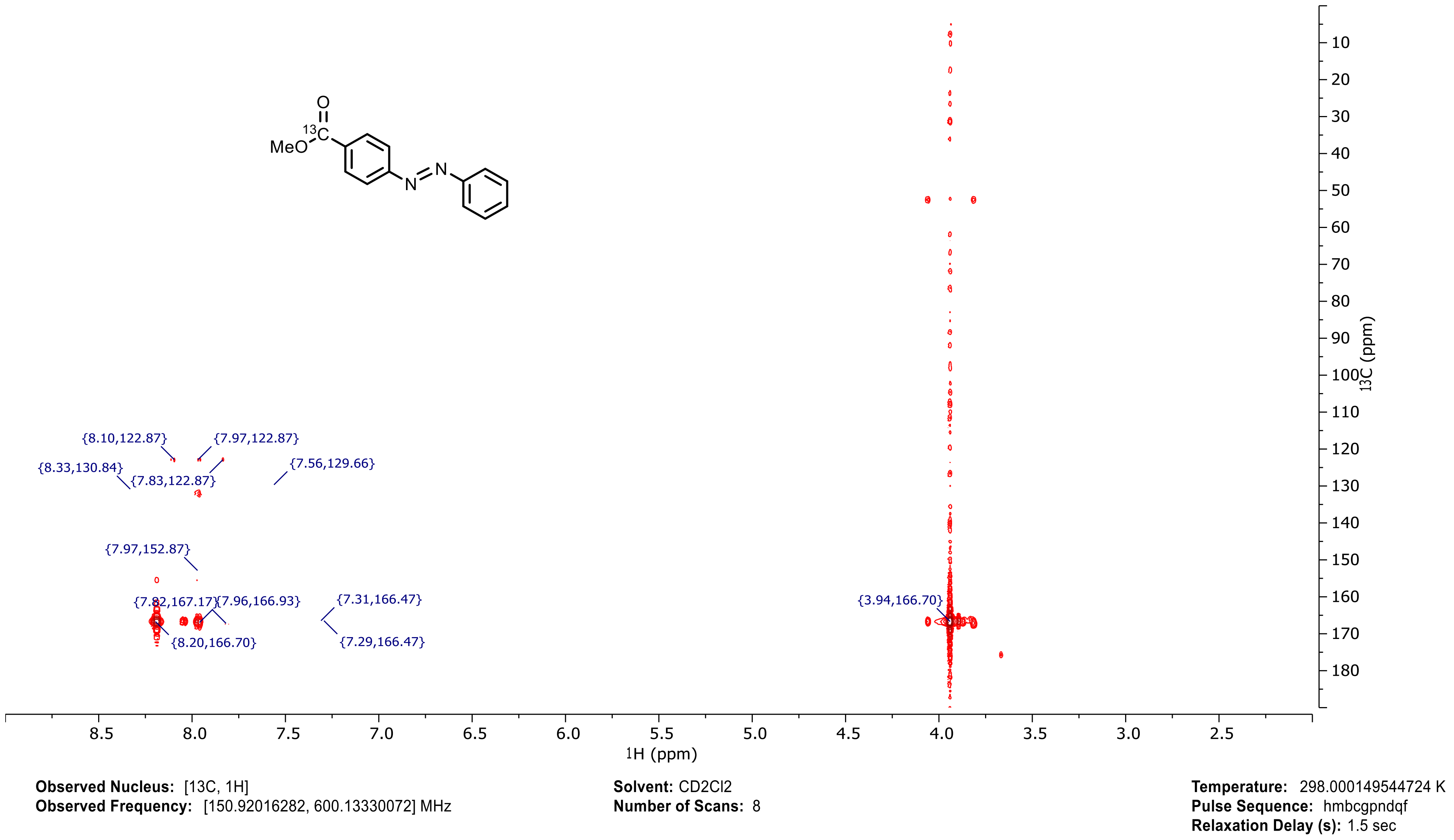

Figure S24e. ^{13}C ^{1}H HMBC 135 NMR spectrum (151 MHz, 600 MHz, CD_2Cl_2) of methyl (*E*)-azobenzene-4-[^{13}C]carboxylate (**3b**).

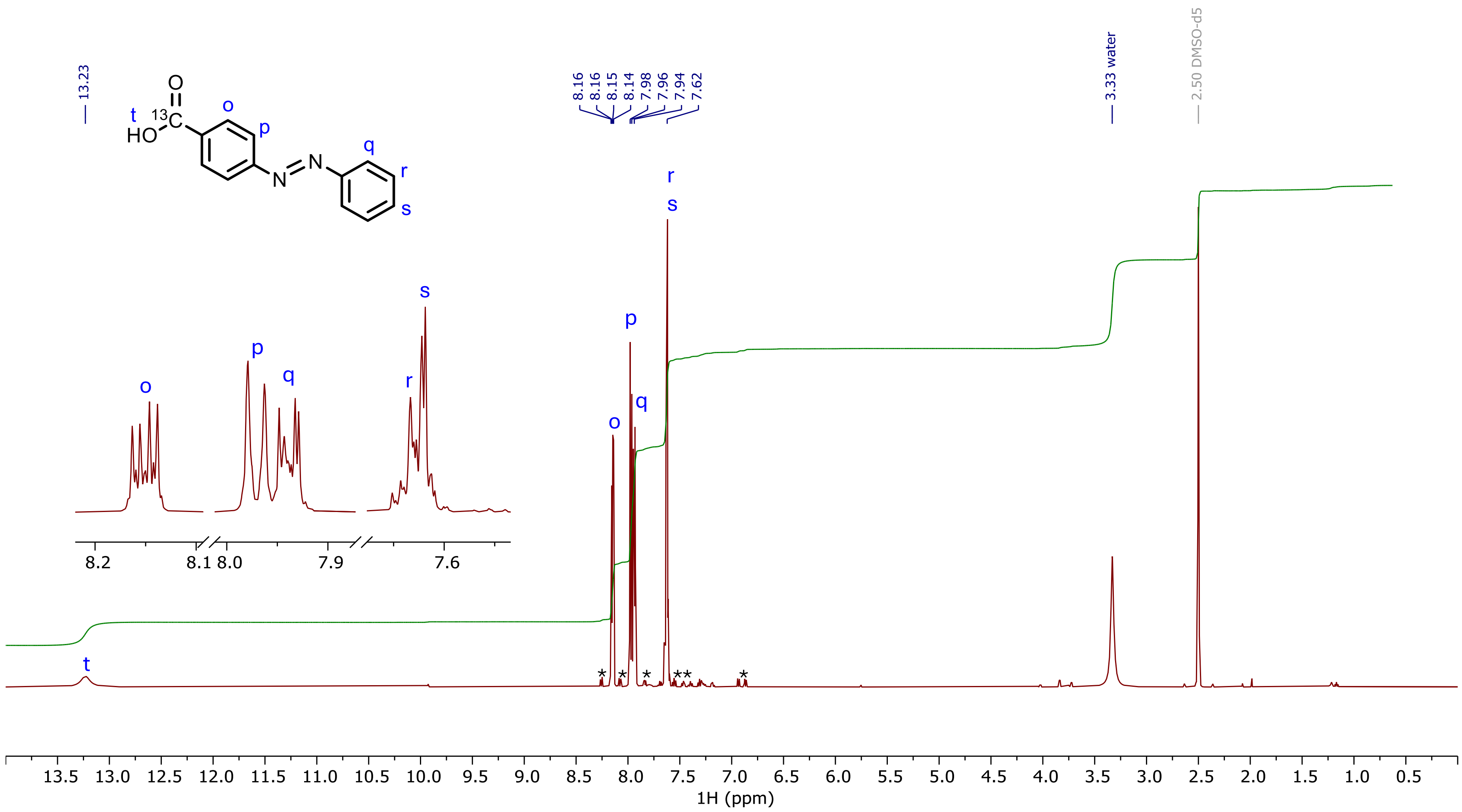

Observed Nucleus: 1H
Observed Frequency: 500.163088488 MHz
Solvent: DMSO
Number of Scans: 64
Temperature: 298.000070719421 K
Pulse Sequence: zg30
Relaxation Delay (s): 1 sec

Figure S25a. ^{1}H NMR spectrum (500 MHz, DMSO) of (*E*)-azobenzene-4-[^{13}C]carboxylic acid (**4c**). The asterisk (*) mark signals of unidentified compounds.

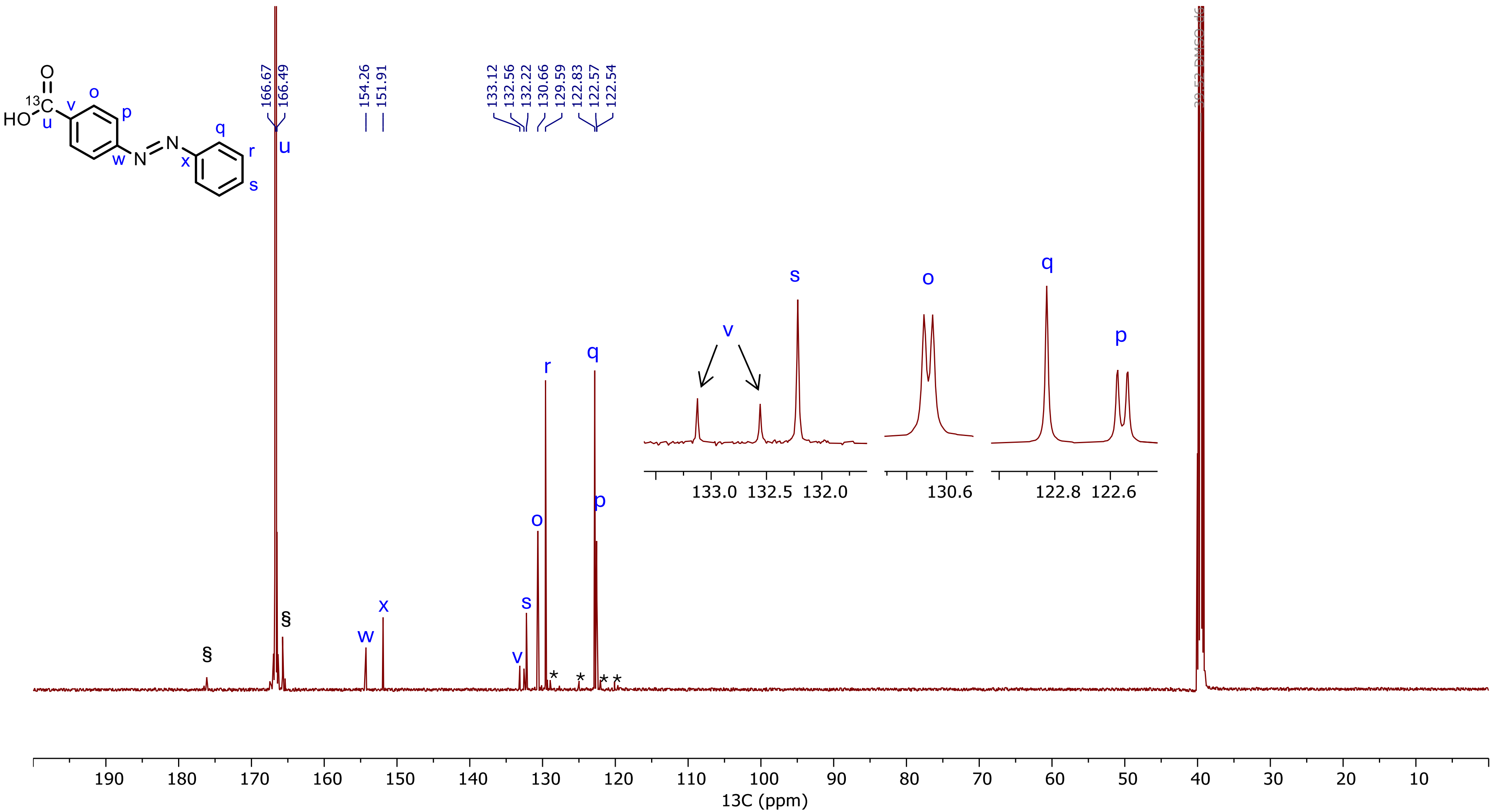

Observed Nucleus: 13C
Observed Frequency: 125.777908565 MHz

Solvent: DMSO
Number of Scans: 4096

Temperature: 297.997358715441 K
Pulse Sequence: zgpg30
Relaxation Delay (s): 2 sec

Figure S25b. $^{13}C\{^{1}H\}$ NMR spectrum (126 MHz, DMSO) of (*E*)-azobenzene-4-[^{13}C]carboxylic acid (**4c**). The asterisk (*) mark signals of unidentified compounds. §: We assume that these signals are caused by contaminants already present in the commercially obtained material and that these signals are only seen because of ^{13}C enrichment (see Figure S13b).

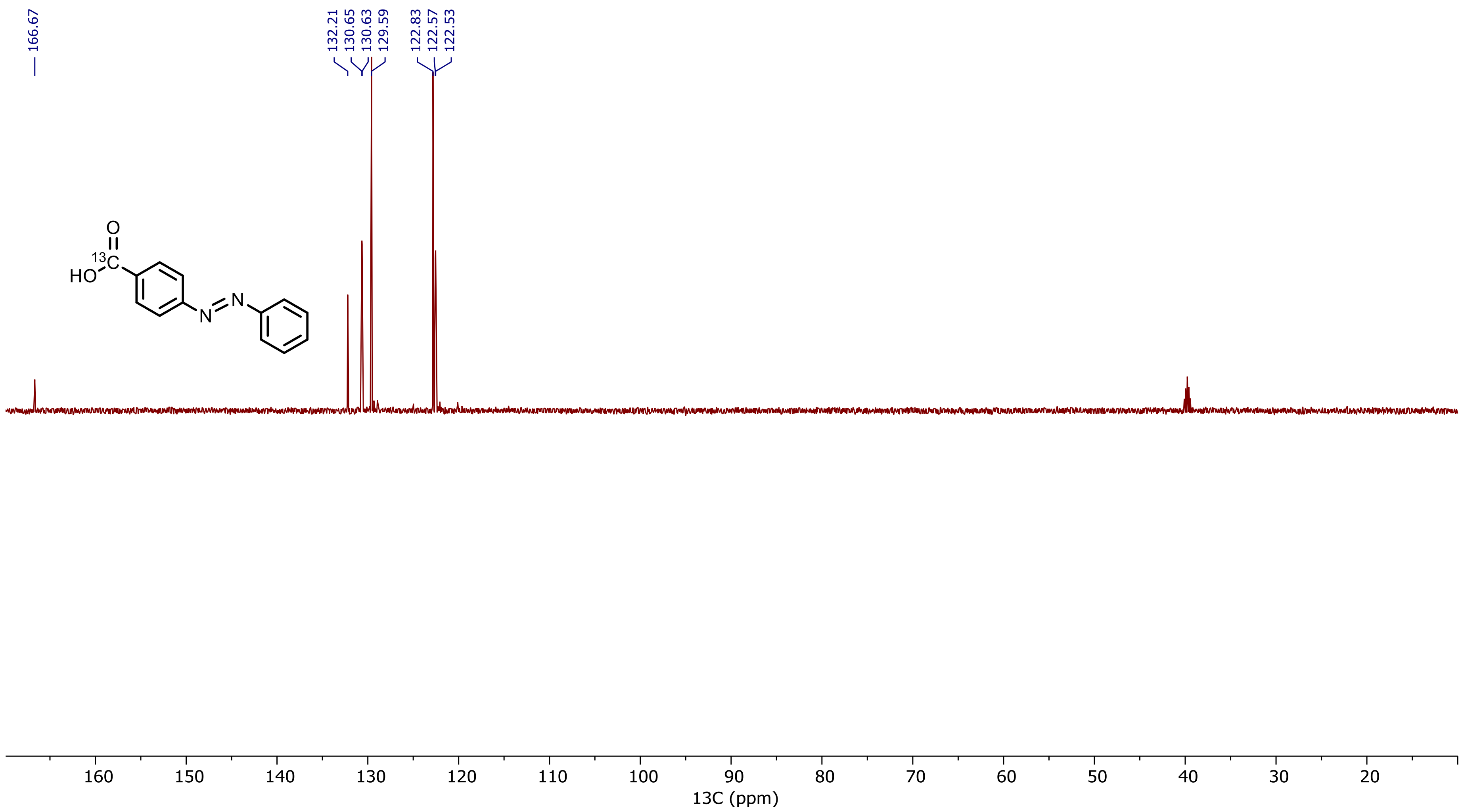

Observed Nucleus: 13C
Observed Frequency: 125.775393258 MHz

Solvent: DMSO
Number of Scans: 512

Temperature: 298.016855010723 K
Pulse Sequence: deptsp135
Relaxation Delay (s): 2 sec

Figure S25c. ^{13}C DEPT 135 NMR spectrum (126 MHz, DMSO) of (*E*)-azobenzene-4-[^{13}C]carboxylic acid (**4c**).

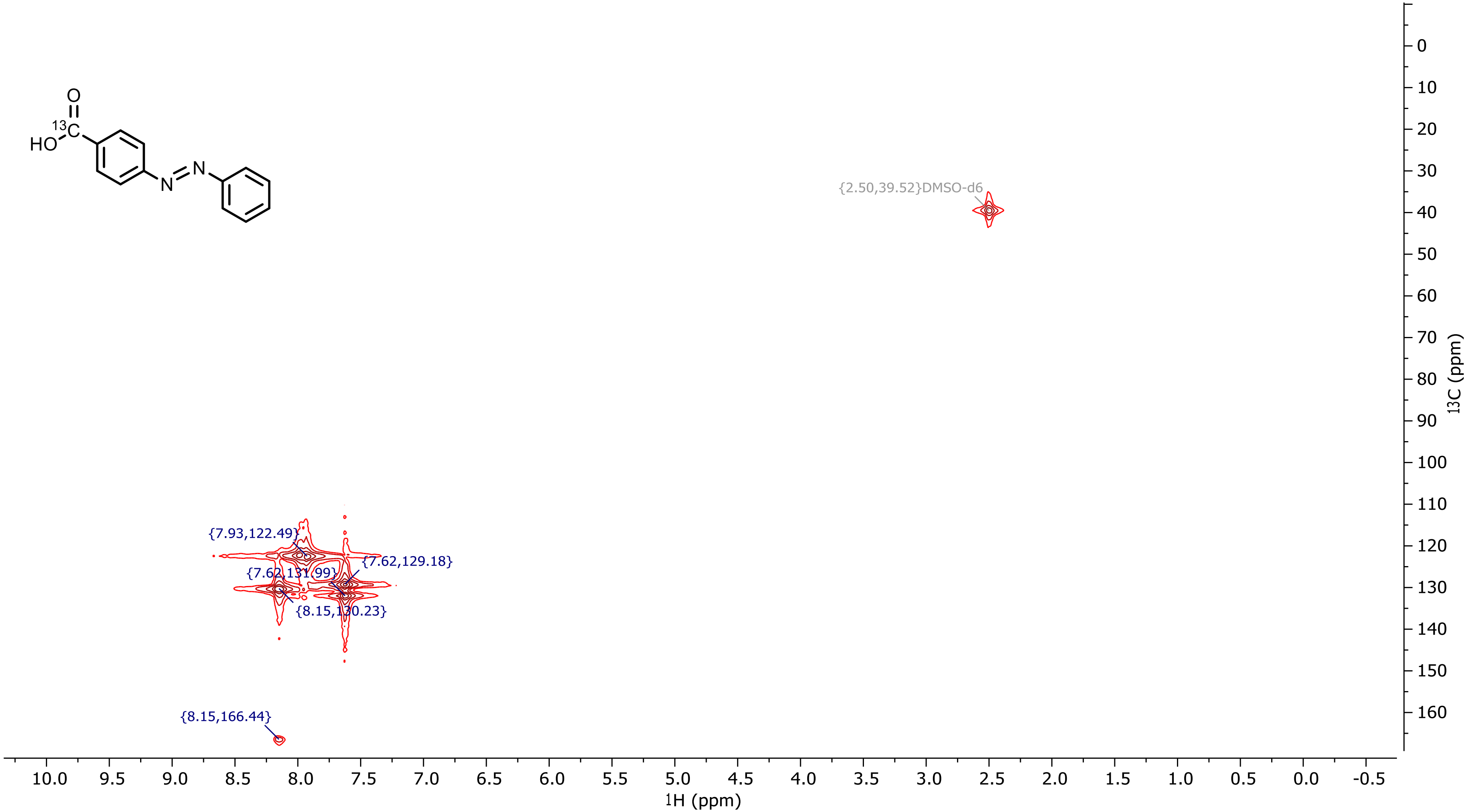

Observed Nucleus: [13C, 1H]
Observed Frequency: [125.775393258563, 500.162400768] MHz

Solvent: DMSO
Number of Scans: 8

Temperature: 298.032433966922 K
Pulse Sequence: hmqcgpqf
Relaxation Delay (s): 1.5 sec

Figure S25d. ^{13}C ^{1}H HMQC NMR spectrum (126 MHz, 500 MHz, DMSO) of (*E*)-azobenzene-4-[^{13}C]carboxylic acid (**4c**).

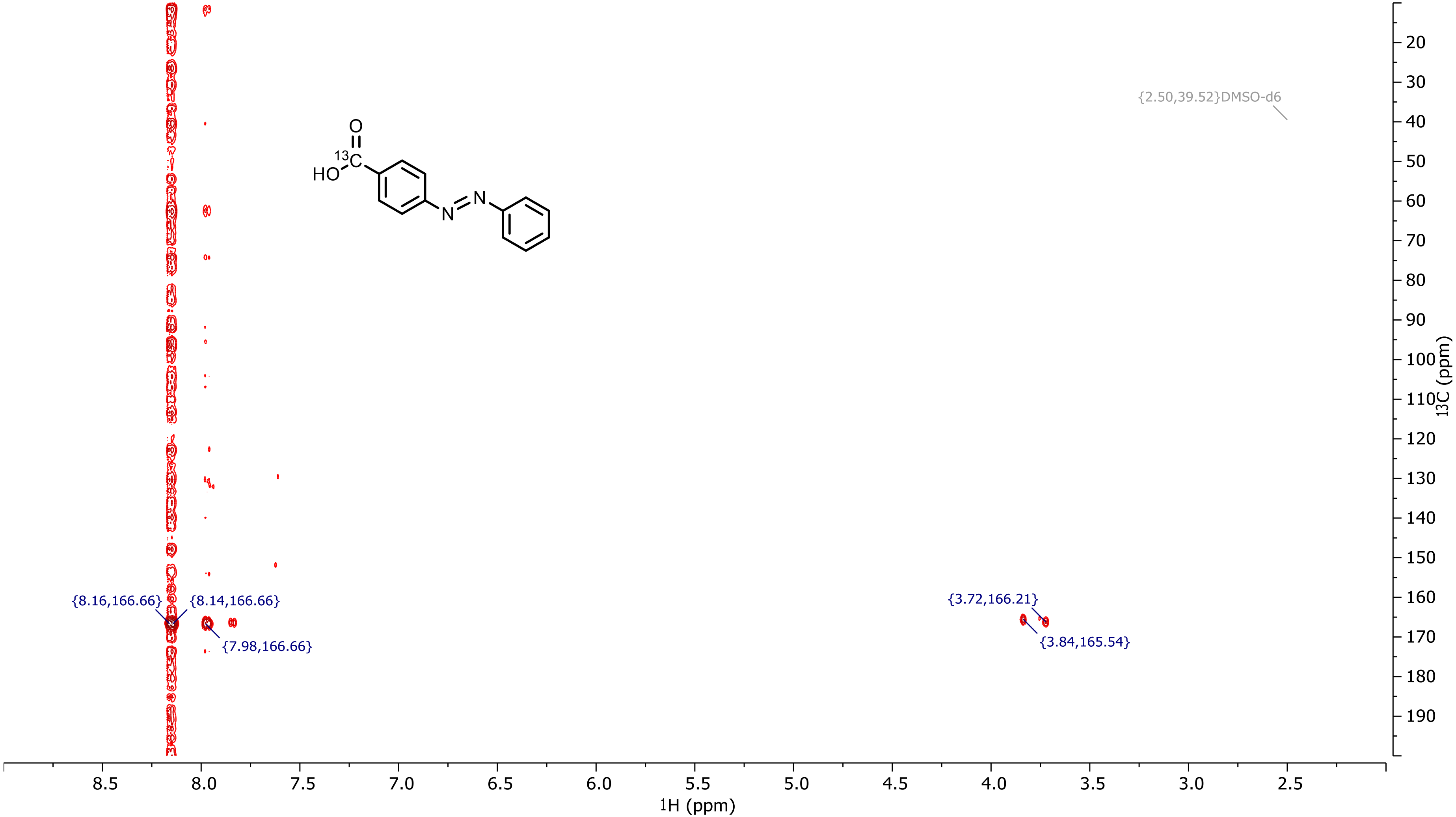

Observed Nucleus: [13C, 1H]
Observed Frequency: [125.778537391863, 500.162400768] MHz

Solvent: DMSO
Number of Scans: 16

Temperature: 298.00181845532 K
Pulse Sequence: hmbcgplpndqf
Relaxation Delay (s): 1.5 sec

Figure S25e. ^{13}C ^{1}H HMBC NMR spectrum (126 MHz, 500 MHz, DMSO) of (*E*)-azobenzene-4-[^{13}C]carboxylic acid (**4c**).

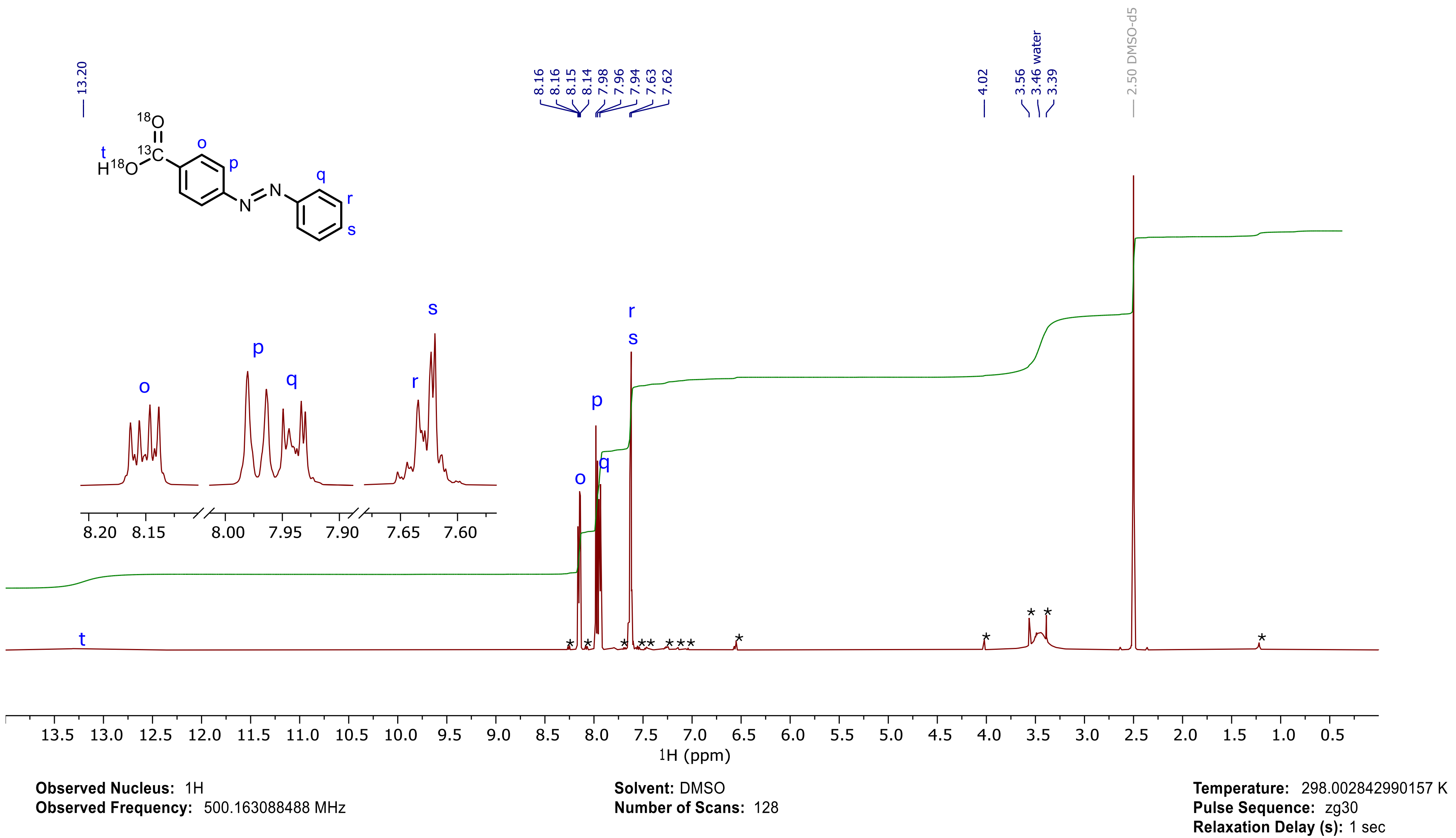

Figure S26a. ^{1}H NMR spectrum (126 MHz, DMSO) of (*E*)-azobenzene-4-[^{13}C,$^{18}O_2$]carboxylic acid (**4d**). The asterisk (*) mark signals of unidentified compounds.

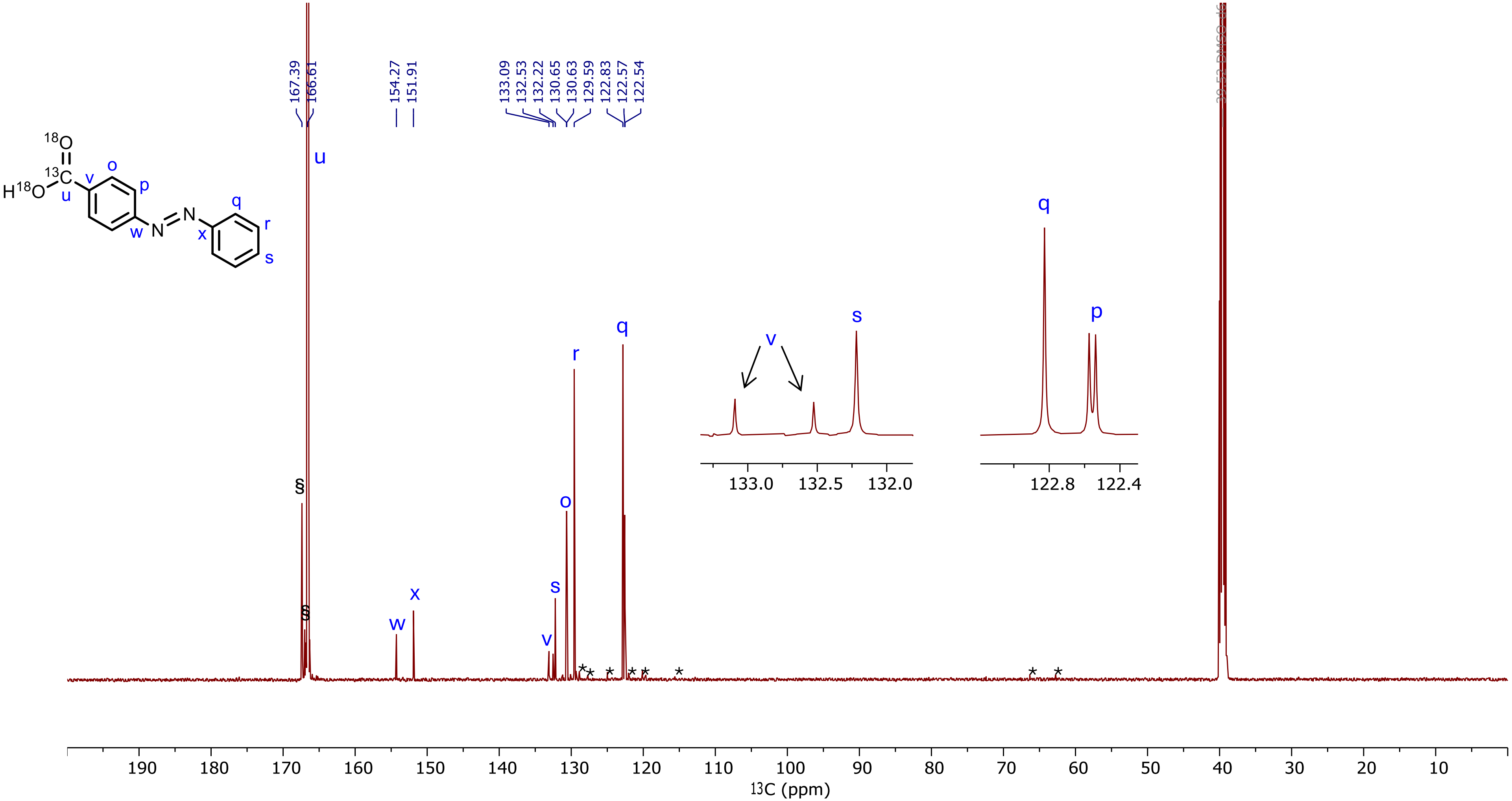

Observed Nucleus: 13C
Observed Frequency: 125.777908565 MHz

Solvent: DMSO
Number of Scans: 8192

Temperature: 297.999226984849 K
Pulse Sequence: zgpg30
Relaxation Delay (s): 2 sec

Figure S26b. $^{13}C\{^{1}H\}$ NMR spectrum (126 MHz, DMSO) of (*E*)-azobenzene-4-[^{13}C,$^{18}O_2$]carboxylic acid (**4d**). The asterisk (*) mark signals of unidentified compounds. §: We assume that these signals are caused by contaminants already present in the commercially obtained material and that these signals are only seen because of ^{13}C enrichment (see Figure S13b).

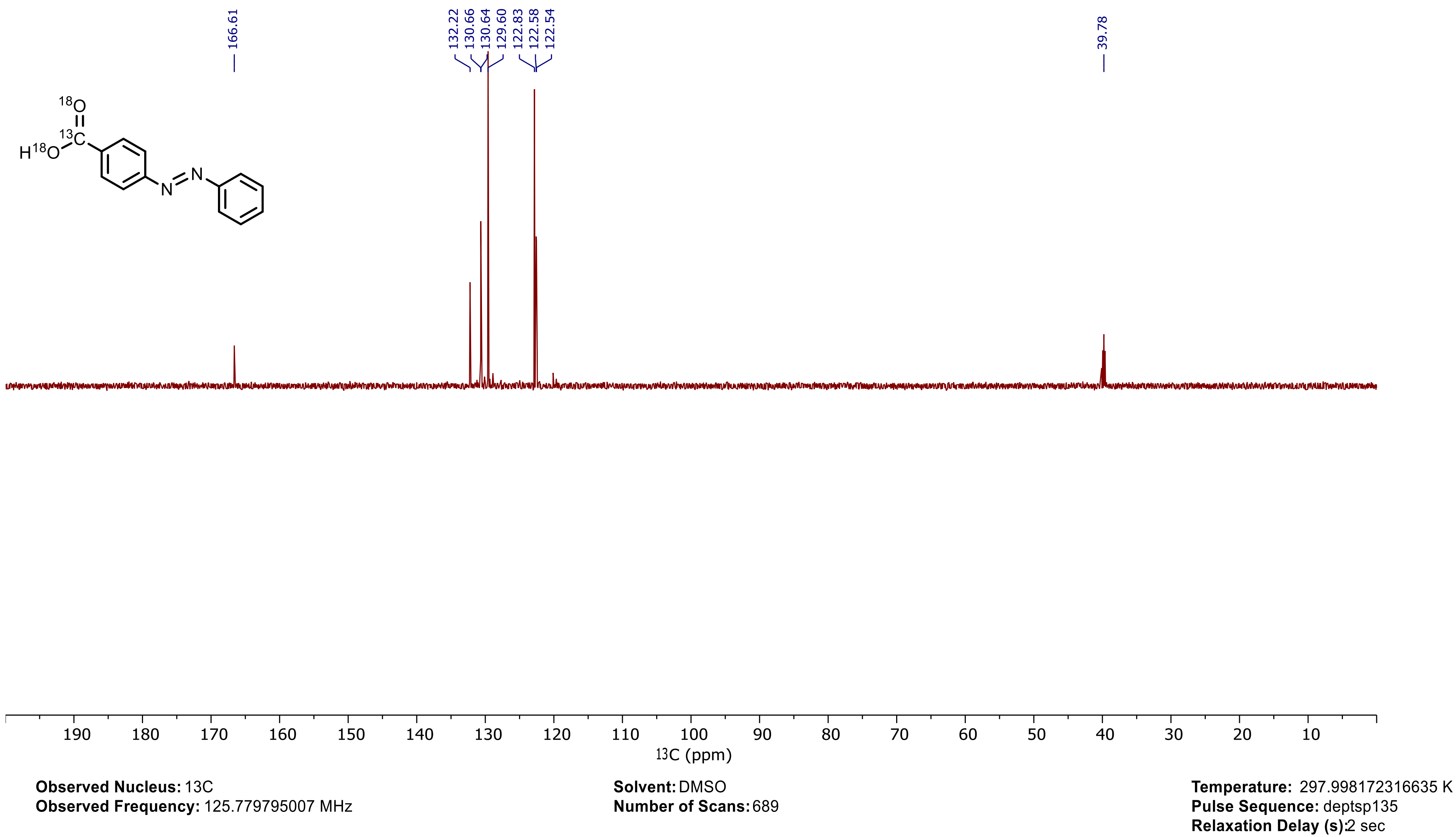

Figure S26c. ^{13}C DEPT 135 NMR spectrum (126 MHz, DMSO) of (*E*)-azobenzene-4-[^{13}C,$^{18}O_2$]carboxylic acid (**4d**).

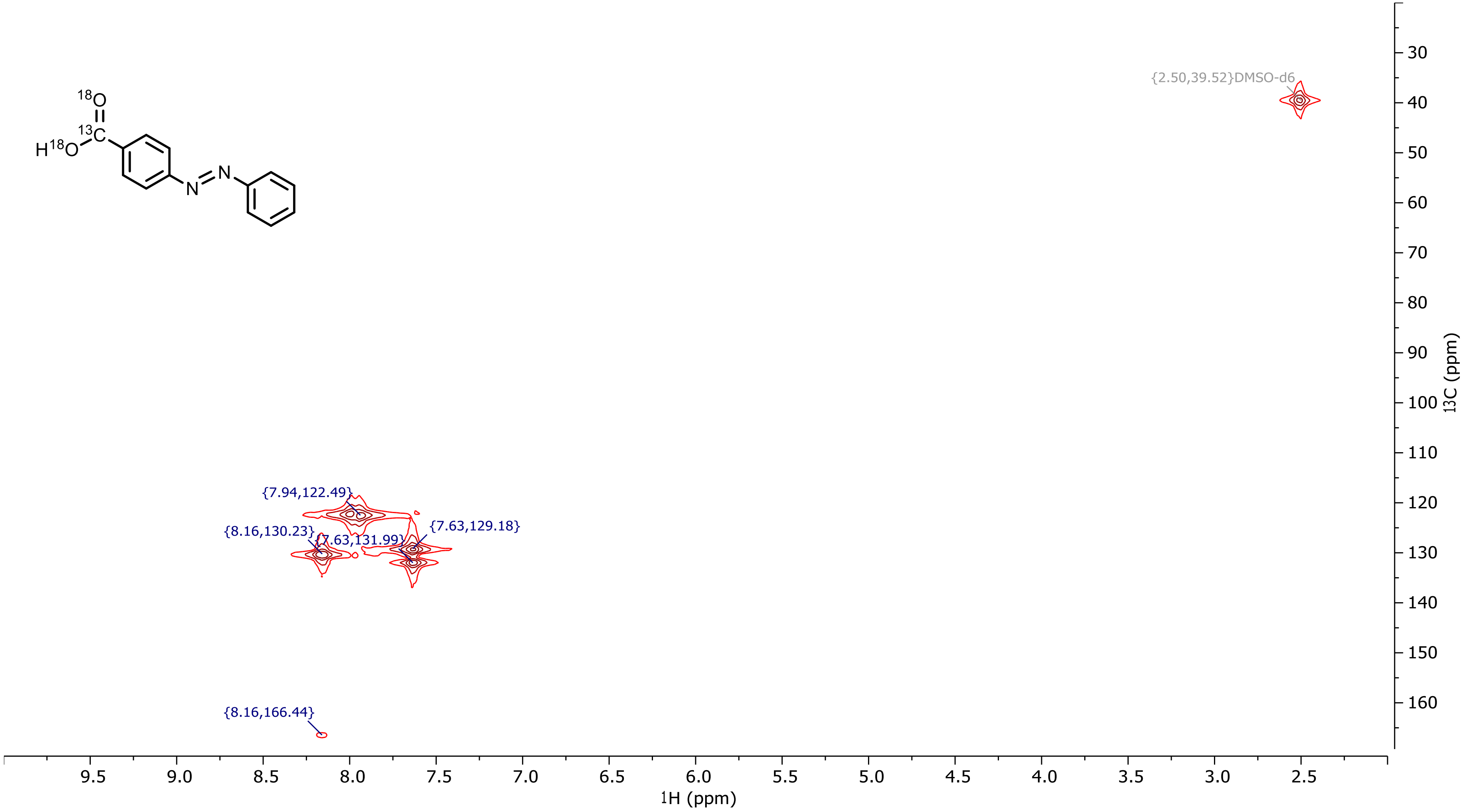

Observed Nucleus: [13C, 1H]
Observed Frequency: [125.775393258563, 500.162400768] MHz

Solvent: DMSO
Number of Scans: 8

Temperature: 298.072451092323 K
Pulse Sequence: hmqcgpqf
Relaxation Delay (s): 1.5 sec

Figure S26d. ^{13}C ^{1}H HMQC NMR spectrum (126 MHz, DMSO) of (*E*)-azobenzene-4-[^{13}C,$^{18}O_2$]carboxylic acid (**4d**).

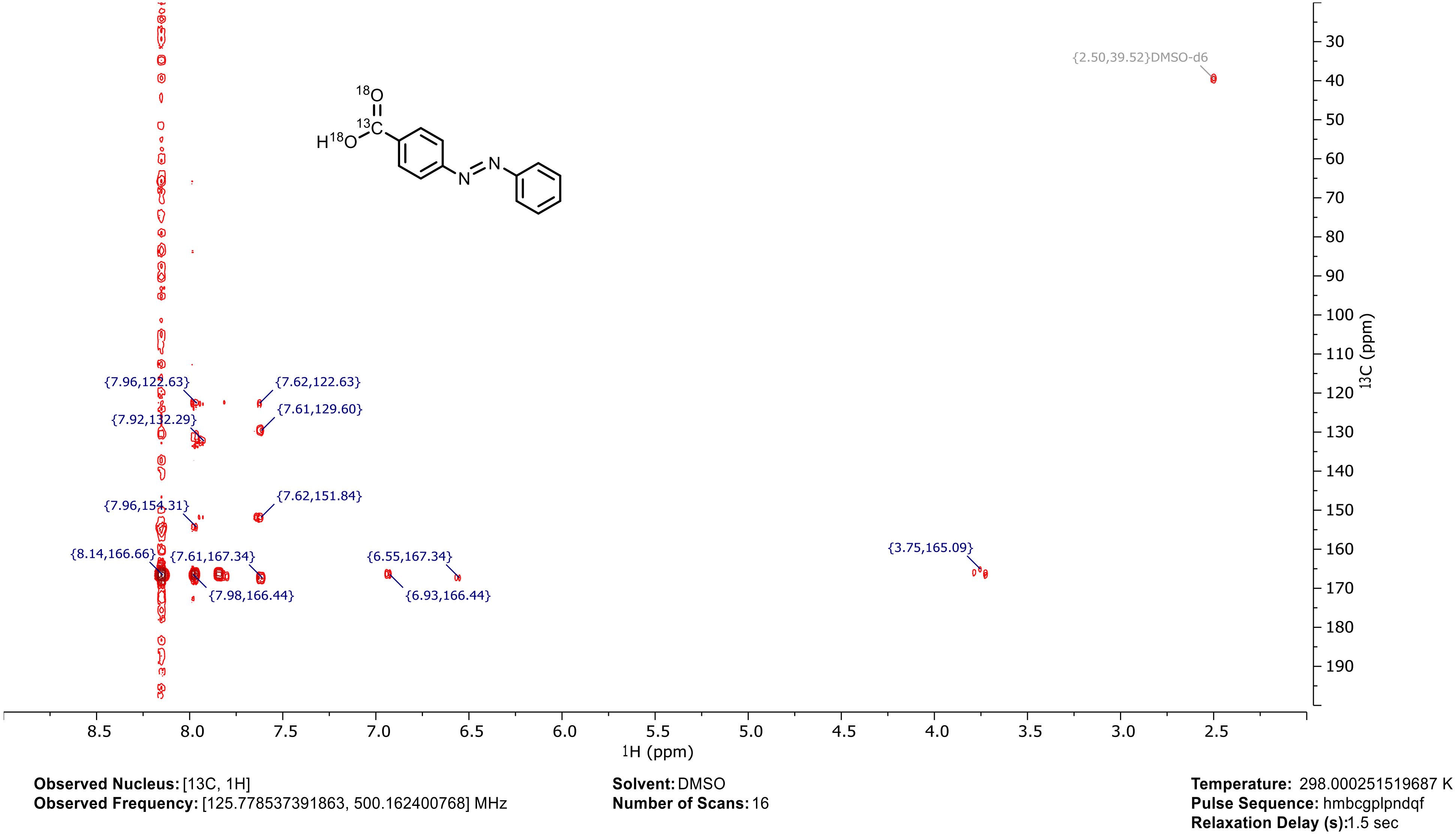

Figure S26e. ^{13}C ^{1}H HMBC NMR spectrum (126 MHz, DMSO) of (*E*)-azobenzene-4-[^{13}C,$^{18}O_2$]carboxylic acid (**4d**).

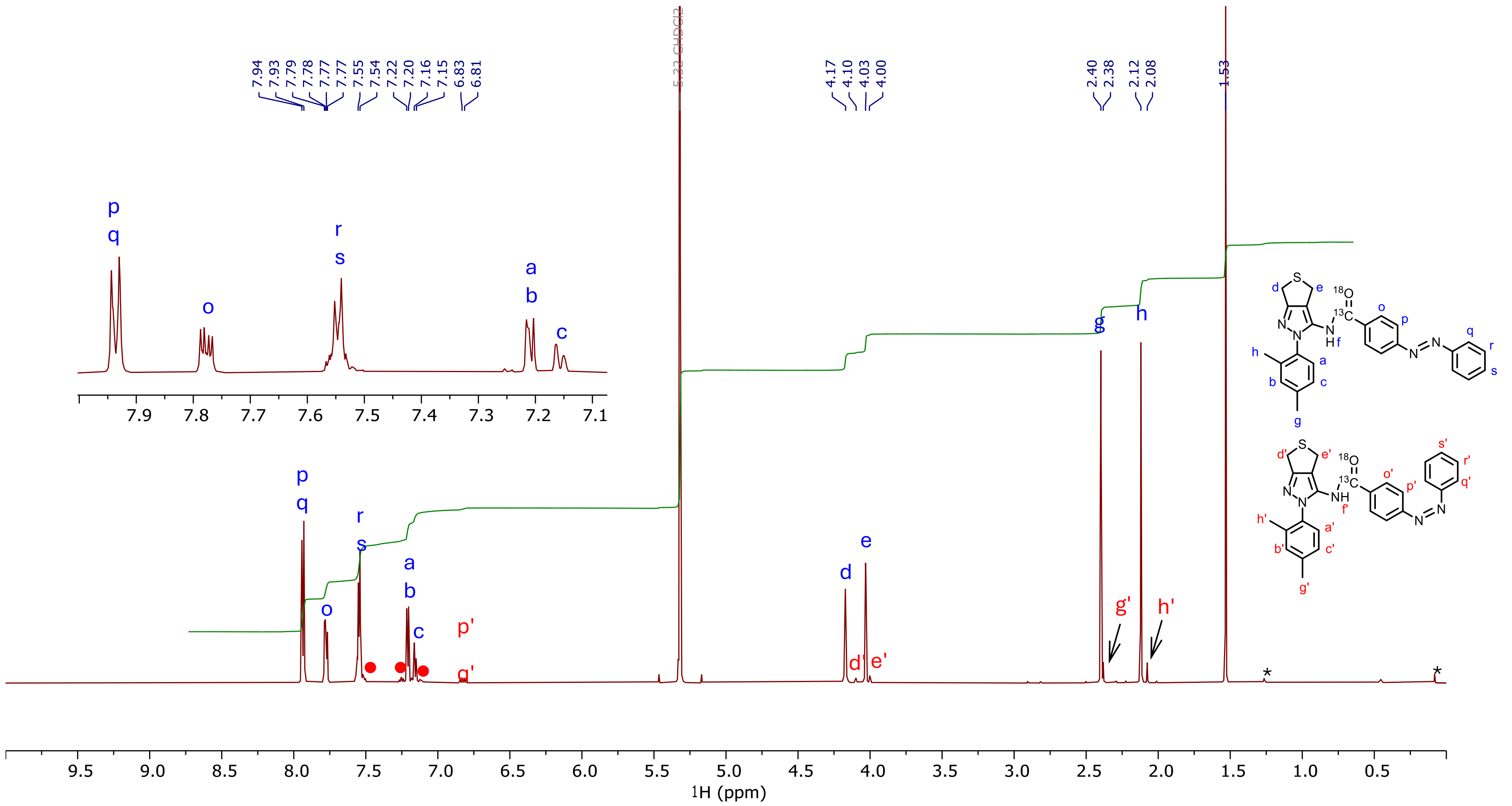

Observed Nucleus: 1H
Observed Frequency: 600.133705802 MHz

Solvent: CD2Cl2
Number of Scans: 64

Temperature: 297.998939583747 K
Pulse Sequence: zg30
Relaxation Delay (s): 1 sec

Figure S27a. ^{1}H NMR spectrum (600 MHz, CD_2Cl_2) of photopharmacological compound **1b**. The red dots (●) mark signals of the (*Z*)-isomer of photopharmacological compound **1a** and the asterisk (*) unidentified compounds.

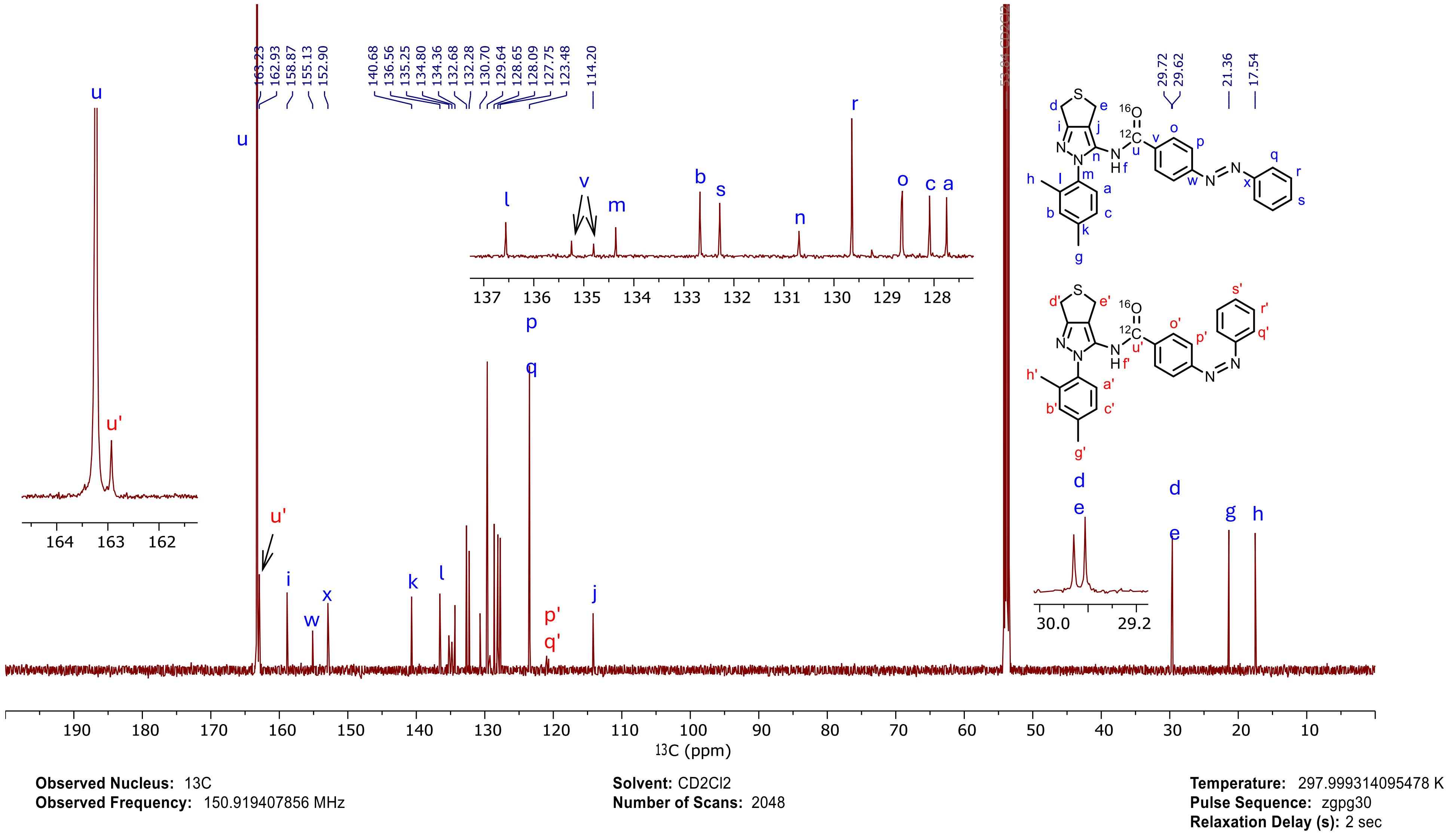

Figure S27b. $^{13}C\{^{1}H\}$ NMR spectrum (151 MHz, CD_2Cl_2) of photopharmacological compound **1b**. The red dots (●) mark signals of the (*Z*)-isomer of photopharmacological compound **1b**.

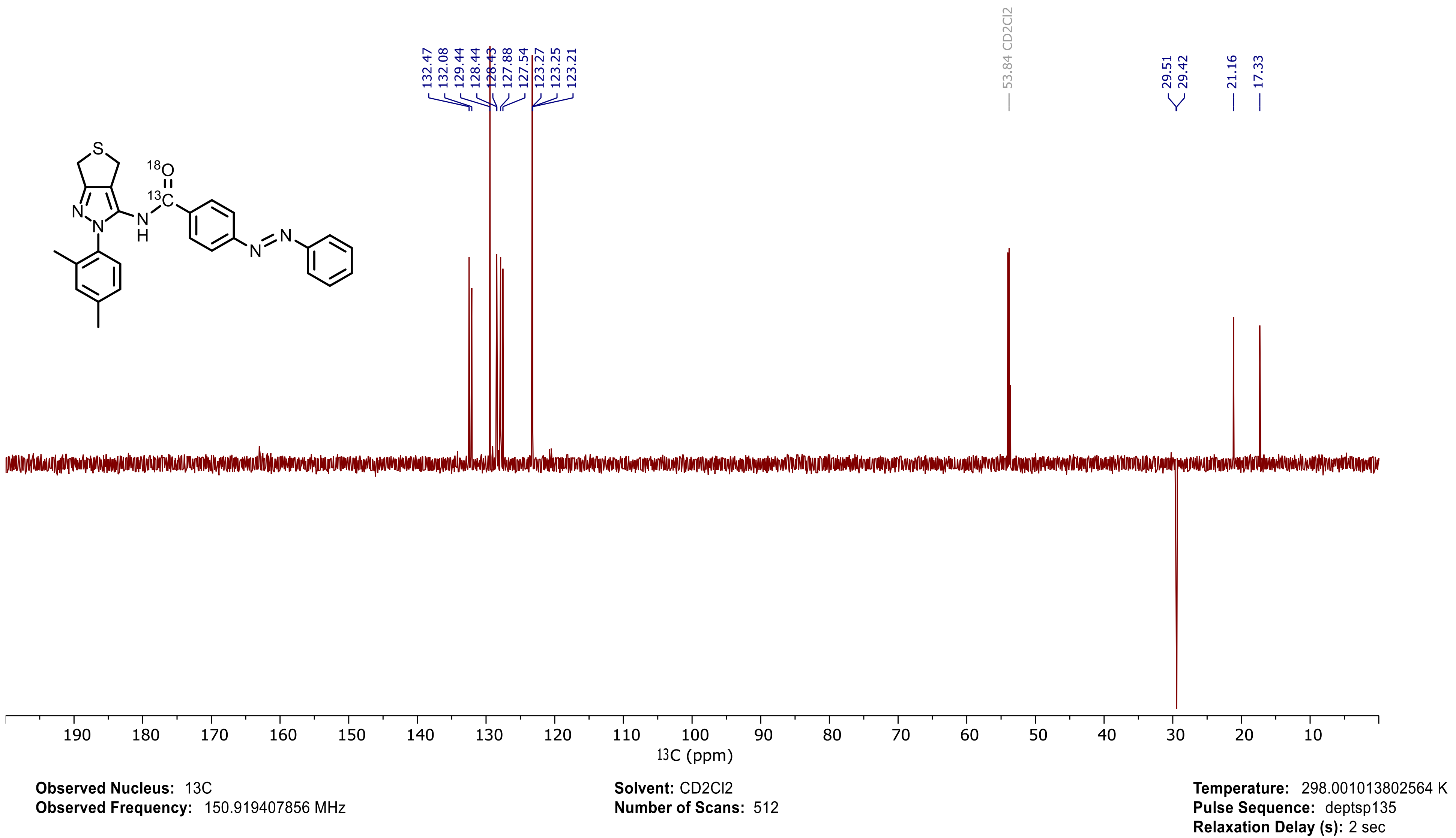

Figure S27c. ^{13}C DEPT 135 NMR spectrum (151 MHz, CD_2Cl_2) of photopharmacological compound **1b**. The red dots (●) mark signals of the (*Z*)-isomer of photopharmacological compound **1b**.

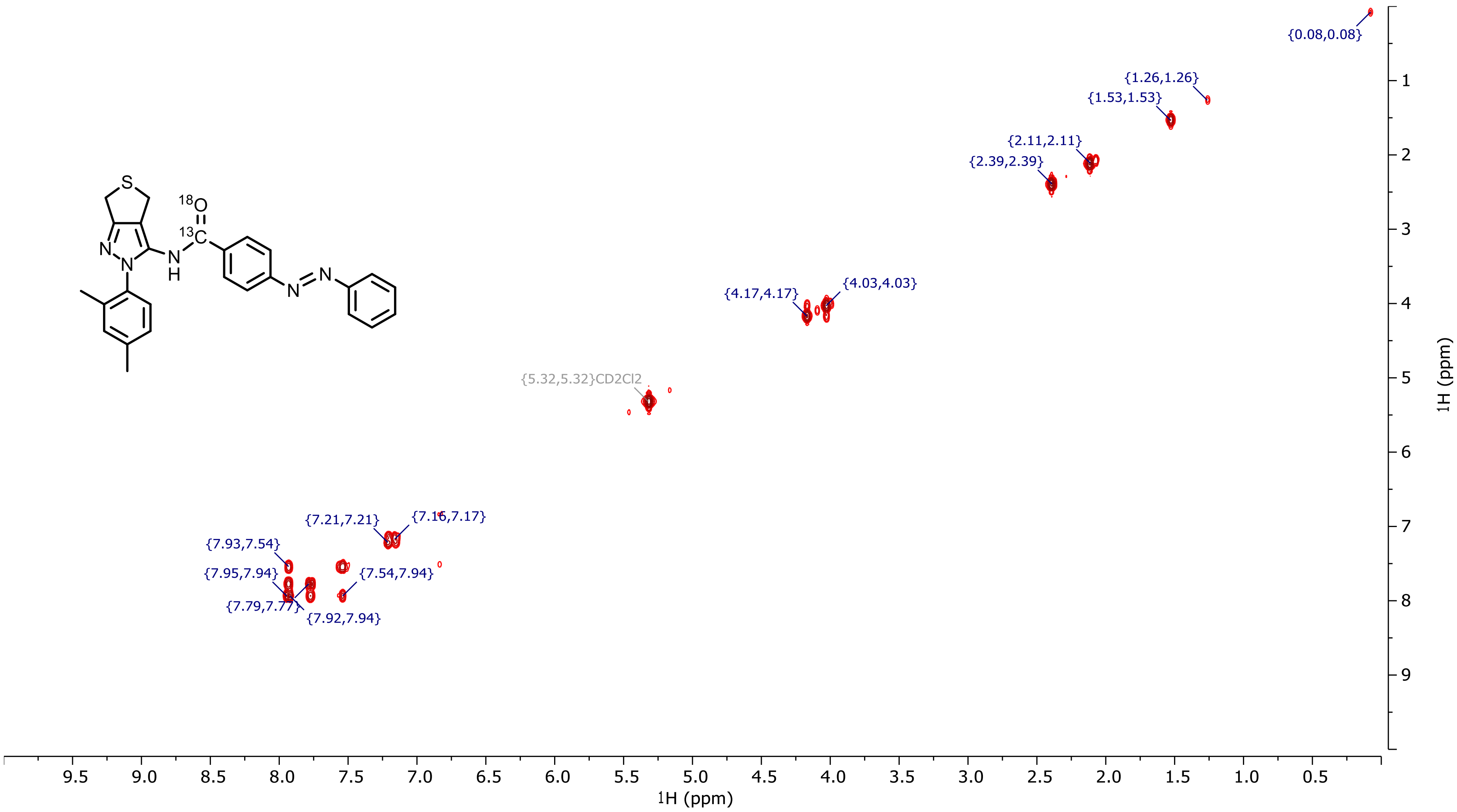

Observed Nucleus: [1H, 1H]
Observed Frequency: [600.13330072, 600.13330072] MHz

Solvent: CD2Cl2
Number of Scans: 4

Temperature: 297.999630990019 K
Pulse Sequence: cosygpppqf
Relaxation Delay (s): 2 sec

Figure S27d. ^{1}H ^{1}H COSY NMR spectrum (600 MHz, 600 MHz, CD_2Cl_2) of photopharmacological compound **1b**.

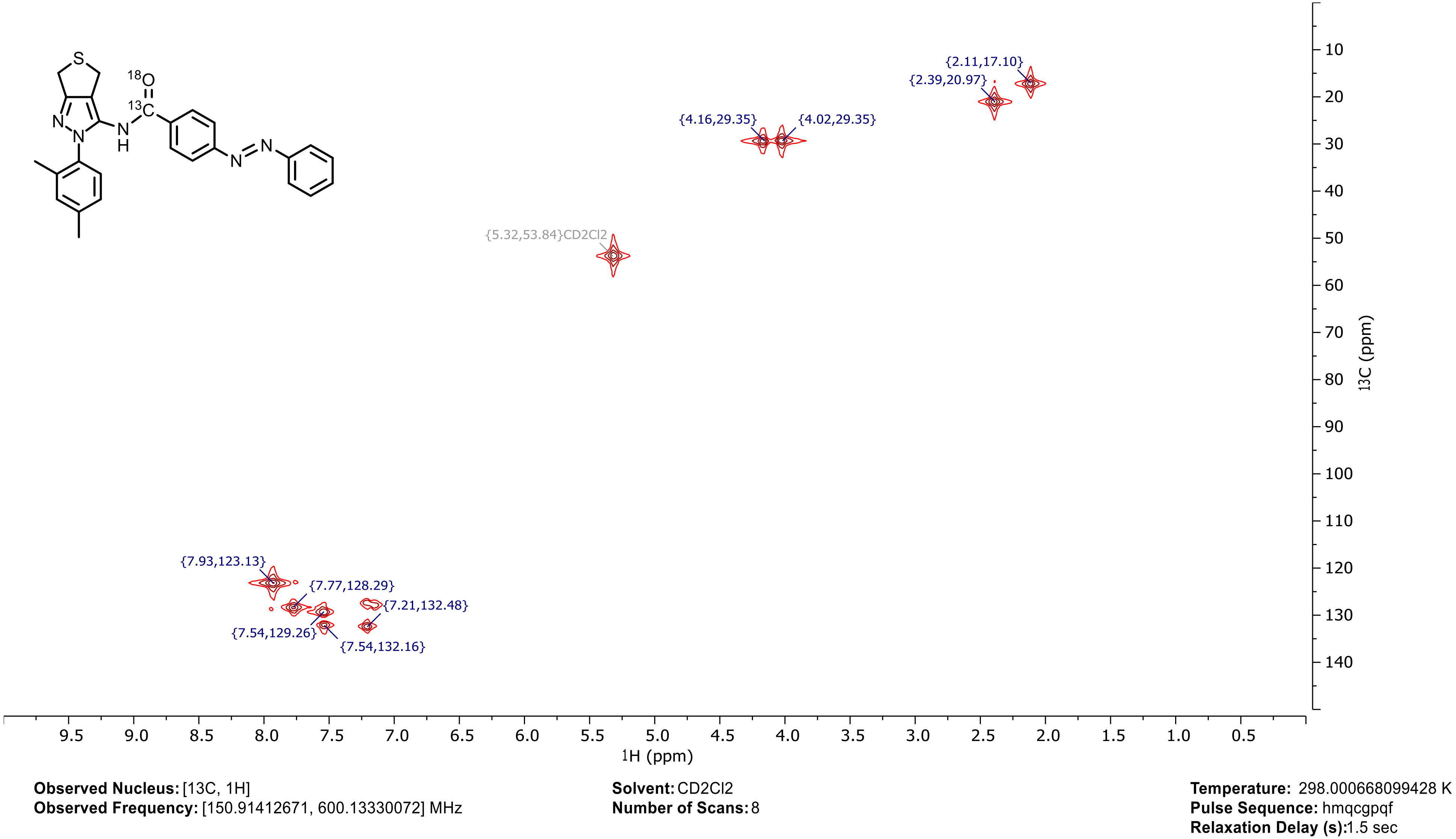

Figure S27e. ^{13}C ^{1}H HMQC NMR spectrum (151 MHz, 600 MHz, CD_2Cl_2) of photopharmacological compound **1b**.

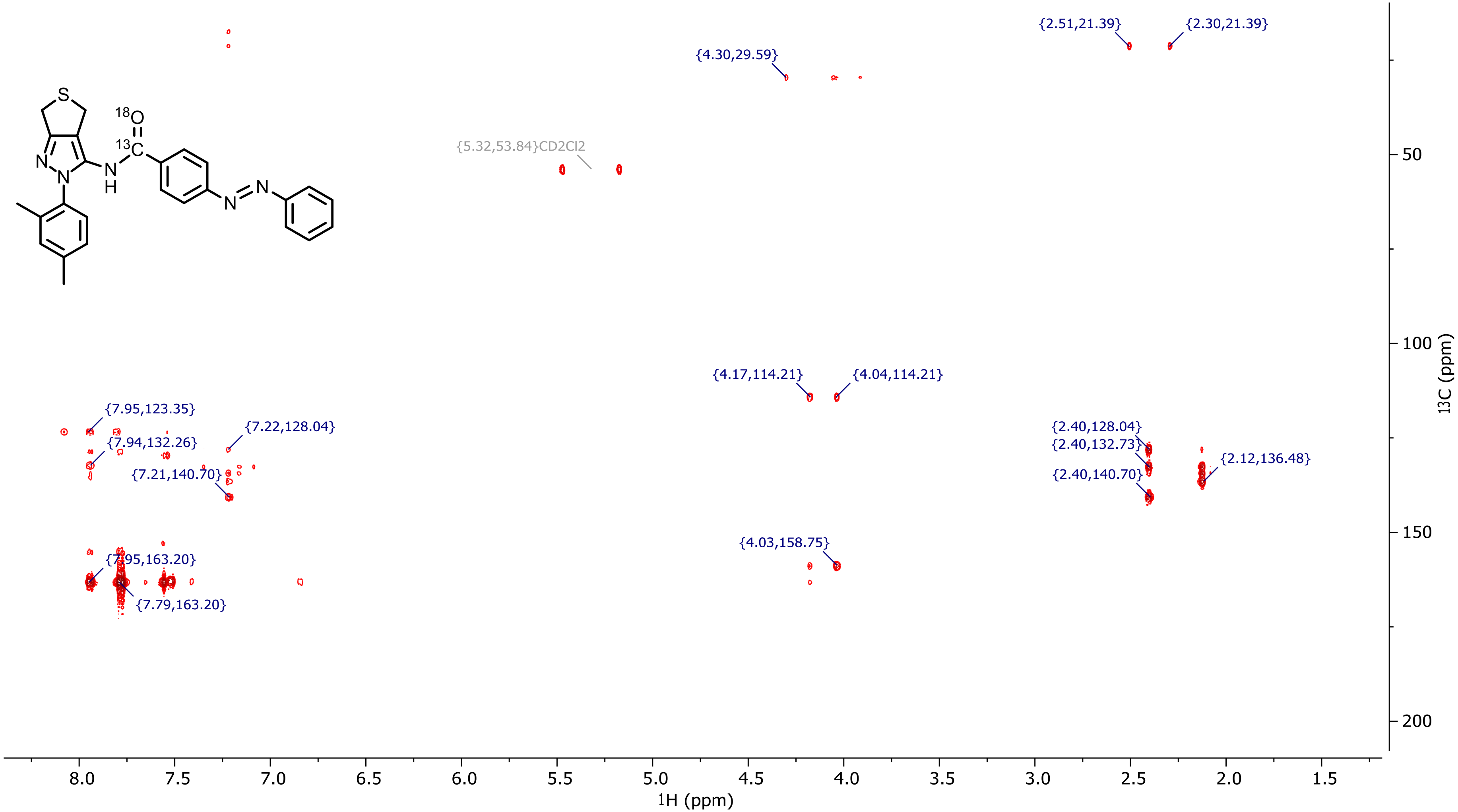

Observed Nucleus: [13C, 1H]
Observed Frequency: [150.92016282, 600.13330072] MHz

Solvent: CD2Cl2
Number of Scans: 16

Temperature: 297.999227669694 K
Pulse Sequence: hmbcgpndqf
Relaxation Delay (s): 1.5 sec

Figure S27f. ^{13}C ^{1}H HMBC NMR spectrum (151 MHz, 600 MHz, CD_2Cl_2) of photopharmacological compound **1b**.

References for Experimental Procedures, MS Spectra & NMR Spectra

(1) Wang, B.; Jorns, M. S., Reconstitution of *Escherichia coli* DNA photolyase with various folate derivatives. *Biochemistry* **1989,** *28* (3), 1148-1152.

(2) Macheroux, P., UV-visible spectroscopy as a tool to study flavoproteins. *Methods Mol. Biol.* **1999,** *131* 1-7.

(3) Varadi, M.; Bertoni, D.; Magana, P.; Paramval, U.; Pidruchna, I.; Radhakrishnan, M.; Tsenkov, M.; Nair, S.; Mirdita, M.; Yeo, J.; Kovalevskiy, O.; Tunyasuvunakool, K.; Laydon, A.; Zídek, A.; Tomlinson, H.; Hariharan, D.; Abrahamson, J.; Green, T.; Jumper, J.; Birney, E.; Steinegger, M.; Hassabis, D.; Velankar, S., AlphaFold protein structure database in 2024: providing structure coverage for over 214 million protein sequences. *Nucleic Acids Res.* **2023,** *52* (D1), D368-D375.

(4) Seyfried, M. S.; Lauber, B. S.; Luedtke, N. W., Multiple-turnover isotopic labeling of Fmoc- and Boc-protected amino acids with oxygen isotopes. *Org. Lett.* **2010,** *12* (1), 104-106.

(5) Marecek, J.; Song, B.; Brewer, S.; Belyea, J.; Dyer, R. B.; Raleigh, D. P., A simple and economical method for the production of C, O-labeled Fmoc-amino acids with high levels of enrichment: Applications to isotope-edited IR studies of proteins. *Org. Lett.* **2007,** *9* (24), 4935-4937.

(6) Letzel, M. *Universal Mass Calculator (UMC) for Windows*, 2021.

(7) Dantzig, G. B., *Linear programming and extensions*. Princeton University Press: 1991.

(8) Kagechika, H.; Himi, T.; Namikawa, K.; Kawachi, E.; Hashimoto, Y.; Shudo, K., Retinobenzoic acids. 3. Structure-activity relationships of retinoidal azobenzene-4-carboxylic acids and stilbene-4-carboxylic acids. *J. Med. Chem.* **1989,** *32* (5), 1098-1108.

(9) Kolarski, D.; Miller, S.; Oshima, T.; Nagai, Y.; Aoki, Y.; Kobauri, P.; Srivastava, A.; Sugiyama, A.; Amaike, K.; Sato, A.; Tama, F.; Szymanski, W.; Feringa, B. L.; Itami, K.; Hirota, T., Photopharmacological Manipulation of Mammalian CRY1 for Regulation of the Circadian Clock. *J. Am. Chem. Soc.* **2021,** *143* (4), 2078-2087.

(10) Miller, S.; Son, Y. L.; Aikawa, Y.; Makino, E.; Nagai, Y.; Srivastava, A.; Oshima, T.; Sugiyama, A.; Hara, A.; Abe, K.; Hirata, K.; Oishi, S.; Hagihara, S.; Sato, A.; Tama, F.; Itami, K.; Kay, S. A.; Hatori, M.; Hirota, T., Isoform-selective regulation of mammalian cryptochromes. *Nat. Chem. Biol.* **2020,** *16* (6), 676-685.
